# Pottery spilled the beans: Patterns in the processing and consumption of dietary lipids in Central Germany from the Early Neolithic to the Bronze Age

**DOI:** 10.1371/journal.pone.0301278

**Published:** 2024-05-16

**Authors:** Adrià Breu, Roberto Risch, Elena Molina, Susanne Friederich, Harald Meller, Franziska Knoll

**Affiliations:** 1 Department of Prehistory, Autonomous University of Barcelona, Barcelona, Catalonia, Spain; 2 Department of Social Sciences and Humanities, Koç University, Istanbul, Türkiye; 3 State Office for Heritage Management and Archaeology Saxony-Anhalt, Halle (Saale), Germany; New York State Museum, UNITED STATES

## Abstract

The need to better understand economic change and the social uses of long-ago established pottery types to prepare and consume food has led to the study of 124 distinct ceramic vessels from 17 settlement and funerary sites in Central Germany (present day Saxony-Anhalt). These, dated from the Early Neolithic (from 5450 cal. BCE onwards) to the Late Bronze Age (1300–750 cal. BCE; youngest sample ca. 1000 BCE), include vessels from the Linear Pottery (LBK), Schiepzig/Schöningen groups (SCHIP), Baalberge (BAC), Corded Ware (CWC), Bell Beaker (BBC), and Únětice (UC) archaeological cultures. Organic residue analyses performed on this assemblage determined the presence of vessel contents surviving as lipid residues in 109 cases. These were studied in relation to the changing use of settlement and funerary pottery types and, in the case of burials, to the funerary contexts in which the vessels had been placed. The obtained results confirmed a marked increase in the consumption of dairy products linked to innovations in pottery types (e.g., small cups) during the Funnel Beaker related Baalberge Culture of the 4^th^ millennium BCE. Although the intensive use of dairy products may have continued into the 3^rd^ millennium BCE, especially amongst Bell Beaker populations, Corded Ware vessels found in funerary contexts suggest an increase in the importance of non-ruminant products, which may be linked to the production of specific vessel shapes and decoration. In the Early Bronze Age circum-Harz Únětice group (ca. 2200–1550 BCE), which saw the emergence of a highly hierarchical society, a greater variety of animal and plant derived products was detected in a much more standardised but, surprisingly, more multifunctional pottery assemblage. This long-term study of lipid residues from a concise region in Central Europe thus reveals the complex relationships that prehistoric populations established between food resources and the main means to prepare, store, and consume them.

## Introduction

The first agricultural and pottery-producing societies settled in Central Europe around 7,500 years ago as part of the so-called Early Neolithic Linear Pottery (LBK) expansion. Over the following millennia an exceptional cultural diversity unfolded in the region, an aspect of which was expressed through a wide range of pottery styles and decorations. These, in combination with burial practices, have been essential to the definition of the *archaeological cultures* that still today structure the prehistoric materiality of large parts of Europe into chronological and spatially recognisable entities [1]. Whatever ideological meanings might have been expressed through these clearly recognisable pottery types, they were manufactured to fulfil everyday needs, probably as means to store, cook, and serve food. To a large extent, pottery would have been a manufactured object mediating between the available subsistence resources and human consumption, both at the individual and at the social level. In this material sense, and as means to transform subsistence resources into nourishment, different pottery forms can be expected to have reflected changing human-animal-plant relationships. The study of vessel contents thus may provide crucial insights into Central Germany’s prehistoric economies as well as into social practices of food consumption and sharing [2–7].

The main aim of the present study has been to determine the importance of various products within the pottery-based food storing and culinary practices of Central Germany throughout the Neolithic and the Bronze Age (ca. 5500–1000 BCE) by studying the lipid residues trapped in a set of 124 period-characteristic pottery vessels of different shapes, sizes, and contexts. Lipid analyses can distinguish between residual fats derived from milk, ruminant and non-ruminant animals, as well as of marine or plant origin [8–11], or the effects of heating [12–16]. Their detection has been used as a proxy for the presence of wider food categories such as dairy [2,17] or plant products [11,18] and as direct evidence for the sources of fat used in those foodways involving pottery. Although previous lipid studies in Central Europe are available, these have focused mainly on Early Neolithic contexts [4,6,19,20], resulting in an absence of geographically well-defined diachronic analyses exploring connections between different pottery types, sizes, decorations, the depositional context of the vessels, and their different types of residues.

In the continental but at the same time relatively arid conditions of Central Germany from the Holocene Thermal Maximum to the 4.2kyr event [21–24], the consumption of lipids through fat-rich food may have been more critical for human nourishment than in more temperate and southern regions, where a broader array of plant resources was available since the beginning of the Neolithic [25,26]. Amongst the existing lipid-rich resources in the Central German Neolithic, the existing faunal analyses show that cattle were the most important domesticate across time while sheep, goat, and pig were of secondary importance and hunting played only a minor role [27]. Flax could have also been an alternative source of fat-rich meals, as its presence is attested since the Early Neolithic of Central Germany, either through seeds or as textiles [28–30]. However, as important as the differing fat resources used during Later Prehistory are, the pottery containers in which they were processed, stored, and offered for consumption can also point at the social rules and norms ordering their distribution. In light of the dairy-centred research by Evershed et al. [19], where cultural changes in dietary preferences are considered as a potential factor to explain past change, integrating pottery characteristics with additional contextual and social information has become indispensable. Thus, the research presented here aims to shed new light on several major social and economic changes observed in later prehistoric Europe, some of which have been the focus of recent debates and studies:

1. How did the exploitation of ruminant animals for dairy products change from the Early Neolithic to the Late Bronze Age, i.e. between ca. 5500 to ca. 1000 BCE? Dairy products are present in 7^th^ millennium pottery vessels used by northern Mesopotamian farmers [31,32], but their abundance in the Mediterranean Early Neolithic is low [33–37]. A recent European-wide analysis of the correlation between dairy fats and the lactase persistence gene indicates that the preparation and consumption of dairy products is not a consequence of the expansion of the lactase persistence gene [19] but a socioeconomic feature of only certain Neolithic and Bronze Age groups. Was the increase in dairy products accompanied by the appearance of specialised vessel types? The up to 100 hectares of ditched enclosures (“Erdwerke”) defining the landscape of Central Europe during the 4^th^ millennium and within the Michelsberg Culture have been interpreted as possible cattle corrals beyond other symbolic or political relationships [e.g., 38–40]. Although these suggest an increased capability to produce dairy products, it is not clear what role pottery played in supporting the new animal-human relationships in contrast to the apparently more agriculturally oriented Early Neolithic communities.
2. Archaeogenetics has identified a dominant Eastern European or so-called “steppe” ancestry component among communities using Corded Ware (CWC) pottery from ca. 2800 BCE onwards [41,42]. The rapid expansion of these groups throughout large parts of Europe has been explained by their alleged nomadic or pastoral economy. Co-existing Corded Ware and Bell Beaker (BBC) communities in Central Germany (2500–2200 cal. BCE) followed similar individual burial practices but showed marked differences in settlement location and architecture which confer a more husbandry-oriented milieu to the Corded Ware groups in contrast to a greater importance of agriculture among the Bell Beaker groups [43]. Can these differences also be detected in the way pottery was used by these communities, particularly in their burial rituals?
3. During the Early Bronze Age, identified with the circum-Harz Únětice group (UC), a much more complex form of social organisation emerged. Evidence of marked social differences, probably hereditary, labour specialisation, mainly in the realm of metallurgy and gold working, settlement aggregation and surplus production through a dependant work force are recognised as traits of early state formation [44–46]. These political and economic changes could have influenced diets and pottery-based foodways, as suggested by the appearance of a new, rather standardised and undecorated set of vessel types.

This study explores these three issues of later prehistoric archaeology within the geographical context of modern-day Saxony-Anhalt by analysing the relationship between vessel contents and pottery forms through time. Moreover, the domestic versus funerary character of the final vessel placement will be introduced as a third variable. Pottery used as grave goods in burials provides an even closer insight into the relationship of specific individuals, of different sex and age, to pottery forms and their contents.

## Materials and methods

### Sampling

**Fig 1:**
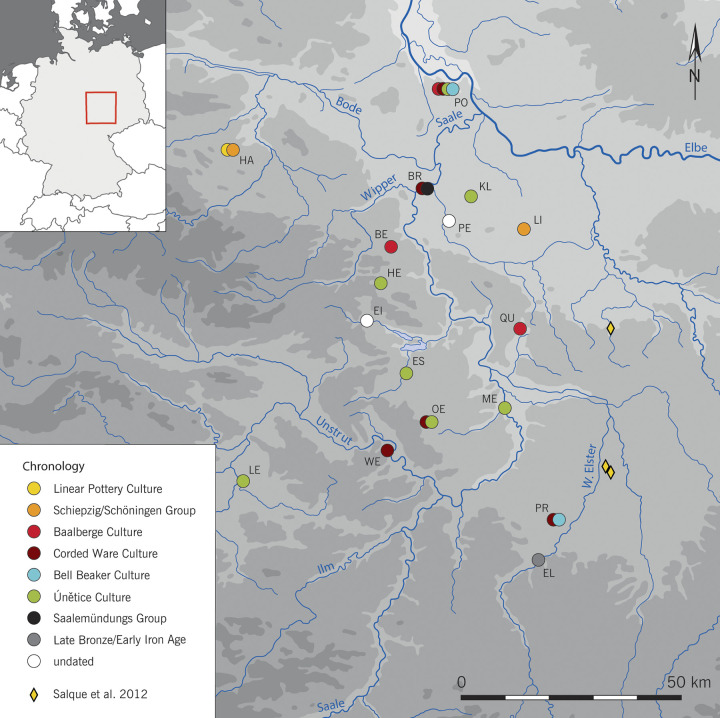
Location and chronology of the pottery discussed in this paper or in previous studies [4].

For this first comprehensive diachronic study of prehistoric organic residues in the Saxony-Anhalt region, 124 distinct and well-preserved ceramic vessels from 17 archaeological sites dating from the Early Neolithic (LBK—5450 cal. BCE) to the beginning of the Early Iron Age, in the first half of the 1^st^ millennium BC, were sampled (see Table 1 and S1 Fig). Samples from a broad range of vessel types (for drinking, storing, food preparation), archaeological contexts (settlement/household, grave goods, deposits) were included (Table 1, and Fig 1). In selected cases, more than one sample was taken per vessel to validate results with different extraction methods (S1 Table) or to test different vessel parts, resulting in the total preparation and analysis of 199 samples.

**Table 1:** Site names, chronologies, degree of preservation, and archaeological context of the vessels sampled.

Period	Culture/ Group	Period chronology (cal. BCE)	Site name	Context			Preservation rate (%)
				Settlement	Funerary	Deposit	
Early Neolithic (EN)	Linear Pottery (LBK)	5450–4775 [47 page 47]	Harsleben (HA)	2	5		100
Middle Neolithic (MN)	Schiepzig/ Schöningen groups (SCHIP)	4200–3800 [48 page 121]	Harsleben (HA)	7			100
			Libehna (LI)	5			100
	Baalberge (BAC)	3950–3375 [49]	Queis (QU)		10		90
			Belleben (BE)	11			100
			Pömmelte (PO)		5		40
Late Neolithic (LN)	Corded Ware, Phases 1b–3 (CWC)	2575–2200 [50 page 672]	Wennungen (WE)		3		100
			Profen (PR)		16		100
			Oechlitz (OE)		7		100
			Pömmelte (PO)			1	100
			Bernburg (BR)		1		100
	Bell Beaker (BBC)	2500–2200 [50]	Pömmelte (PO)		8		100
			Profen (PR)		2		100
Early Bronze Age (EBA)	Únětice, Phases 1–3 (UC)	2200–1775 [51, page 222]	Pömmelte (PO)	2	2	10	35
			Oechlitz (OE)		4		100
			Kleinpaschleben (KL)	9			100
			Merseburg (ME)	5			100
			Esperstedt (ES)	2			100
			Leubingen (LE)		1		0
			Helmsdorf (HE)		1		0
Late Bronze Age (LBA)	Early Saalemündung group (Ha B1)	1075–1000	Bernburg (BR)		2		100
Reference samples	Elsteraue (EL) LBA/Iron Age			1			100
	Eisleben (EI)			1			100
	Preußlitz (PE)			1			100
Total				46	67	11	88

Pottery sampled: “Settlement” includes domestic, production, and storage areas. “Funerary” encompasses grave goods found *in situ*. “Deposit” reflects unexpected concentrations of artefacts within a site. Preservation rate: percentage of samples with a total lipid extract higher than 5 μg g^-1^. Reference samples correspond to pottery used to assess the extent of contamination caused by the 2013 floods of the river Saale, see S1 File, and have not been used for archaeological interpretations.

The sampling strategy mainly focused on studying pottery coming from recent excavations of the State Office for Heritage Management and Archaeology Saxony-Anhalt, recovered both in the field but also from *Blockbergungen*, i.e., burials recovered *in situ* in their sedimentary context and later excavated in the laboratories of the State Office, ensuring the recovery of parts of vessels in their best possible condition. A special focus was placed on the transition from the Early Neolithic (EN) to the Middle Neolithic (MN) and from the Late Neolithic (LN) to the Early Bronze Age (EBA), while sampling was more limited for the LBK in the 6^th^ and 5^th^ millennium BCE. As a corpus of settlement data for this period and region is already available [4,19], mostly LBK funerary vessels, which are quite rare in this part of Central Europe, were analysed. Two Late Bronze Age funerary vessels from Bernburg were studied as part of the analyses of several graves from this site, but, due to their singularity, they should not be considered as a representative sample of the pottery used in this period. Finally, three undated pottery sherds were chosen as reference samples from areas of the museum storage rooms undamaged by the ingressed 2013 Saale river flood, in order to assess to what extent certain vessels from *Blockbergungen* may have been affected and guide the sampling strategy of this study.

### Period-specific sampling details

The arrival of the first farmers and herders in the region in the 6^th^ millennium BCE is represented by the Linear Pottery Culture (LBK), which is widespread across Central Europe. In Harsleben (HA), the northernmost site in the study, two sampled vessels from the settlement and five from three burials belong to this period. Our diachronic sampling continued in the early Middle Neolithic with 12 pottery vessels from the sites of Harsleben (HA) and Libehna (LI), the latter of which was protected by an earthwork [52]. Both sites belong, respectively, to the local groups of Schöningen [53] and Schiepzig (4200–3800 cal. BCE) (SCHIP), which can be described as Epi-Rössen/Lengyel phenomena [cf. 48] with influences from the Michelsberg Culture [54]. In the first half of the 4^th^ millennium BCE, a demographic shift was brought about by the Middle Neolithic Funnel Beaker cultures, represented in this study by the Baalberge Culture (BAC). Samples from 26 vessels were analysed, of which 11 correspond to the ditched enclosure of Belleben (BE) [55], while another 15 come from four graves inside a typical trapezoidal ditch at Queis (QU) [56] and different funerary contexts at Pömmelte (PO) [57]. At the beginning of the Late Neolithic (early 3^rd^ millennium), the arrival of populations from the eastern steppes configured the Corded Ware Culture (CWC). Samples from 28 pots placed in burials at Wennungen (WE), Profen (PR), Oechlitz (OE), and Bernburg (BR), dated not before ca. 2575 cal. BCE, were studied from this period. Due to the scarcity of Late Neolithic settlement evidence in this region, only one additional vessel, a miniature cup deposited inside a small pit, could be sampled at Pömmelte (PO). The same situation applies to the Late Neolithic (LN) Bell Beaker Culture (BBC), represented here by ten samples from burials in Pömmelte and Profen dating between 2500 and 2200 cal. BCE.

**Fig 2:**
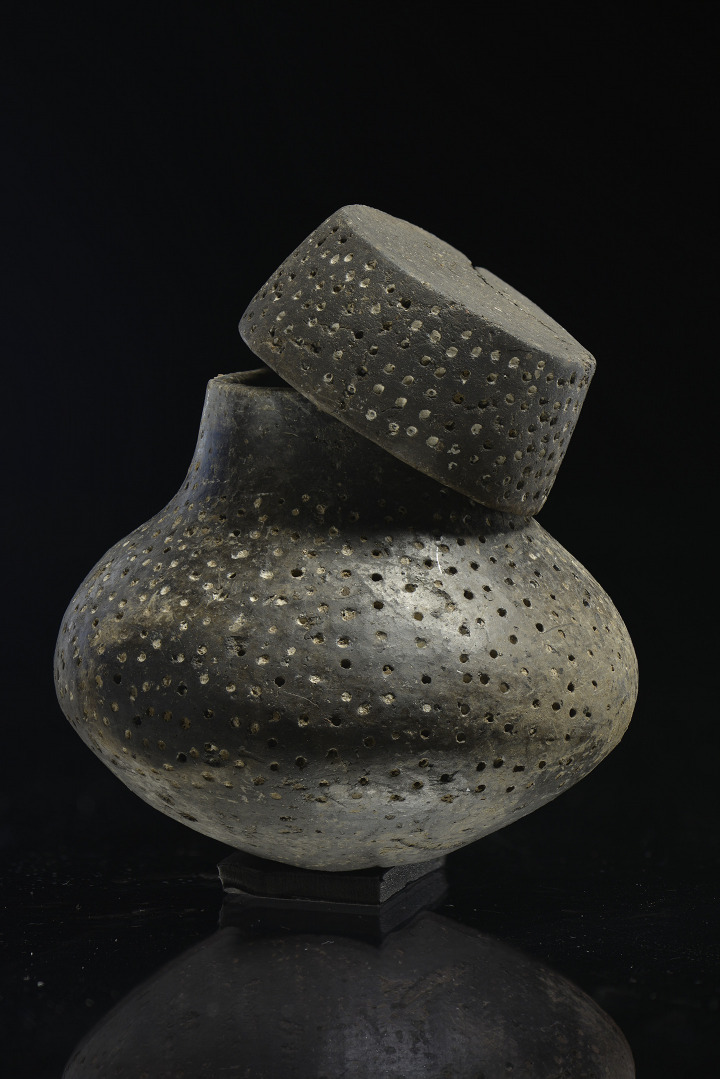
Funerary vessel with lid from Oechlitz. In this case, the vessel (OE-10) and a residue visible on the interior (OE-20) were sampled.

The onset of the Bronze Age is marked in Central Europe by the Únětice Culture (UC), which bore close genetic links to the Corded Ware as well as to the Bell Beaker populations [41]. At a time with increased social differentiation, two large storage vessels from the princely tombs of Helmsdorf (HE) and Leubingen (LE) [cf. 58], and a very small vessel with a lid, found in a rich burial at Oechlitz [59] (Fig 2) were included in the study [60]. A further three funerary vessels were analysed from Únětice burials excavated in Oechlitz (OE) and Pömmelte (PO). In this period, the settlement’s pottery is represented by 28 samples from Kleinpaschleben (KL) [61], Merseburg (ME), Esperstedt (ES) [62], and Pömmelte (PO). Most of the vessels (ten in number) from the latter site were found together in an exceptional hoard find. Finally, two funerary urns from Bernburg, belonging to the Late Bronze Age Early Saalemündung group (Ha B1), were analysed to gain initial insights into the pottery use of later prehistoric periods.

### Methods

To perform the analyses, the surface of each sherd was removed using a modelling drill to eliminate exogenous lipids, traces of soil from the burial environment, and contamination from handling. Approximately 2 g of each sherd were pulverised with a pestle and mortar and stored in a cold room in glassware or aluminium foil, which had been fired at 450°C for 8 h, prior to sample extraction. *N*-tetratriacontane (40 μL of a 548 ng μL^-1^ solution in isooctane) was added to all samples for quantification of target analytes and to appraise their analytical recoveries before extraction with commonly applied Dichloromethane/Methanol (DCM/MeOH) or Acidified Methanol procedures (see S1 File for specific procedure details) [3,63,64].

For samples from Harsleben, Libehna, Wennungen, Profen, Oechlitz, Bernburg, Kleinpaschleben, Leubingen, Helmsdorf, Elsteraue, and Eisleben, when the DCM/MeOH procedure did not yield significant amounts of lipids (TLE < 5 μg g^-1^), a second extraction with Acidified Methanol was performed on the same 2 g of sample. Samples from Queis, Belleben, Pömmelte, Merseburg, Esperstedt, and Preußlitz were directly extracted with the Acidified Methanol procedure.

After their extraction, residues were screened for lipids by Gas Chromatography Flame Ionisation Detection (GC-FID) and samples selected according to their peak purity and quantity of fat content (Total Lipid Extract >5 μg g^-1^) were further characterised with Gas Chromatography-Mass Spectrometry (GC-MS) and Gas Chromatography-Isotope Ratio Mass Spectrometry (GC-C-IRMS). The compound-specific isotopic analyses of fatty acid methyl esters (FAMEs) provided complementary information on the nature of the animal fats contained in the pottery. The δ^13^C values of C_16:0_ and C_18:0_ fatty acids from modern animals raised on controlled C_3_ diets show that a distinction can be made between adipose fats of ruminant (cattle, sheep, and goats) and those of non-ruminant animals (domestic and wild pigs, horses and other non-ruminant wild species) due to differences in their metabolism and physiologies [65,66]. Discrimination can also be made between ruminant adipose and dairy fats via the same δ^13^C values. Therefore, according to modern lipid reference values, the Δ^13^C proxy (δ^13^C_18:0_-δ^13^C_16:0_) [66] was used to discriminate between non-ruminant adipose (>0‰/-0.3‰), ruminant adipose (<0‰/-0.3‰>−3.1‰), and ruminant dairy (<−3.1‰) products. These thresholds were adopted on the basis of the isotopic values of modern authentic reference fats from wild and domestic animals in European countries such as the United Kingdom, Germany, Switzerland, Spain, Italy, and Malta [67–73]. Differentiation of the type of animal fats when Δ^13^C values were placed between 0‰ and -3.1‰ also included the δ^13^C_18:0_ and δ^13^C_16:0_ isotopic ratios.

As our research was focused specifically on understanding the uses of different pottery sizes and shapes, the maximum diameter and height of the vessels were reliably measured in 96 cases (77%) (Table 2). Furthermore, 88% of the studied vessels (109 out of 124, Table 2) retained sufficient features to be assigned to a typological category.

**Table 2:** Number of vessels for which residues, type and/or size were successfully recorded.

Period	Number of vessels	N of residue results	N of classified vessels	N of vessels with height and/or max. diameter measured
**Early Neolithic**	7	7	3	1
**Middle Neolithic**	38	34	34	33
**Late Neolithic-CWC**	28	28	28	25
**Late Neolithic-BBC**	10	10	10	10
**Early Bronze Age**	36	25	30	27
**Later periods/Reference**	5	5	2	2
**Total**	124	109	109	96

In order to determine the degree of specialisation of pottery types, an index of dominance (Berger-Parker) and one of diversity (Margalef’s richness) were calculated using the results of the organic residue analyses for all types with more than five vessels analysed [74,75] (S1 File). A common definition of specialisation in functional analysis is that a particular tool type was only used to accomplish one task [76] while multipurpose tools were used for a range of purposes. Using lipid residues as a proxy for culinary tasks, the degree of specialisation of a given vessel type was assumed to be proportional to the dominance index and inversely proportional to the diversity of residues. To approximate the range of potential uses of pottery that would not yield a lipid residue, samples with less than 5 micrograms of lipid residue per gram under Acidified Methanol extractions were included in the calculations of the dominance and diversity indices.

## Results

Interpretable amounts of lipids (>5 μg g^-1^) [3,32], were detected in 33 of the 74 vessels (45%) extracted with DCM/MeOH and 79 of the 95 treated with the Acidified Methanol procedure (83%). Given that some sherds were prepared with both techniques, a total of 109 pottery vessels out of 124 (88%) were found to have contained lipid substances in the past (Table 1, S1 Fig and S1 Table). Except for seven vessels from the sites of Helmsdorf, Leubingen, and Bernburg, most samples contained only trace amounts of phthalate plasticisers which did not hinder the chromatographic analysis or interpretation of the results (S1 Supporting Information, section 3.4). Extensively contaminated samples (HE-1, HE-2 and HE-3 from Helmsdorf and LE-5 and LE-6 from Leubingen), possibly originating from older restoration works, were removed from the study.

**Fig 3:**
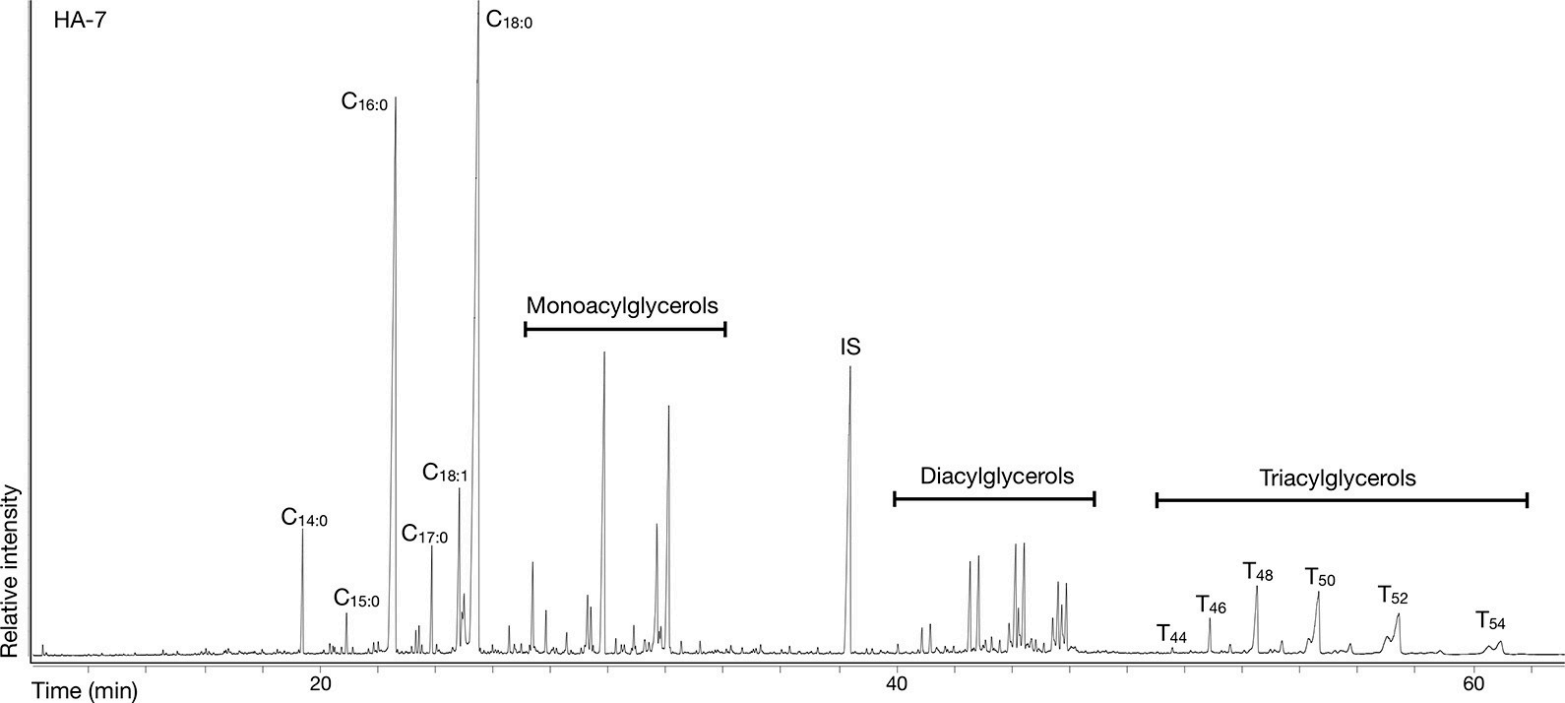
Example of a GC chromatogram of a solvent extraction from a vessel from Harsleben.

**Fig 4:**
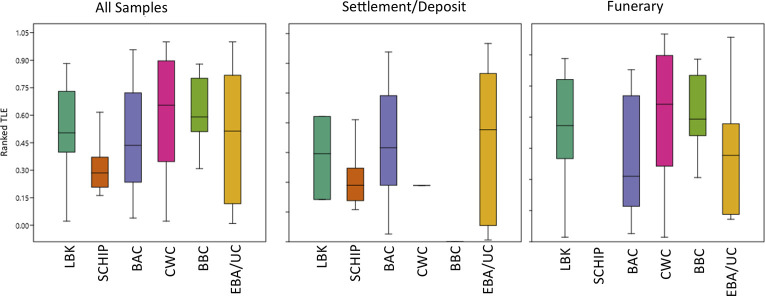
Variation in TLE across periods and settlement/deposit/funerary contexts. To integrate results from DCM/MeOH and Acidified Methanol extractions, TLEs for each extraction type were ordered by rank and normalised, S1 File), which placed them in a scale (y axis) from 0 (lowest lipid amount detected) to 1 (highest lipid amount detected).

DCM/MeOH extractions (Fig 3) presented a median Total Lipid Extract (TLE) of 13 μg g^-1^ with a standard deviation of 191 μg g^-1^ while the Acidified Methanol extractions produced a median TLE of 127 μg g^-1^ with a standard deviation of 1492 μg g^-1^ (Fig 4). No significant variation was observed in the fat concentration and recovery rates from the top, middle, or bottom parts of the vessels, whether in DCM/MeOH or Acidified Methanol extracts (S1 File). Thus, prioritisation of one vessel part to maximise lipid yields during sampling may not have changed the results, as lipids appear to be ubiquitous across the vessel profile. In 16 cases, a lipid concentration higher than 1 mg g^-1^ suggests that some vessels were intensively used for, and/or in contact with, high fat-yielding products. While such cases are found sporadically in most periods (e.g.: HA-13 MN 3.4 mg g^-1^; PO-17 BBC 1.2 mg g^-1^; KL-7 CWC 3.6 mg g^-1^), 62% of them were Corded Ware vessels (OE-6, PR-5, PR-9, PR-10, PR-14, PR-16, PR-17, PR18, WE-2, WE-3), ranging from 1.2 to 9.4 mg g^-1^ of recovered lipids. Conversely, while most sites exhibited preservation rates of 100% or close to it, the multi-period site at the ring sanctuary of Pömmelte [57,77,78] produced lipid extracts above 5 μg g^-1^ in only 57% of cases (16 out of 28 samples).

In 20 of the 33 (60%) positive samples extracted by DCM/MeOH, traces of triacylglycerols (TAGs, T44 to T54), diacylglycerols (DAGs), and monoacylglycerols (MAGs) indicated that fats were not fully hydrolysed during vessel use and burial (Fig 3). No evidence of wax esters was detected in the study. In the remaining samples, the lipid profiles were characterised by a range of even over odd chained free fatty acids consistent with full triacylglycerol hydrolysis. Most extracts contained compounds resulting from oxidation reactions, i.e., dicarboxylic acids, keto acids and cholesta-3,5-dien-7-one [8,79,80].

After correction for extraction-specific lipid recovery rates (see S1 File for details about this procedure, section 3.3), differences in the amount of lipids found in the 96 Acidified Methanol and the 74 DCM/MeOH extracts suggest varying intensities in the amount of recovered fats across different periods (Fig 4). Late Neolithic pots from Corded Ware and Bell Beaker burials show higher lipid values than funerary vessels from other periods except for the LBK (Table 3 and Fig 4) and 69% of the vessels had very high lipid quantities (>1 mg g^-1^). Furthermore, the Early Bronze Age TLEs present the highest standard deviations, potentially reflecting the samples’ origin from both funerary and settlement contexts, and/or a larger variety of uses compared to earlier periods. Given the number of factors potentially affecting the recovery of lipids in pottery (time since burial, fabric, porosity, vessel form, type of fat, vessel treatment during fieldwork and laboratory extractions), archaeological interpretations of these values must be treated with extreme caution as most of these factors could not be controlled for in this study. Strong variations between pottery types and periods such as the ones described here must, however, also consider interactions between fatty-rich substances and pottery vessels as one of the potential explanations.

**Table 3:** Pairwise Mann-Whitney tests for equal TLE medians between different prehistoric periods.

Mann-Whitney p value, TLE	EN/LBK	MN/SCHIP	MN/BAC	LN/CWC	LN/BBC
MN/SCHIP	0.14	-	**-**	**-**	**-**
MN/BAC	0.32	0.47	-	**-**	**-**
LN/CWC	0.37	<0.01	<0.0002	-	**-**
LN/BBC	0.20	<0.02	<0.005	0.5083	-
EBA/ Únětice	0.50	0.96	0.56	0.001	<0.05

Tests for the total lipid extracts in Acidified Methanol extractions between different prehistoric periods. The p-values which are lower than 0.05 (marked in red) indicate significant differences in the distribution of lipids amounts between two periods.

### Evidence of plant residues

Unlike most fats from domestic animals, plants tend to contain lower amounts and more easily degradable oils, which limits their preservation potential over time [81,82]. Consequently, tentative evidence for the processing of plant products was only detected in five samples across the study.

An LBK sample from Harsleben (HA-2) and a CWC sample from Oechlitz (OE-7) displayed unusually high palmitic to stearic ratios (3.5 and 3.1 respectively), which could be coherent with the low amounts of stearic acid detected in plant triacylglycerols [18]. Nonetheless, the absence of any additional biomarkers prevents the secure identification of plant residues in these samples.

**Fig 5:**
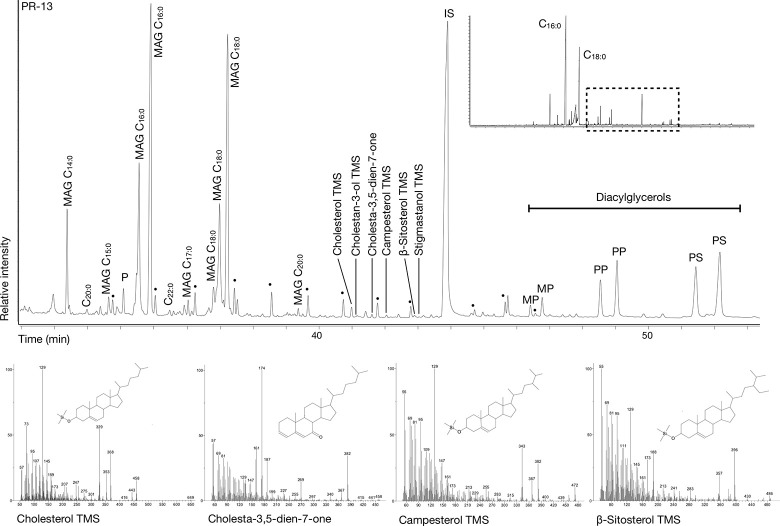
Chromatogram of sample PR-13 showing the presence of several plant and animal sterols (cholesterol, cholesta-3,5-dien-7-one, campesterol, and β-sitosterol) and their respective mass spectra.

Alternatively, a CWC vessel from Profen (PR-13) contained campesterol, β-sitosterol, and stigmastanol, compounds mainly produced by plant organisms, in a residue dominated by an equal amount of palmitic and stearic acid and trace amounts of cholesterol, cholestane-3-ol, and cholesta-3,5-dien-7-one (Fig 5). Although caution must be taken when interpreting sterols as they can be modern contaminants [80,83], the presence of several degradation products supports its archaeological origin. Given the clear presence of animal and plant biomarkers, a mixed use for both products is assumed in the case of sample PR-13.

**Fig 6:**
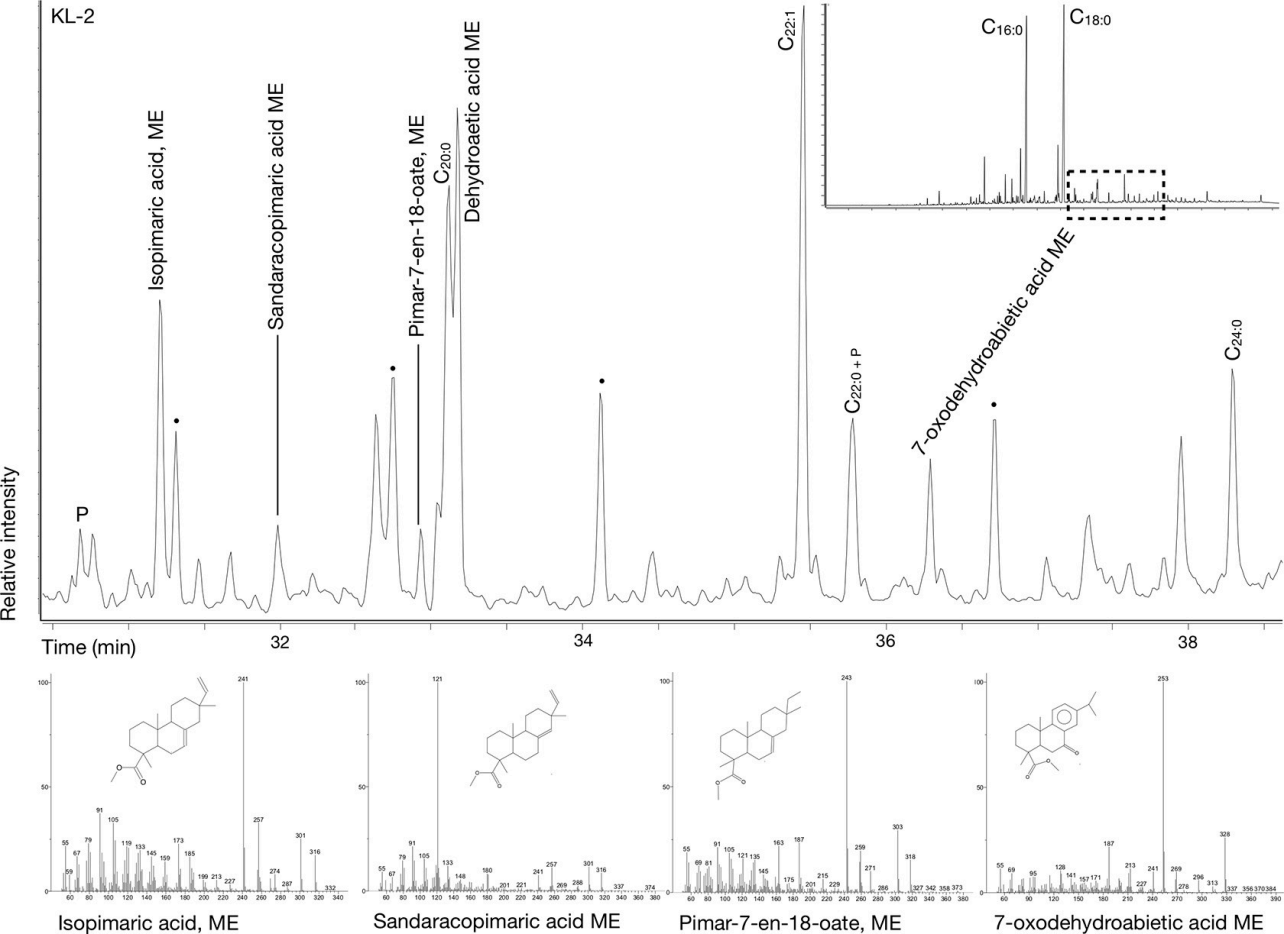
Chromatogram from sample KL-2 presenting a range of diterpenoids (isopimaric acid, sandaracopimaric acid, pimar-7-en-18-oate, dehydroabietic acid, and 7-oxodehydroabietic acid) diagnostic of conifer resins.

Several diterpenoids including dehydroabietic acid methylester (DHA), dehydrodehydroabietic acid (2DHA), 7-oxodehydroabietic acid (7ODA), 7-oxo-dehydrodehydroabietic acid (7ODDA), and a pimarane diterpenoid (PA) were detected in 17 samples from the sites of Queis, Pömmelte, Wennungen, and Kleinpaschleben. Nonetheless, given that diterpenoids can originate from multiple sources other than pottery use [84], only one Únětice vessel from Kleinpaschleben (KL-2) combining the presence of DHA, 7ODA and several PA (isopimaric acid, sandaracopimaric acid and pimar-7-en-18-oate) (Fig 6) was considered to comply with the criteria [85] for detecting degraded conifer resins in pottery.

### Evidence of thermal alterations

**Fig 7:**
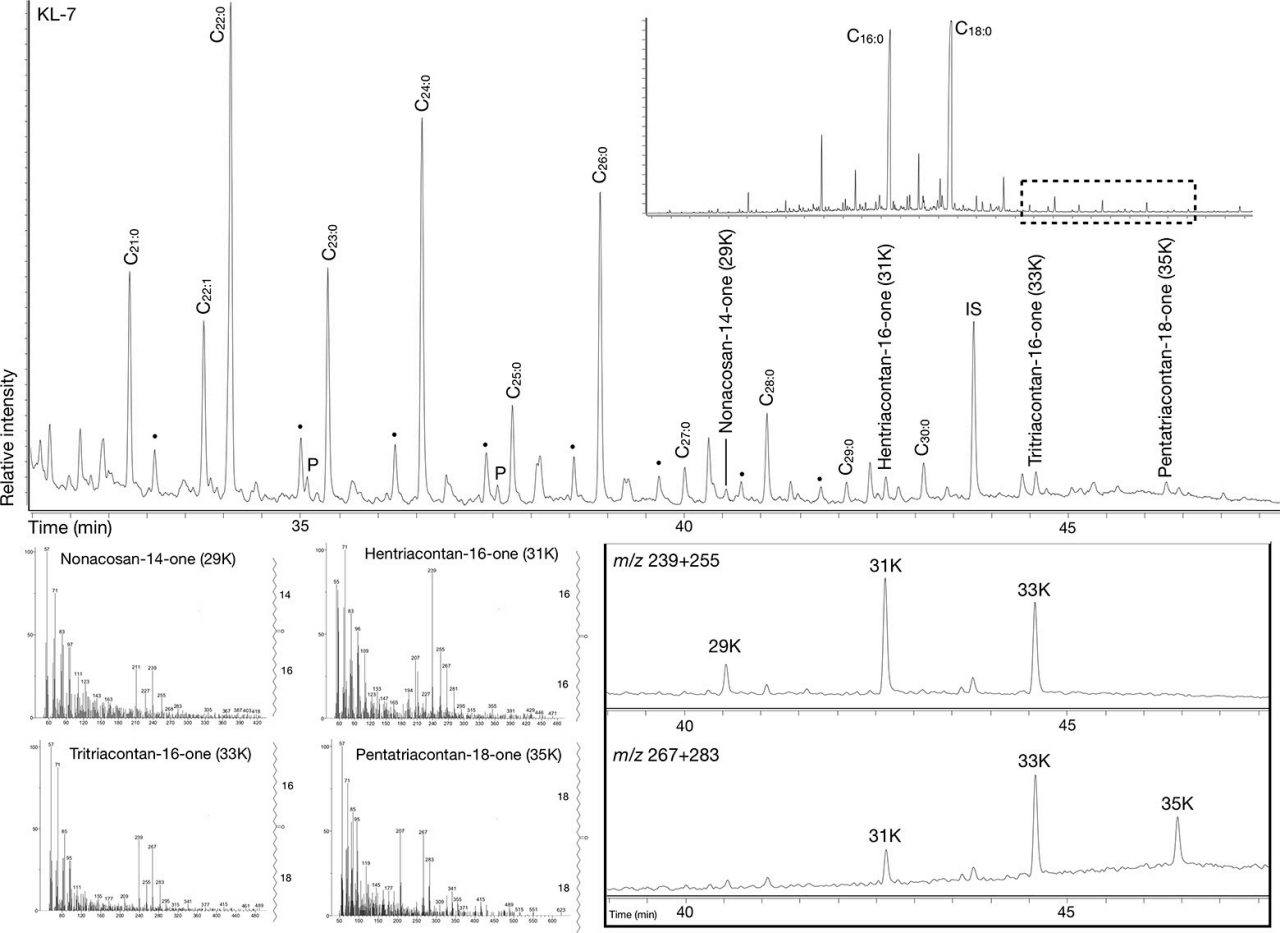
Chromatogram from sample KL-7 showing the presence of long chained ketones including nonacosan-14-one, hentriacontane-16-one, tritriacontane-16-one, and pentatriacontane-18-one.

**Fig 8:**
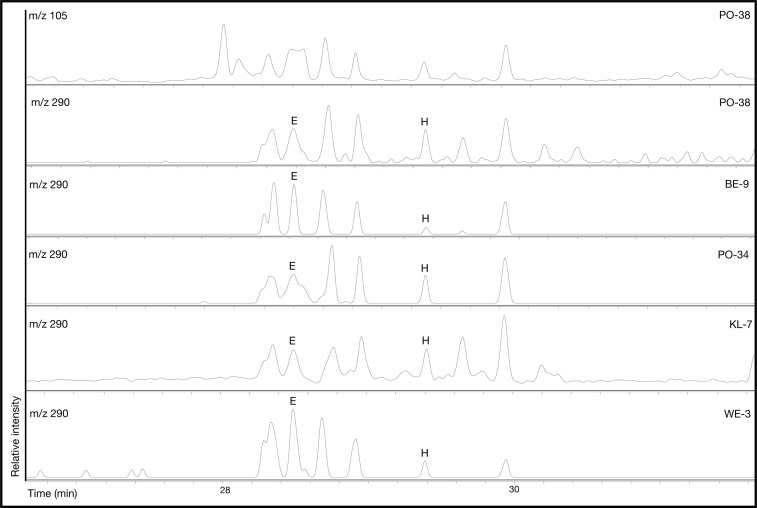
Partial chromatograms of *m/z* 105 and 290 from six samples showing the presence of several isomers of ω-(o-alkylphenyl)alkanoic acid of 18 carbon atoms.

Thermal alterations of the preserved lipids were attested mainly through the detection of characteristic long chained ketones (hentriacontane-16-one, 31K, tritriacontane-16-one, 33K, and pentatriacontan-18-one, 35K) [13,14] and ω-(o-alkylphenyl)alkanoic acids with 18 carbon atoms (APAA-C_18_) [15,86] in six samples (Figs 7 and 8). Following Bondetti et al. [16], the relative abundances of the E and H isomers were calculated to approximate the origin of the polyunsaturated fats in the residue. Additionally, other heating biomarkers such as ω-(2-alkylcyclopentyl)alkanoic acids (ACPAA) and very long chained oxo fatty acids (oxoVLCFA) [33] were also detected in some cases.

While most of the samples presented an E/H isomer ratio coherent with the values obtained from the alkali isomerisation of polyunsaturated fats from terrestrial animals (WE-3, PO-38, and PO-34), the high E/H ratio in BE-9 (8.8) has only been experimentally detected in cereals/fruits/non-leafy vegetables [16], which suggests that this residue could be the result of mixing and heating plant and animal products (Table 4). Additionally, samples PO-38 (1.7) and KL-7 (1.6) present an E/H value only reported from plants high in α-C_18:3_ or resulting from high thermal impact [16]. In these samples, the co-occurrence of long mid-chained ketones suggests that a high thermal impact could explain these E/H values, however, they could also result from plant oil high in α-C_18:3_ such as those in the *Linum*, *Brassica* or *Allium* species [87–89].

**Table 4:** Heating biomarkers detected in samples from this study.

Sample	Group	Period	LCK	APAA-C_18_	E/H	ACPAA	oxoVLCFA
BE-9	BAC	MN	No	Yes	8.8	No	No
WE-3	CWC	LN	No	Yes	5.9	No	No
PO-38	BBC	LN	Yes	Yes	1.7	Yes	Yes
PO-34	BBC	LN	Yes	Yes	2.5	Yes	No
PO-18	BBC	LN	Yes	No	-	No	No
KL-7	UC	EBA	Yes	Yes	1.6	Yes	No

### Evidence of animal residues

All samples except for the aforementioned HA-2 and OE-7 were dominated by a wide distribution of free fatty acids of various carbon chain lengths from C_12:0_ to C_28:0_. Hexadecanoic and octadecanoic acids, the most abundant compounds, were detected in similar abundance, which, combined with the presence of cholesterol, cholestane-3-ol, and cholesta-3,5-dien-7-one in some samples, strongly suggested that the recovered residues originated from the full hydrolysis of animal triacylglycerols.

**Fig 9:**
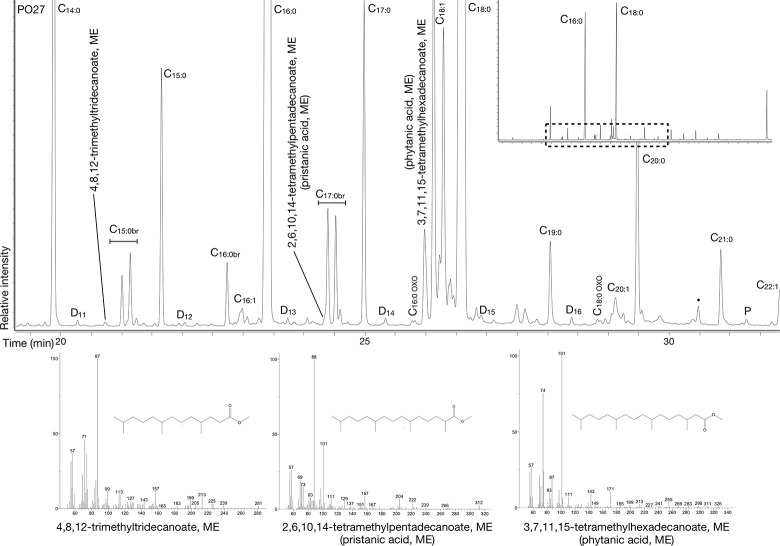
Chromatogram from sample PO-27 showing the presence of isoprenoic fatty acids: phytanic acid, pristanic acid, and 4,8,12-trimethyltridecanoate.

Isoprenoic acids resulting from the oxidation and hydrogenation of phytol, a constituent of chlorophyll, were detected in several samples. In one Únětice vessel from Pömmelte (PO-27, Fig 9), the combined presence of 3,7,11,15-tetramethylhexadecanoic acid (phytanic acid), 2,6,10,14-tetramethylpentadecanoic acid (pristanic acid), and 4,8,12-trimethyltridecanoic acid (4,8,12-TMTD) could be indicative of an input from aquatic resources. However, the widespread absence of aquatic resource biomarkers in the studied assemblage and the relatively low amount of 4,8,12-TMTD compared to phytanic acid [90] indicates that the recovered isoprenoic acids could also be of terrestrial origin.

**Fig 10:**
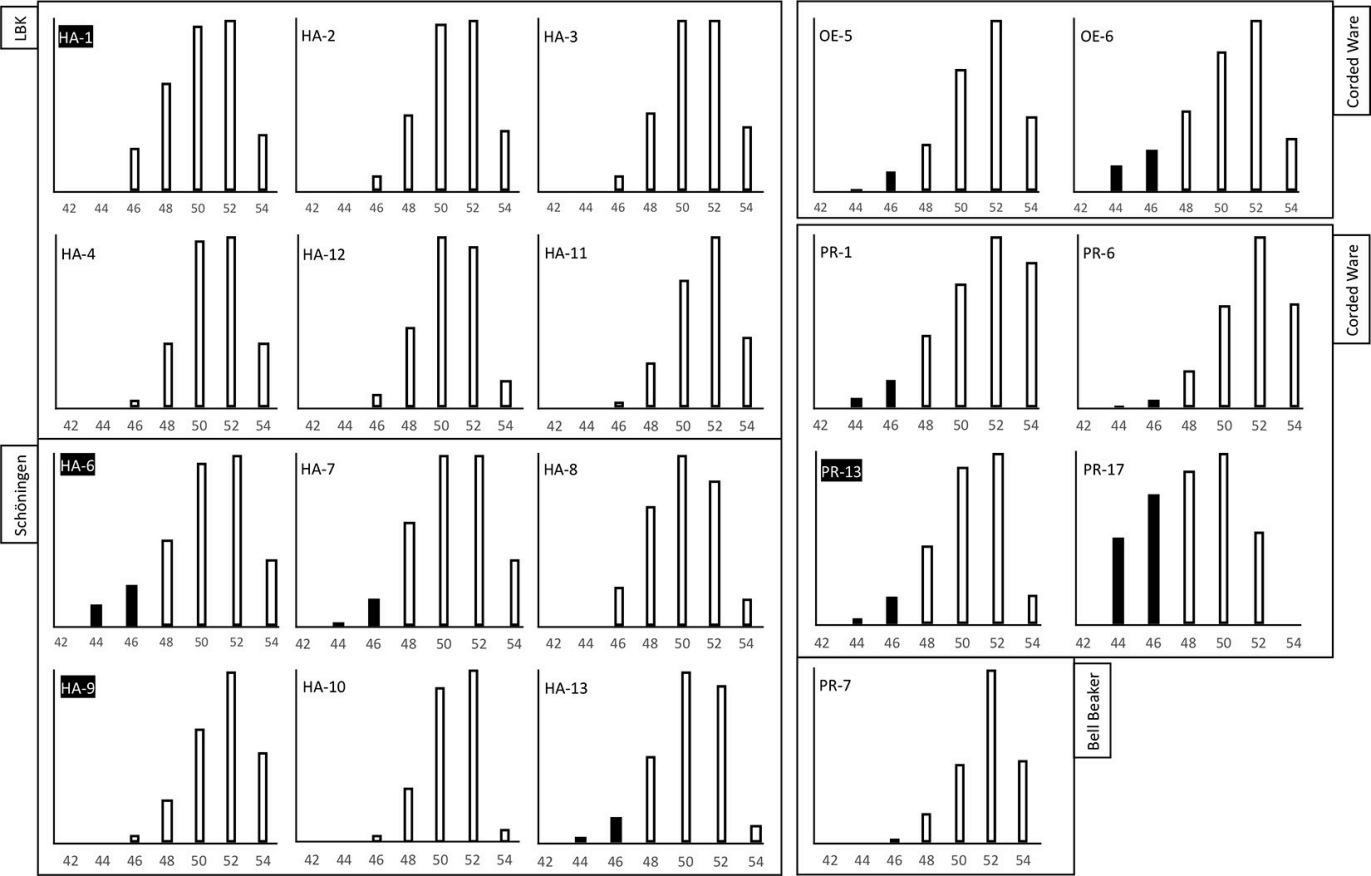
Triacylglycerol profiles from vessels from the sites of Harsleben (HA), Profen (PR), and Oechlitz (OE). Black bars mark triacylglycerols compatible with dairy products. Samples highlighted in black present isotopic signals coherent with dairy products.

A finer characterisation of the recovered animal fats initially explored the triacylglycerol (TAG) profiles of 13 samples from Harsleben (LBK and SCHIP), five samples from Profen (CWC and BBC) and two samples from Oechlitz (CWC) (Fig 10). The TAG molecular numbers, established through High-Temperature GC-FID known retention times, oscillated between 44 and 52 carbons. In eight cases (HA-6, HA-13, PR-1, PR-6, PR-13, PR-17, OE-5, and OE-6) a wide TAG distribution including T44 to T54 was considered suggestive of a ruminant adipose source or possibly a dairy source [2,91,92]. In the remaining 13 vessels, narrower distributions, T46–T54, did not preclude the presence of non-ruminant adipose fats. To improve upon this initial classification, the compound-specific δ^13^C‰ isotopic values of palmitic and stearic acids were measured in 106 of the total 109 recovered residues. The potential presence of mixtures of different fat types is best captured by combining isotopic data and TAG profiles.

**Fig 11:**
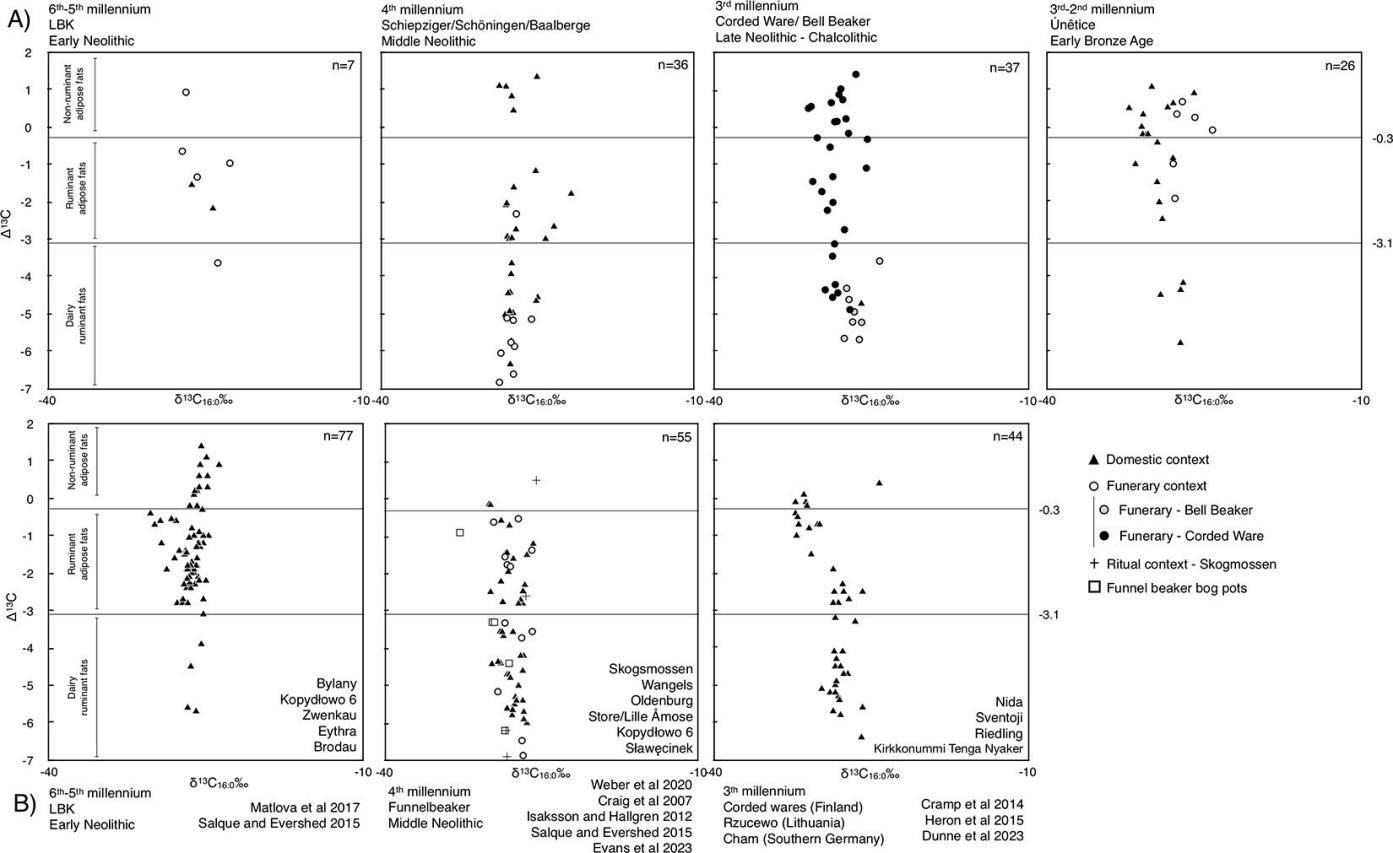
Isotopic values of A) new samples from this study and B) published results from the closest possible contemporary sites.

Overall, compound-specific δ^13^C‰ isotopic values oscillated between a maximum of -21.8‰ and a minimum of -32.2‰ with a median of -27.6‰ for palmitic acid (δ^13^C_C16:0_) and from -23.5‰ to -35.5‰ and a median of -30.5‰ for stearic acid (δ^13^C_C18:0_), which is coherent with lipid isotopic values reported from previous analyses on Neolithic pottery from central and northern Europe [6,20,93–98]. Δ^13^C values varied between -6.9‰ and 1.4‰ with a median of -1.8‰, thus demonstrating the presence of dairy, ruminant, and non-ruminant products across all the studied periods albeit in different intensities (Table 5 and Fig 11). None of the studied samples presented values coherent with either C_4_ or marine based products. Amongst non-ruminant fats, some samples showed isotopic values identical to domestic pig reference values (δ^13^C_16:0_: median -25.9‰, standard deviation 1.69‰. δ^13^C_18:0_: median 24.6‰, standard deviation 1.8‰. Δ^13^C: median 1.24‰, standard deviation 0.82‰) [68,70,71], while others presented more negative results (δ^13^C values lower than -27‰), which could be coherent with a wider range of species including wild (e.g.: boar, bear, badger, marmot, and hare) and domestic (e.g.: horse) animals and most plant oils or mixtures of those [17,71,72,81,99–103]. Although it has not been analytically possible to separate horse fats from the non-ruminant group [102,104], it is important to note that the high amounts of non-ruminant fats detected in vessels from the latter periods in this study would not be incompatible with the preparation of meals using this species.

**Table 5:** Summary of the types of animal fat recovered according to period, archaeological culture, and context of the analysed pottery.

Period	Culture/Group	N Samples		Dairy		Ruminant		Non-ruminant	
		Total	Funerary/Settlement/Deposit (No. of samples)	No.	%	No.	%	No.	%
Early Neolithic	Linear Pottery (LBK)	7	Funerary (5)	1	14	3	71	1	14
			Settlement (2)	-		2		-	
Middle Neolithic	Schiepzig/ Schöningen (SCHIP)	12	Funerary (0)	-	16	-	42	-	42
			Settlement (12)	2		5		5	
	Baalberge (BAC)	20	Funerary (9)	8	75	1	25	-	0
			Settlement (11)	7		4		-	
Late Neolithic	Corded Ware (CWC)	28	Funerary (25)	5	21	10	39.5	10	39.5
			Deposit (1)	1		-		-	
	Bell Beaker (BBC)	9	Funerary (9)	8	89	1	11	-	0
			Settlement (0)	-		-		-	
Early Bronze Age	Únětice (UC)	24	Funerary (5)	-	16	1	42	4	42
			Settlement (15)	1		8		6	
			Deposit (4)	3		1		-	
Late Bronze Age	Saalemündung (Ha B1)	2	Funerary (2)	-	NC	0	NC	2	NC

NC = not calculated. This table does not include samples used as references.

## Discussion

Applying a long-term and holistic perspective to lipid residues from different pottery forms not only provides insights into a key subsistence resource, fat, but also into the social practices implied in the preparation, storage, and consumption of fat bearing products (Table 5, Fig 11). Moreover, the size, shape, and decoration of the vessels may inform about the importance and social meaning of each source of fats, animal, or plant. From the first Neolithic settlers of Central Germany until the Bronze Age, pottery types allowed different communities, some of which were contemporary (e.g., CWC & BBC), to manifest their habits and values in distinguishable ways, beyond archaeological typologies. Thus, the study of multifunctionality, as opposed to the specialised use of pottery, offers a means to establish, in each context, whether pottery forms expressed specific culinary functions or whether they were related to social values, thereby crossing the material-ideal divide that has so preoccupied recent debates in archaeology and the social sciences in general [e.g., 105].

### Long term changes in pottery use and lipid sources

**Fig 12:**
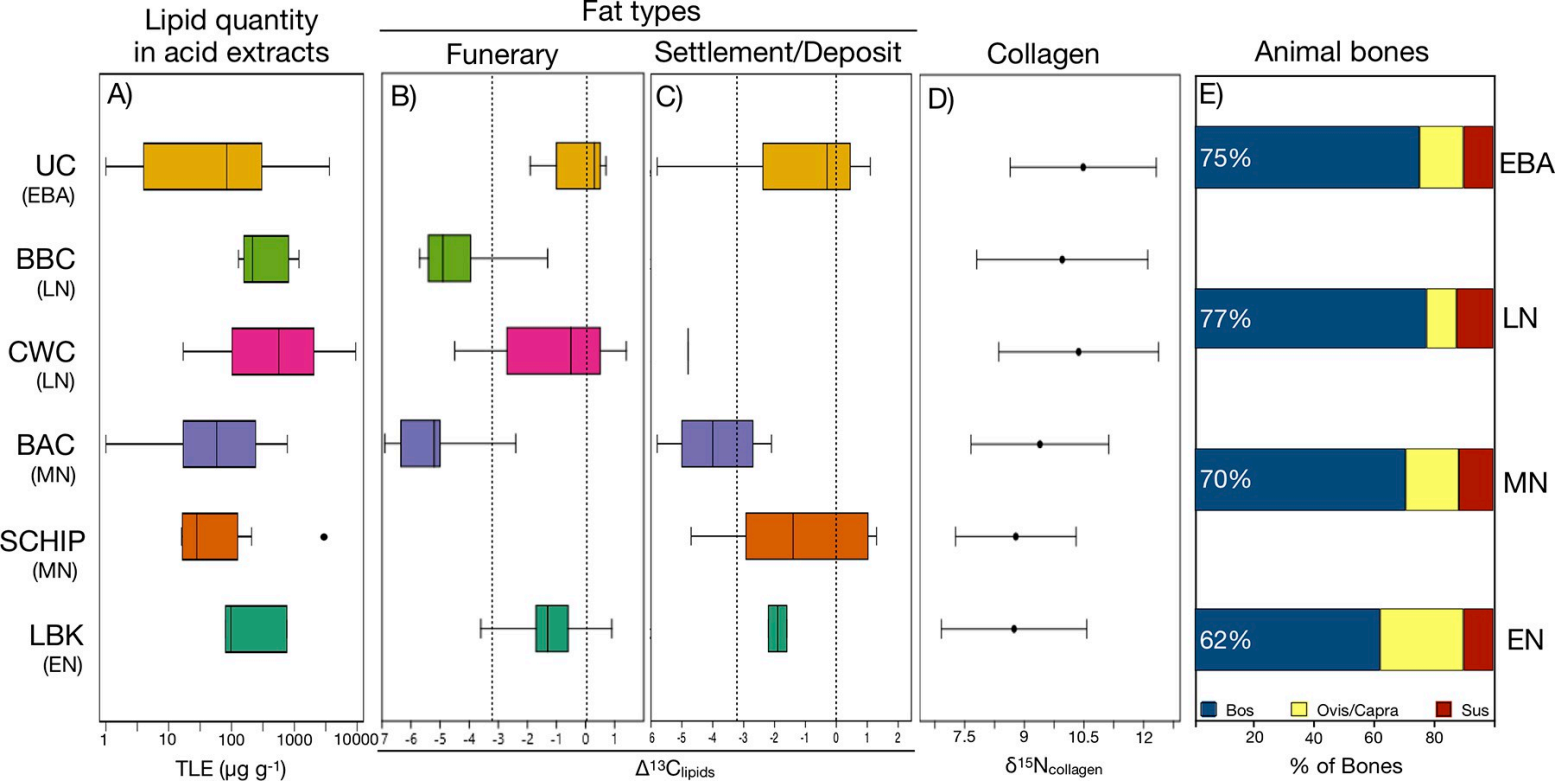
Changes in the amount and composition of animal fats preserved in pottery vessels across prehistoric periods. A) Box-plots of the total lipid extracts recovered through Acidified Methanol extractions. B and C) Box-plots presenting the Δ^13^C proxy for animal fat types for funerary and settlement/deposit contexts. D) Mean-and-whisker (95% interval) plot presenting published δ^15^N bone collagen isotopic values from contemporary burials [109]. E) Composition of contemporary herds based on bone frequency. Note that animal bone data from the LN period originates only from the site of Bottendorf [27,106–108].

As initially stated, pottery mediates between subsistence production and consumption, the two complementary and indispensable practices which allow socio-economic reproduction. Our findings do not provide substantial evidence for a significant inclusion of aquatic and/or plant-derived lipid products in the pottery culinary practices of Central Europe, albeit these being more easily degradable, while results show that animal derived lipid products were clearly and, in some cases, intensively stored, prepared, and served in pottery vessels. The abundant faunal record available for Central Germany, with over 59,000 classified faunal remains from 38 archaeological sites, provides a detailed insight into the human-animal relations from the Early Neolithic to the Bronze Age [27,106–108]. These show that hunted animals, mainly red deer and wild boar, represented always less than 10% of the identified bones and played only a marginal economic role. During the Early Bronze Age its importance dropped to around one percent. It is unlikely that they significantly contributed to the detected ruminant and non-ruminants fats. Apart from meat, the ruminants–cattle, sheep, and goat–could have been potential providers of milk and milk derived products. Interestingly, according to the available faunal record, the relative amounts of these animals were not significantly modified in the Middle Neolithic (Fig 12E), the period when a more intensive dairy processing was observed in pottery (Table 5, dairying in Baalberge at 75%, and Fig 12B and 12C). Age at death profiles from the nearby 4^th^ millennium sites of Salzmünde, Krautheim, and Groβobringen [106] suggest that the ovicaprines’ slaughter patterns fit a strategy to maximise the production of dairy products, but when other sites are taken into account, significant variability suggests that different meat centred, dairy centred, or wool centred strategies were practiced in this period. Pigs, potentially the main origin of non-ruminant fats in pottery, had practically the same importance throughout later prehistory (Fig 12E). In sum, the apparently constant husbandry practices suggested by the faunal record do not correlate with the important variations in lipid composition found in the analysed pottery assemblage (Table 5). The broad changes in subsistence economy reflected in pottery lipid residues could have been caused by different animal management strategies, local differences in the production of their derived products, and the development of different culinary practices or tastes. Said changes, potentially leading to diverse ways of consuming food, could be expected to affect the size and shape of kitchenware and tableware. See, for example, the case of the Corded Ware amphorae, significantly skewed towards non-ruminant fats (Table 6).

**Table 6:** Use of the pottery forms, and their functional dominance and diversity indices (I.dom., I.div.).

GROUP (Period)	TYPE	ArchID	N	No	AD	AR	AN	P	I. dom.	I. div.
**SCHIP (MN)**	Pots	HA-9, 10, 13, 14, LI-1, 2, 3	7	0	1	3	3	0	0.43	1.03
	Biconical amphorae	LI-4, 5	2	0	0	0	2	0	-	-
**BAC** **(MN)**	Cups	BE-2, 4, 11, QU-42, 44, 46, 49, PO-15, 19	9	0	8	1	0	0	0.89	0.46
	Small bottle-shaped amphorae	QU-41, 43, 47, 48, PO-13, 14, 20	4(+3 A*)	3	1	0	0	0	-	-
	Large bottle-shaped amphora	QU-45	1	0	0	1	0	0	-	-
**CWC** **(LN)**	Beakers	PR-3, 10, 17, BR-1, WE-3, OE-4, 7	7	0	3	3	1	0	0.43	1.03
	Cups	PR-1, 2, 6, 13**, OE-1, WE-2, PO-32	8	0	2	4	1	1	0.50	1.44
	Amphorae	PR-4, 9, 11, 14–15, 16, 18, WE-1, OE-2	8	0	0	1	7	0	0.88	0.48
	Bottle-shaped amphora	OE 5–6	1	0	0	1	0	0	-	-
**BBC** **(LN)**	Beakers	PO-17, 18, 21, 35, 36, 37, PR-7	7	0	6	1	0	0	0.86	0.51
	Footed bowl	PO-34	1	0	1	0	0	0	-	-
	Bag-shaped cup	PO-38	1	0	1	0	0	0	-	-
**UC** **(EBA)**	Classical cups	PO-22, 23, 24, 25, 26, 27, 28, 29, 30, 31, 39, ME-5, KL-2**	14	7	3	1	2	1	0.50	1.52
	Pithoi	PO-12, 33, KL-3, 10, OE-8	5	2	0	1	2	0	0.40	1.24
	Pots	KL-4, 7, 6, 11, ES-1, 2, OE-9	7	0	1	3	3	0	0.43	1.03
	Cups	PO-16	1	0	0	1	0	0	-	-

Indices are only calculated when five or more different lipid results are available (see S1 Fig and S1 Table). **No**: no fat identified; **AD**: animal, dairy fat; **AR**: animal, ruminant fat; **AN**: animal non-ruminant fat; **P**: plant-derived fat. Archaeological culture/group codes as in Table 1. Sample codes as in S1 Fig and S1 Table. * Three uncharacterised animal fats. **: Vessels containing two distinct types of residues and are counted twice in the N column.

Complementing the study of the zooarchaeological remains, stable isotope analyses carried out on human remains from EN to EBA burials have confirmed a steady increase in the consumption of animal protein, as indicated by δ^15^N values [109] (Fig 12D). At the same time, in our study, most of the vessels containing more than 1 mg g^-1^ of fats were detected in the LN and EBA periods. A trend of increasing lipid concentration in pottery (Fig 12A), for which biomarkers indicate an animal origin, is also observed in our data. However, despite animals generally exhibiting a higher fat content than plants and the limited plant evidence in this study, there is a certain degree of equifinality. Several factors such as the fabric, the porosity, the lifespan of the vessel, soil conditions, or vessel form may also affect the observed lipid concentrations.

Interestingly, rather than showing a long-term shift to dairy products, a possibility stated by Münster et al. [109], the Δ^13^C values (Fig 12B and 12C) in pottery residues show no direct correlation with δ^15^N values. As the first is an indicator of the origin of the fats prepared using pottery [66], this suggests that the increase of protein consumption across time does not seem to be the result of a gradually stronger dairy economy supplementing meat intake with protein-rich dairy products such as yoghurt or cheese. Meat consumption could have been substituted by dairy products, but this does not seem coherent with the slaughter profiles, optimised from meat production in sites contemporary with the high-dairy periods [106]. Alternatively, milk could have been consumed in the form of cream or butter, which would not affect collagen δ^15^N values as these products contain either significantly less protein or no protein at all [115].

**Fig 13:**
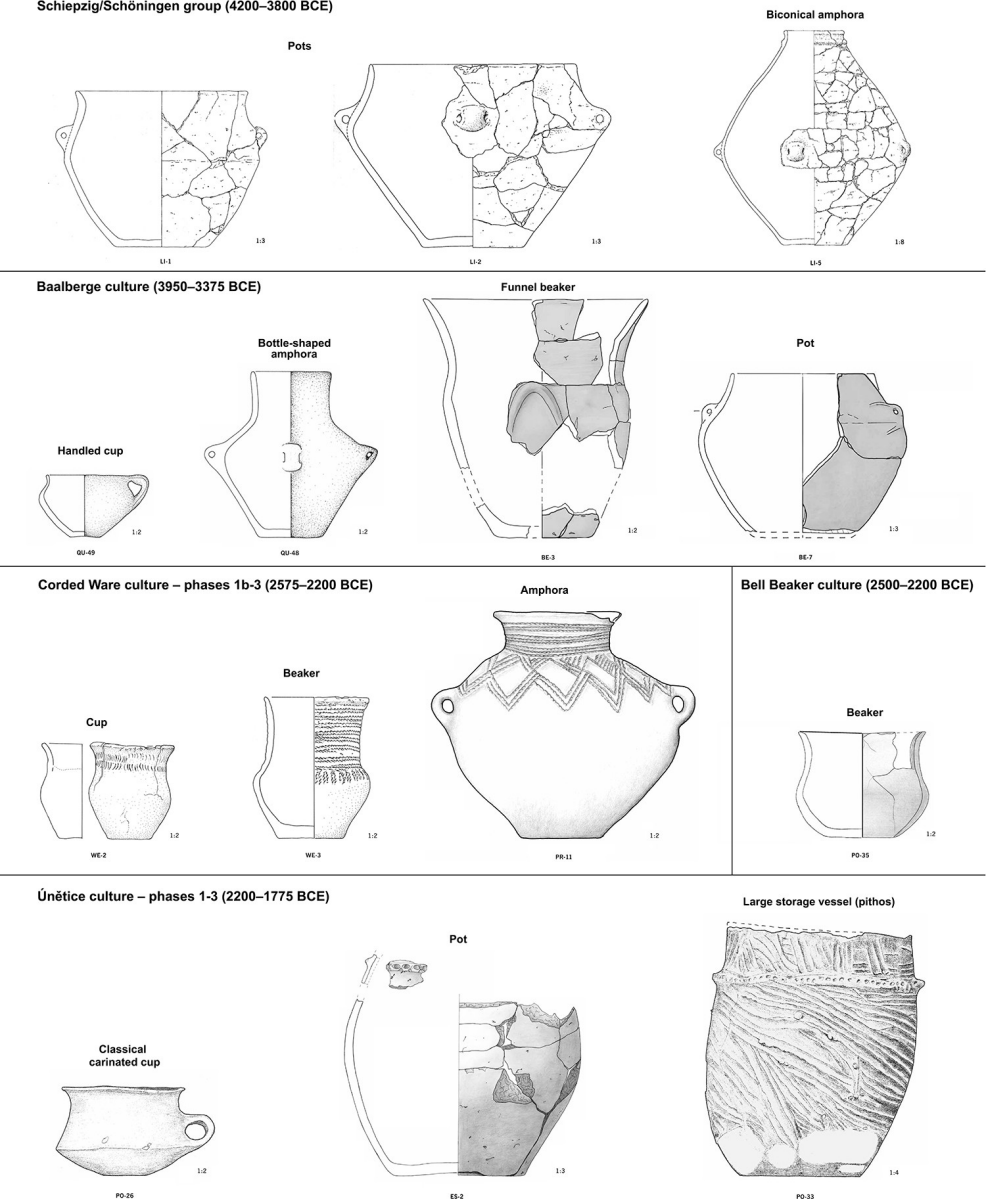
Main pottery types of Central Germany submitted to lipid analyses.

**Fig 14:**
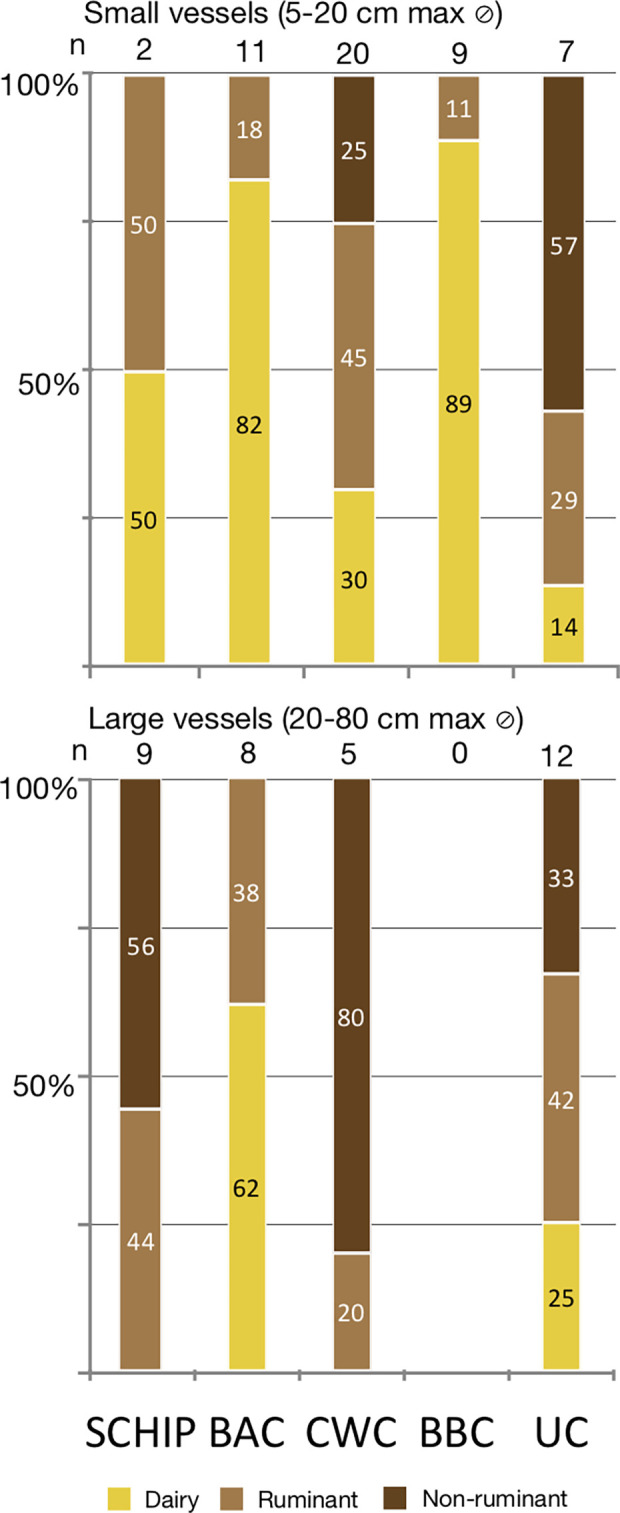
Percentages of dairy, ruminant, and non-ruminant adipose fats by vessel size over time, in settlement and funerary contexts. Vessels are classified according to their maximum diameter (Ø) (≤20 cm, or >20 cm).

Thermally altered bones showing evidence of roasting or drying meat directly on the fire, which are potential sources of adipose fats in dietary intake, have been documented at contemporary sites [107]. However, other activities such as tool confection, the use of bones as fuel or for hygiene, and burning accidents can affect bones in a similar way. While this study is unable to account for these sources of adipose fat, our results for pottery-based cooking indicate complex long-term changes in the preparation and consumption of animal products in Central Germany, particularly with regard to dairy and non-ruminant products. To elucidate how different groups in the region may have employed different strategies for cooking in pottery, the functional roles of different pottery forms (Fig 13) and sizes (Fig 14) in each period were assessed through their degree of specialisation (Table 6).

In total, the degree of specialisation when preparing or consuming fatty-rich substances of nine basic ceramic forms, combining shape and size, were defined by the indices of dominance and diversity (Table 6). These were forms from all chronological phases and groups, except LBK, with more than five studied cases. The diversity and dominance indices, widely applied in the study of archaeological remains (see S1 File), support diversification of use when low indexes of dominance (≈0.5) are accompanied by a high index of diversity (≈1). On the contrary, potentially specialised vessels used to transform a narrower range of products will result in high dominance indices (≈0.9) and a low index of diversity (≈0.5). Results identify two patterns of functional specialisation/diversification throughout time linked to food preparation. Next to a somewhat generalised non-standardised use of most forms, at least three show dominance indices close to 0.9 and diversity indices around 0.5, implying that they may have had a much more specific function when preparing or consuming fatty-rich products. These are Middle Neolithic Baalberge handled cups, where dairy products dominate, Late Neolithic Corded Ware amphorae, where non-ruminant fats dominate, and Late Neolithic Bell Beakers, where dairy products dominate. We find the case of the Baalberge handled cups particularly informative as, together with sieves used for the preparation of dairy products [110], they may constitute the first evidence for vessel forms specialised in the transformation or consumption of dairy products in Europe at a time when their consumption was becoming widespread [19].

### Insights from specific periods

#### LBK (Early Neolithic)

According to the obtained isotopic values for the C_16:0_ and C_18:0_ fatty acids, ruminant adipose fats dominated (88%) amongst the eight analysed vessels from the LBK layers in Harsleben. Only one case, a small decorated funerary cup (HA-1) found in a typical LBK burial of an adult male, presented isotopic values indicative of dairy ruminant fat and a narrow range of TAGs, which did not exclude the possibility of a mixture with other adipose lipids. In the same grave, this vessel was accompanied by two medium sized pots (HA-2 & 3) which contained ruminant fats (see S1 Table). The processing of dairy fats in Central Germany during the Early Neolithic had so far only been detected in a ceramic sieve from Brodau [4] while sherds of non-reported forms from the LBK sites of Zwenkau and Eythra, western Saxony, presented mixtures of ruminant and non-ruminant products [4 page 56]. Similarly, the LBK community of Harsleben, situated at the northern fringes of the LBK expansion (Fig 1), seems to have been more specialised in ruminant rather than non-ruminant food sources.

#### Schiepzig/Schöningen groups (Middle Neolithic)

Despite the limited typological information available for Early Neolithic vessels, impeding the functional analysis of LBK pottery, a substantial number of well-preserved small to medium sized pots of the Schiepzig/Schöningen groups were examined. Their results reveal a variety of purposes, though always involving cooking or storage of animal fats (dairy, ruminant, and non-ruminant) (Tables 5 and 6). This functional diversity correlates with a variety of slightly different body and rim shapes (Fig 13). Alternatively, the large biconical amphorae of the Schiepzig group in Libehna (Table 6, LI-4, LI-5) might have been used specifically for storing non-ruminant fat products (Fig 14), a result which suggests the existence of a trend that will require further confirmation by analysing a larger number of vessels.

Dairy products, present in two out of seven vessels (29%) in Harsleben’s MN Schiepzig/Schöningen settlement contexts, were only slightly more abundant than in the LBK period. The sample HA-6 (not included in Table 6 as shape information was not preserved) presented a dairy TAG distribution, but in HA-9, the only SCHIP pot with a dairy isotopic signal, TAGs did not rule out a mixture with other animal fats. In Libehna, none of the five analysed vessels presented dairy products. Instead, the majority (n = 4) were coherent with non-ruminant adipose fats (80%). Only one vessel (LI-3) presented a ruminant adipose signal. Furthermore, the presence of non-ruminant fats in two large pithoi of over 50 cm in height (LI-4 & 5), underlines the potential importance of non-ruminant products at least at this site. This could be coherent with the slight increase in domestic pig zooarchaeological remains detected within the Middle Neolithic (Fig 12E) [107].

#### Baalberge (Middle Neolithic)

In the 4^th^ millennium, results from the BAC (n = 19) present a completely different picture both in settlement and amongst funerary vessels. Dairy fats were detected in eight out of nine one-handled, small to medium sized cups (Table 6, Figs 13 and 14), (88%). An elegant medium-sized funnel beaker (BE-3), large vessels with small handles (BE-7 & 9), and a bowl (BE-5) may also have been used for producing, storing, and serving dairy products. Only the largest container (BE-8) and the sole large bottle-shaped amphora (QU-45) would not have been used for this purpose and were coherent with ruminant adipose fats (Fig 14). Considering that the typical grave inventory of the Baalberge Culture consists of a handled cup combined with a small amphora, it is significant that cups in funerary contexts bore residues of dairy products (BE-4, QU-42, PO-15, PO-19) while amphorae contained traces or no lipid residues at all (QU-43, PO-12, PO-13, PO-14). This potential trend warrants additional investigation in future studies.

So far, the two small- and medium-sized funnel beakers (BE-3, QU-40) contained dairy products, whereas the small and large funnel shaped vessels (BE-6, 8) exhibited ruminant residues. However, numbers are still too small to yield secure trends. In graves, the use of the typical small bottle-shaped amphorae, frequently combined with a drinking vessel, is still uncertain. While most of them contained undefined fats or no fats at all, at least one preserved dairy residues. Contrarily, the handled cups nearly always contained dairy lipids, indicating a highly specialised use related to milk-derived food sources. One hypothesis is that maybe these cups served to scoop milk products from the large conical rim vessels found in the settlements (BE-7, 9). If confirmed in the future by a larger number of samples, this would imply that the rising importance of dairy products in the BAC went hand in hand with a specific set of pottery types used to prepare and consume this novel food.

#### Corded Wares (Late Neolithic)

This increase in dairy consumption, however, would not seem to be sustained by the later CWC groups of the 3^rd^ millennium in Central Germany. Then, out of 31 cases, only six presented dairy isotopic values (19%), while non-ruminant (n = 11, 35%) and ruminant adipose fats (n = 14, 45%) dominated again. However, five additional samples from Profen and Oechlitz presenting isotopic values coherent with adipose fats (PR-1, PR-6, PR-17, OE-5, and OE-6) yielded TAG profiles suggesting potential mixtures with dairy products. With regards to funerary vessels, the variety of pottery types placed in certain burials appears to correlate with different uses. The young adult from grave 7618 in Profen was buried with a beaker (PR-12), a cup (PR-13), and an amphora (PR-11) containing dairy, ruminant, and non-ruminant fats, respectively. One beaker and two cups (PR-1, 2 and 3) placed in the grave of an adult individual (code 5043) contained ruminant and non-ruminant adipose fats, whereas the amphora (PR-4) of the same burial again contained a non-ruminant fat. In view of the clear differences in the placement and orientation of male and female bodies in CWC burials [e.g., 111], it is noteworthy that no gender specific norms existed with regards to the shape nor the contents of the pottery placed in graves.

The typical decorated beakers and smaller cups of the four different CWC sites analysed contained a diversity of animal and even plant derived fats, suggesting that, in this case, the standardisation of the shape and decoration of the pottery did not go hand in hand with a specialisation in their use. This apparent functional diversity could place the distinctive formal and decorative attributes of these beakers at a socio-political and ideological level, which would exceed the strictly economic realm. So far, our results do not support the interpretation of these cups as special vessels used for drinking cereal derived beverages, such as beer, as has been claimed in the past [112,113]. This situation is reversed in the case of amphorae, a standardised shape accompanied by a very narrow range of lipid residues identifiable as non-ruminant fats. Although their isotopic values are not incompatible with plant oils, the absence of other plant biomarkers in the vessels suggest that animal fats, most probably horse or boar, are more likely. It is noteworthy that the isotopic values of the fats in these vessels do not correspond with the reference values of any of the most common domestic animals in contemporary zooarchaeological assemblages (cattle, sheep, goat, and domestic pig).

#### Bell Beaker (Late Neolithic)

No lipid analyses from the early typical zone-decorated Bell Beakers were available before this study. The Bell Beaker (BBC) funerary vessels from Pömmelte reveal that dairy production may have been significant in the second half of the 3^rd^ millennium, as all of the six analysed undecorated carinated beakers, a decorated bag-shaped cup (*Schlauchkrug*), and a footed-bowl contained a clear signal of dairy products. Some of them (PO-38 and PO-18) also included evidence for protracted heating. The use of the carinated beakers, at least at the site of Pömmelte, seems to have been highly specialised in dairy products, potentially as a serving vessel, as already observed among similarly shaped BAC cups. Such a use, rather than being representative of the whole period, may reflect specific funerary practices at Pömmelte, where the typical grave good, a single drinking vessel, consistently presented a signal of dairy products across multiple burials. Only one similar beaker from a burial found in Profen (PR-7) contained ruminant adipose fat. Dairy fats were also identified in the only undecorated footed bowl (so called “Füßchenschale”, PO-34) and were accompanied by heating biomarkers, suggesting that the pottery type used as a grave good may have played only a subordinate role to the vessel contents.

#### Únětice (Early Bronze Age)

Finally, at the end of the 3^rd^ and start of the 2^nd^ millennium BCE, the emergence of the Únětice (UC) society seems to see a return to the consumption of mainly ruminant (n = 10, 42%) and non-ruminant (n = 10, 42%) adipose fats instead of dairy fats, which are found again in only a handful of vessels (n = 4, 16%). This diversity of uses, as compared to the BBC contexts, results from an assemblage mainly composed of settlement vessels (77%) as well as a few funerary vessels (33%). However, of the ten classical UC carinated cups of different sizes found in a settlement hoard in Pömmelte, three were related to milk derived products, one to ruminant fat (PO-30), and six contained no lipids at all. Large and medium sized containers, often showing the typical rough plastic decoration (so called Fingerstrich) and/or appliqués with finger tip impressions included dairy, ruminant, non-ruminant, or no fats at all, suggesting that the Únětice pottery types did not correlate with specific uses in any of the settlements analysed.

Pottery in the UC settlements thus does not seem to have been designed for highly standardised uses. The carinated cup with one handle, a classical form in this period, was found to contain different lipid residues or none, implying a high diversification and low functional standardisation (Table 6). Large and small storage vessels show a similar variety of residues (Fig 14). Thus, Únětice potters seem to have produced a limited range of multi-purpose forms instead of manufacturing specific types for exclusive functions. Technologically oriented studies of the UC pottery production are required to determine whether this change in productive strategy was a response to an increase in pottery functions, or to an increase in vessel manufacture, as it seems to have been the case in El Argar. In this EBA society from south-eastern Iberia, a limited number of shapes was used to accomplish all required functions [114].

## Conclusions

Overall, the study of the lipid residues in different vessel-types from the Early Neolithic to the Bronze Age in Central Germany reveals a complex picture resulting in the detection of broad changes in pottery use and food preparation over time, fuelled by diverse period-specific strategies where different vessel types presented different use patterns and degrees of specialisation.

In contrast to the 6^th^ and 5^th^ millennium BCE, LBK, and SCHIP pottery use patterns focused on the consumption of ruminant or non-ruminant adipose lipids, the 4^th^ millennium BCE evidence gathered hitherto shows a strong increase in the reliance of dairy products as the main source of dietary lipids from pottery (Table 4). This coincided with the appearance of at least one dairy-specialised form and the development of the BAC and the corresponding demographic shift brought by the Funnel Beaker and Michelsberg cultures. Furthermore, these results are in line with contemporary 4^th^ millennium BCE data from Skogsmossen, Wangels, Oldenburg, Store/Lille Åmose, Sławęcinek, and Kopydłowo 6 in Poland, Denmark, and Sweden [20,93,97,98,115] (Fig 11) amongst many sites [19], demonstrating a strong shift towards the use of dairy products in pottery-based culinary practices, constituting the strongest evidence for the first widespread adoption of dairying in northern Europe. The stronger reliance on dairy products by the BAC groups seems coherent with the development of new economic strategies managing animal herds to maximize milk production and of new sets of pottery types, such as cups with high carinations and handles, apparently developed to prepare and consume this product. Interestingly, these economic changes did not seem to affect the species composition of the domestic animals herds, which suggests that the social management of animals was most probably a driver of change which also led to an increase in the importance of animal protein in the diet, as suggested by δ^15^N isotopic values.

The arrival of the new populations associated with the CWC and BBC in the 3rd millennium resulted in a notable change of scenario, returning to a majority consumption of products bearing non-dairy fats in a context of an increasing pottery shape standardisation which does not always correlate with stronger functional vessel specialisations. While the typical decorated CWC amphorae has been tentatively identified as a special type of container for non-ruminant derived products, the standardised cups and beaker shapes may have served for a wider variety of culinary purposes. Intensive cases of dairy production and consumption were detected in CWC-related assemblages [95] and in one of Pömmelte’s beaker deposits. However, the vessels with the highest amounts of lipids across all the studied periods, located in CWC sites such as Profen, Wennungen, and Oechlitz, contained mainly non-ruminant fats. It has also been noted that the funerary assemblages considered show no association between specific products and the biological sex and age of the buried individuals. Thus males and females were often buried with a variety of forms, each containing a different product.

The Únětice wares of the end of the 3^rd^ and first half of the 2^nd^ millennium BCE show a departure away from the Final Neolithic period, as detected in certain contexts at the site of Pömmelte, by maintaining the prevalence of adipose fats over dairy products. The new pottery shapes were not accompanied by a clear functional specialisation of pottery types, as seen by high diversity indices and low dominance indices in all the studied forms. An increase in availability and consumption of animal products, seen in δ^15^N values, is complemented by the presence of animal adipose fats in a wide variety of containers and is coherent with the detection of specialised slaughtering installations in Salzmünde-Schiepzig and in Gotha-Sundhausen [43,109].

In conclusion, this first diachronic study on prehistoric pottery use from a concise region in Central Europe (the south of modern day Saxony-Anhalt) has shown how combining lipid organic residue analysis with more conventional contextual and pottery typology studies may reveal complex realities of changing culinary gestures and practices otherwise left unnoticed by other diet indicators. Thus the initial complex trends detected here merit the development of future studies including a larger number of samples from each period. The detected steady increase in protein consumption, seen through δ^15^N values over several millennia, implies a higher overall availability of animal fat-rich products, which is coherent with the detection of animal fats in settlement contexts, but contrasts with changes in the contents of funerary vessels, episodes of strong and weak dairy consumption in pottery, the development of specialised pottery types, and the formation of assemblages with highly formalised but multipurpose vessels. Over time, as an intricate history of pottery use unfolded, distinct groups developed different strategies incorporating pottery in the acquisition, transformation, and consumption of lipid-rich foodstuffs, responding to the needs and challenges of their respective economies, and gave new meanings to those fired clay containers invented by their south-eastern forbearers millennia ago.

## Supporting information

S1 Fig Vessel contexts, drawings, and results. (PDF)

S1 Table Table of lipid results and vessel sizes. (DOCX)

S1 File Analytical parameters and statistical treatments. (DOCX)
