# Supplementary material for: Pottery spilled the beans: Patterns in the processing and consumption of dietary lipids in Central Germany from the Early Neolithic to the Bronze Age
Source: PLoS One. 2024 May 16;19(5):e0301278. doi: 10.1371/journal.pone.0301278 (PMC11098342; doi:10.1371/journal.pone.0301278)
Supplement: S1 Fig — (PDF) [file pone.0301278.s001.pdf]

Linear Pottery (LBK 5450–4775 BCE) – Funerary and Settlement

Legend

- A animal fat
- AD dairy
- AR ruminant
- AN non-ruminant
- V plant-based
- – absence
- >100 °C
- >300 °C
- funerary
- settlement
- deposit
- **HA-1** sample no.

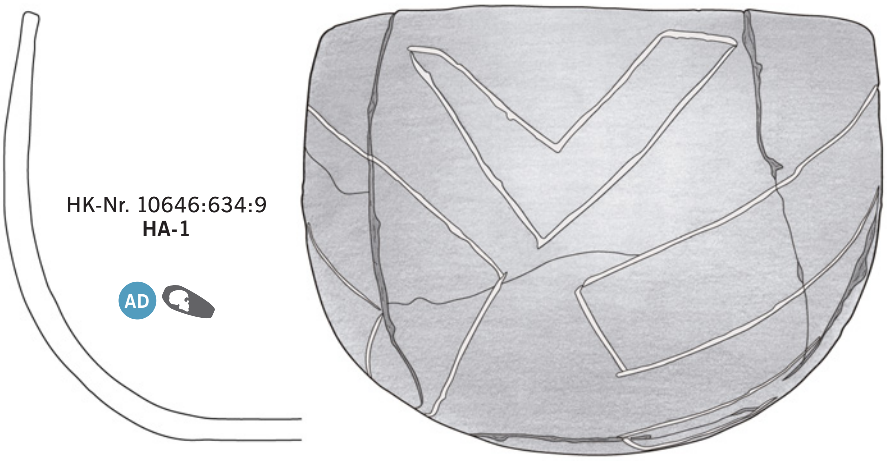

HK-Nr. 10646:634:9
HA-1

AD

Harsleben 1:2

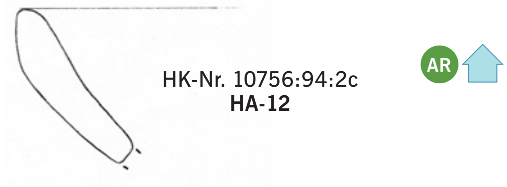

HK-Nr. 10756:94:2c
HA-12

AR

Harsleben 1:2

Schöningen group (4200–3800 BCE) – Settlement

AR 40–50+ y/♂

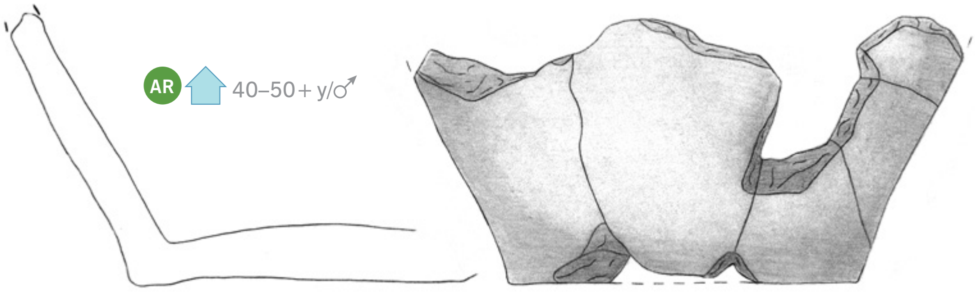

HK-Nr. 10757:11040:4c
HA-7

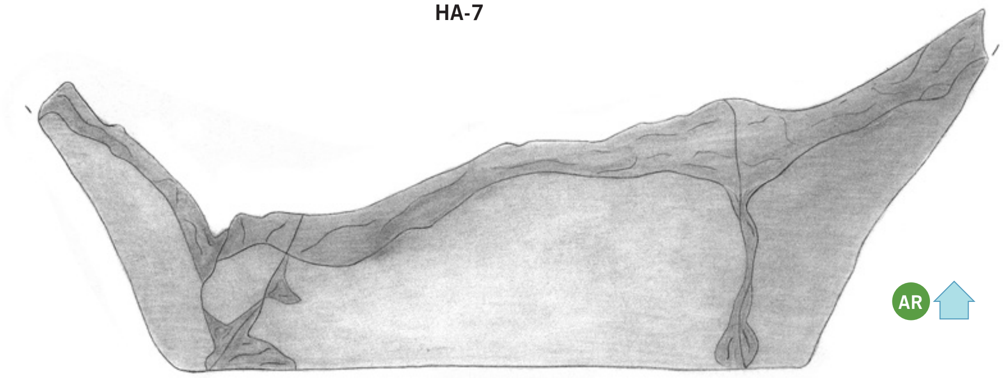

AR

HK-Nr. 10757:11040:11f+d
HA-8

Harsleben 1:2

Schöningen group (4200–3800 BCE) – Settlement

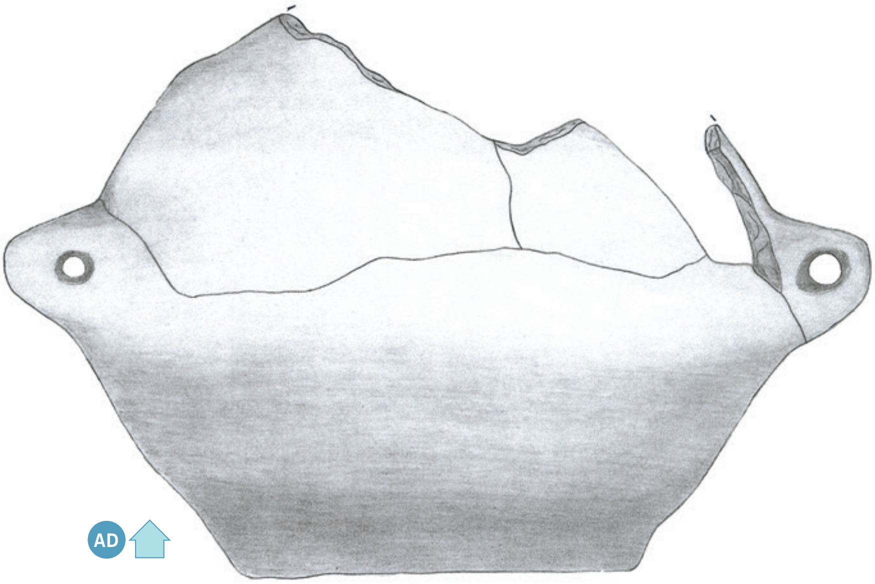

HK-Nr. 10757:11040:41a
HA-9

Harsleben

1:2

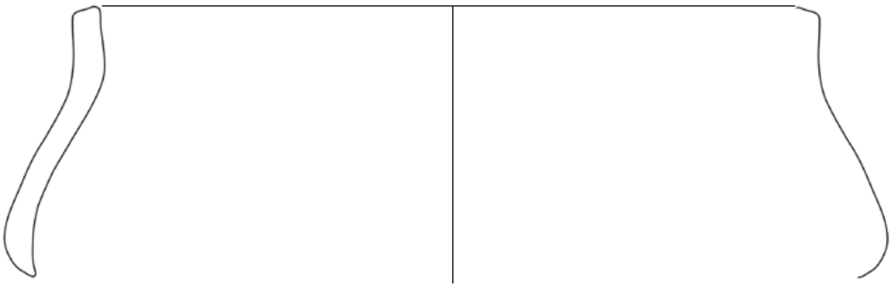

HK-Nr. 10757:11094:35
HA-10

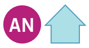

Harsleben

1:2

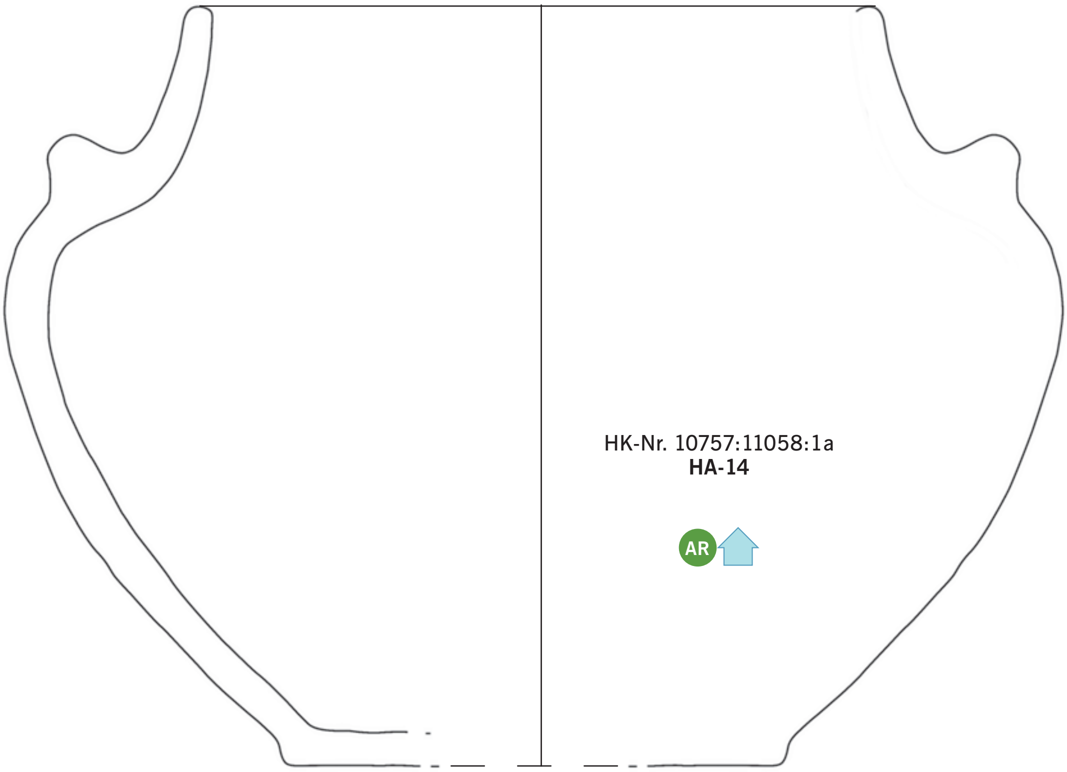

HK-Nr. 10757:11058:1a
HA-14

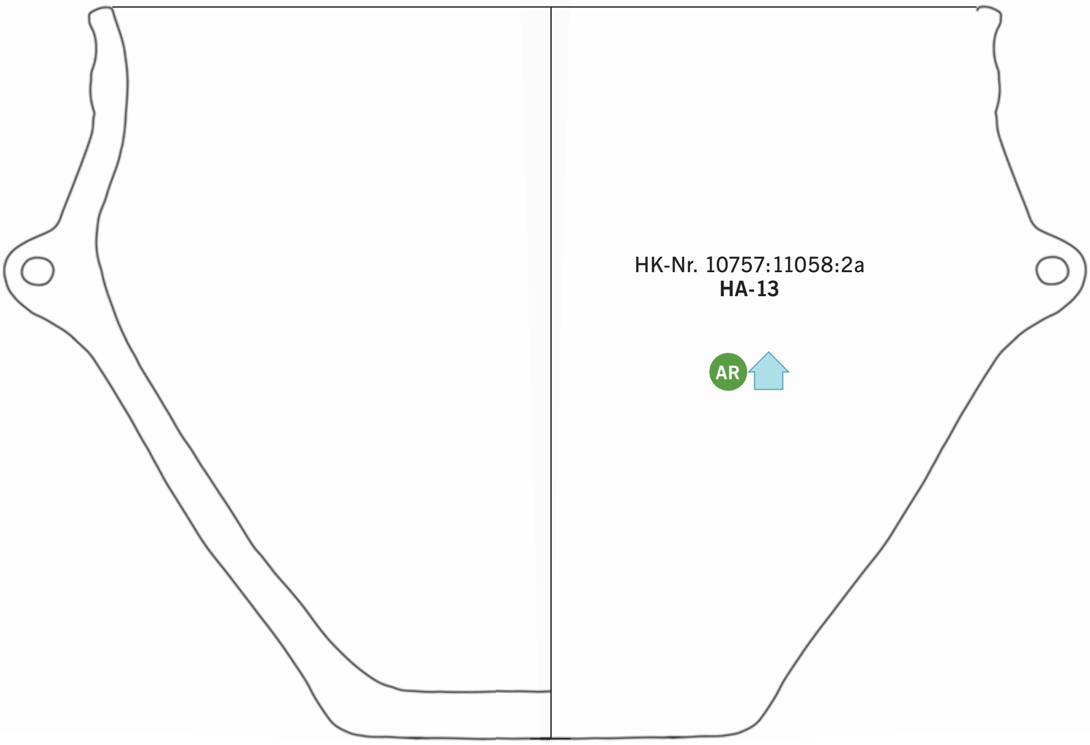

HK-Nr. 10757:11058:2a
HA-13

Harsleben

1:2

Schiepzig group (4200–3800 BCE) – Settlement

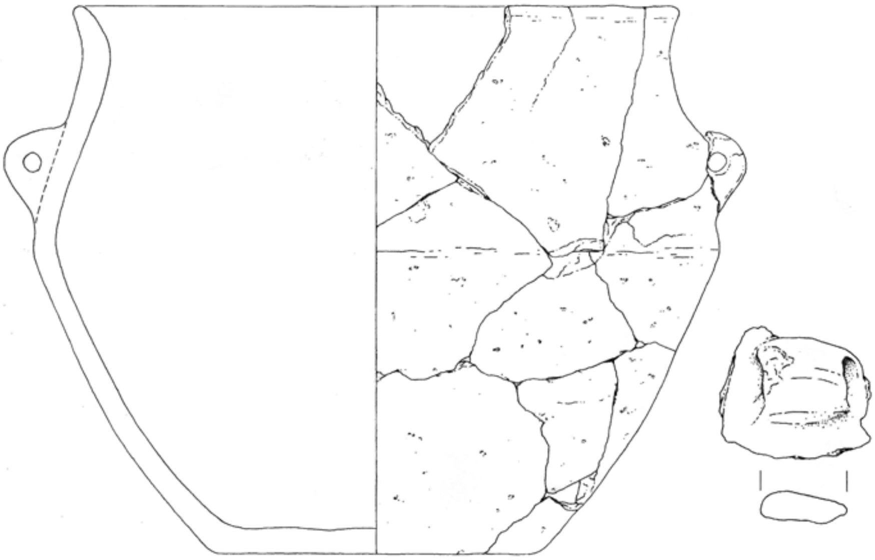

HK-Nr. 9495:1031:40a
LI-1

AN

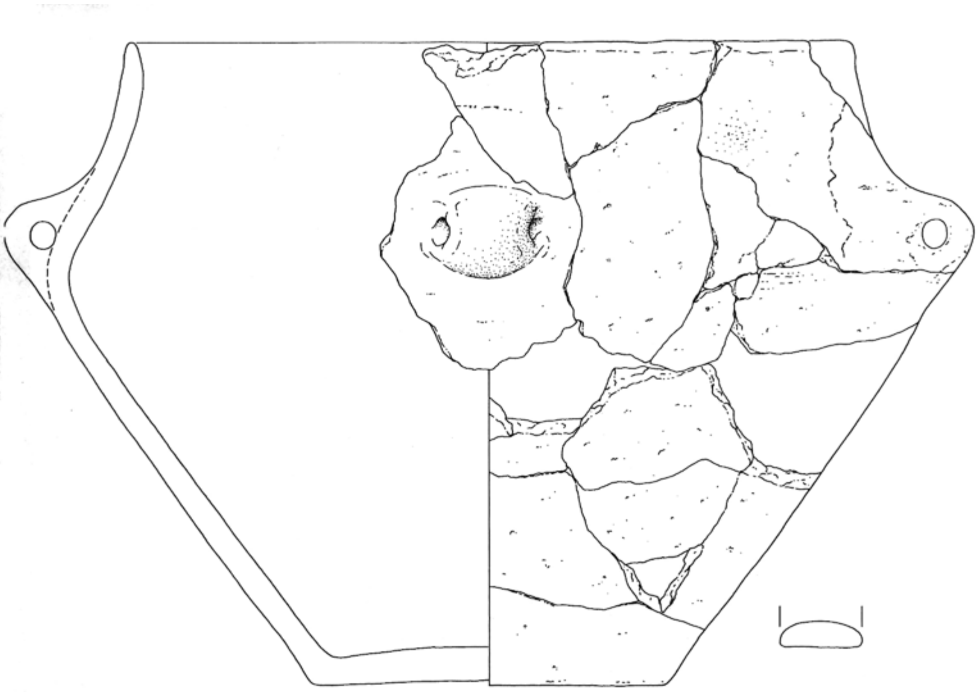

HK-Nr. 9495:1031:41a
LI-2

AN

Libehna

1:3

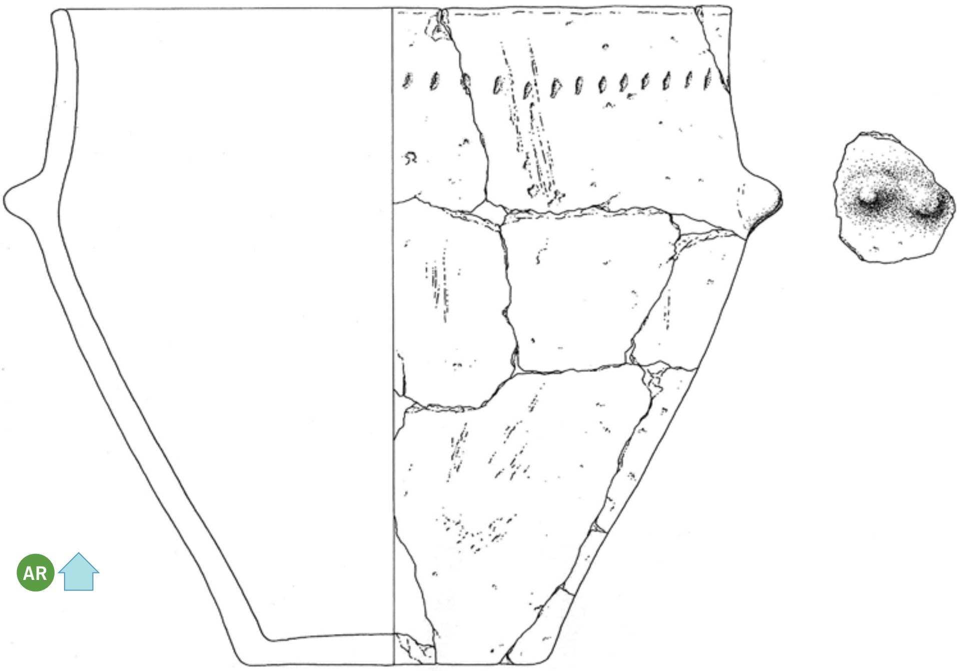

HK-Nr. 9495:1034:35a
LI-3

Libehna 1:3

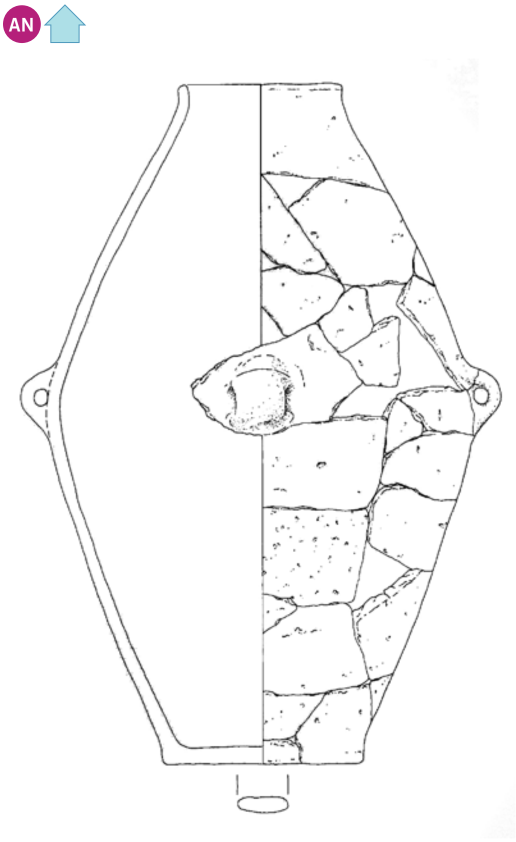

HK-Nr. 9495:2030:22
LI-4

Libehna 1:6

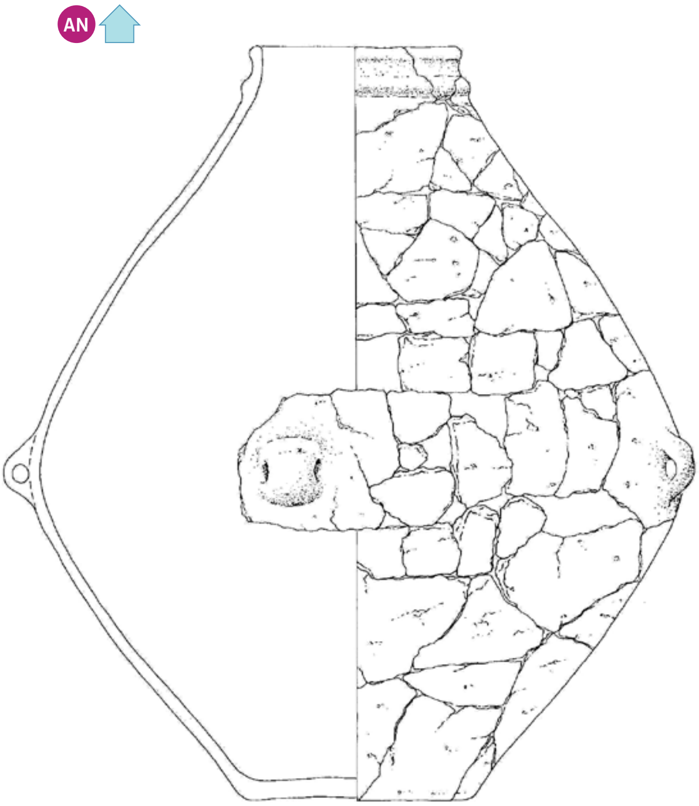

HK-Nr. 9495:2030:31
LI-5

Libehna 1:8

Baalberge Culture (3950–3375 BCE) – Settlement

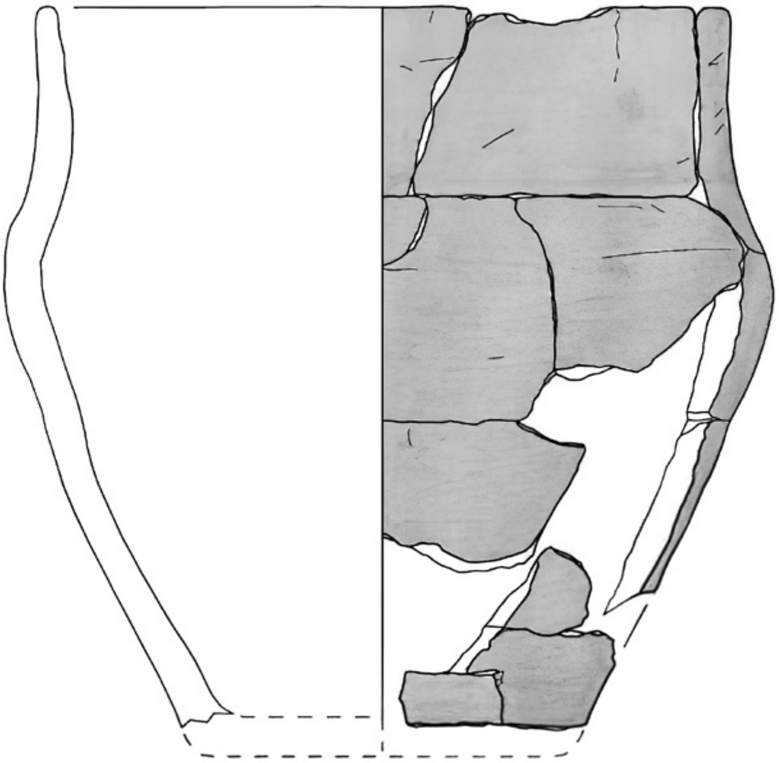

AR

HK-Nr. 12:114
BE-1

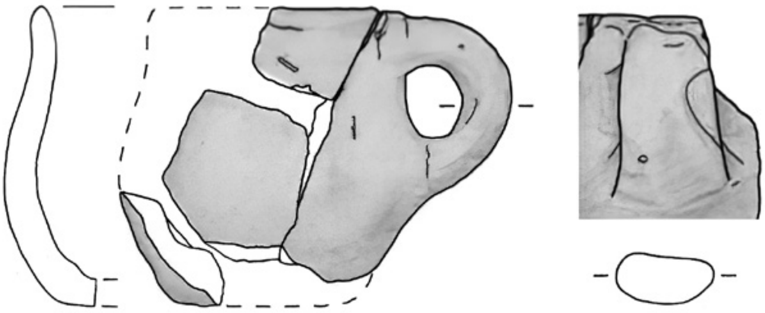

HK-Nr. 14:1068
BE-2

AR

Belleben

1:2

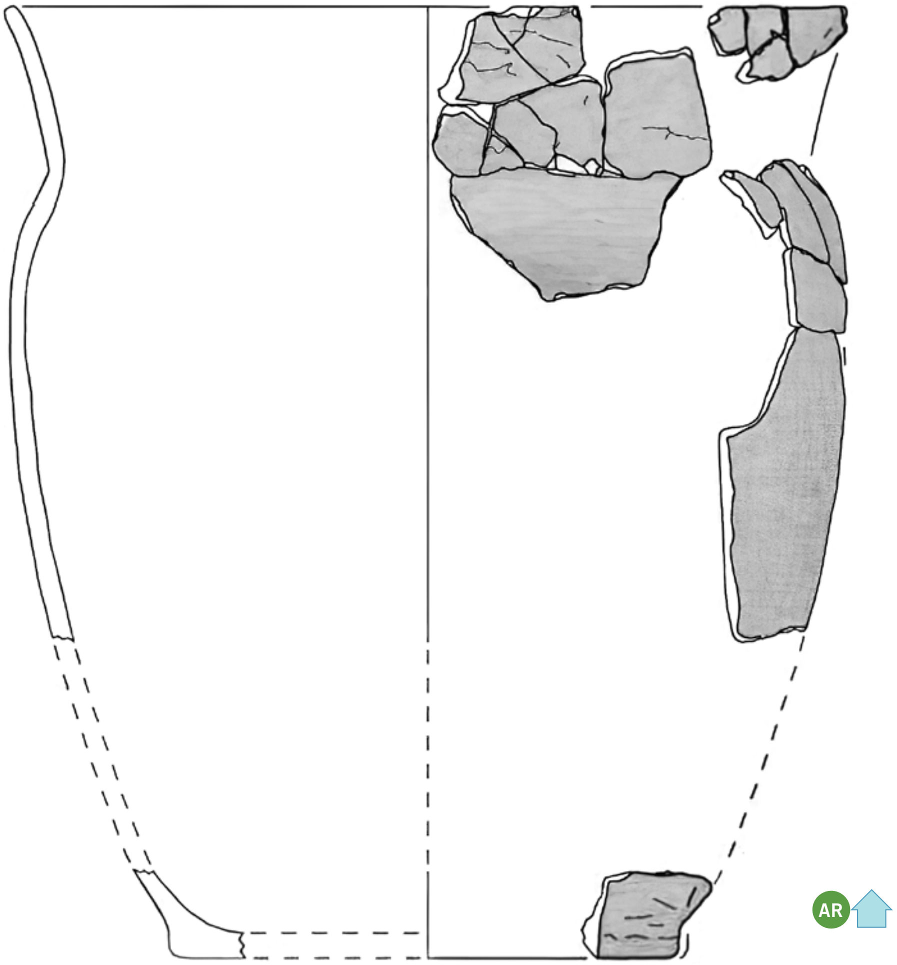

HK-Nr. 14:1146
BE-8

Belleben

1:4

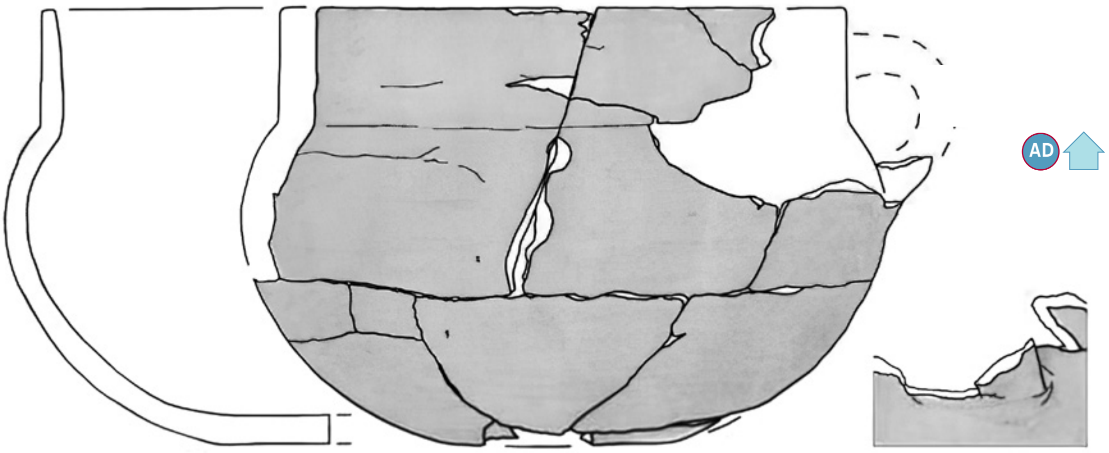

HK-Nr. 14:1054
BE-4

Belleben

1:2

Baalberge Culture (3950–3375 BCE) – Settlement

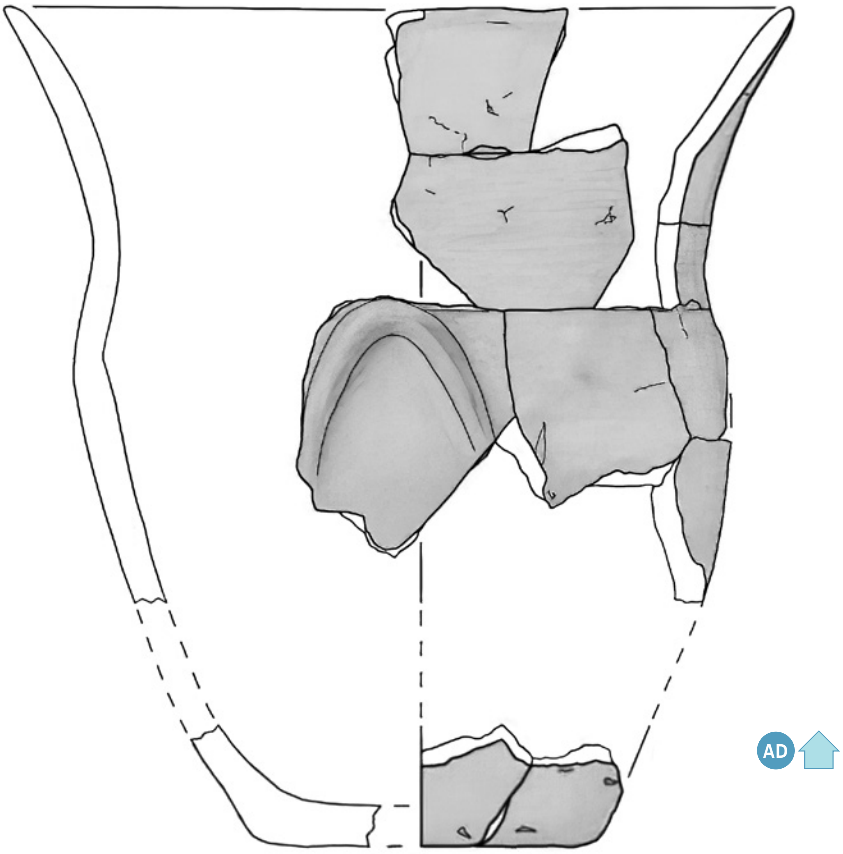

HK-Nr. 13:462
BE-3

Belleben

1:2

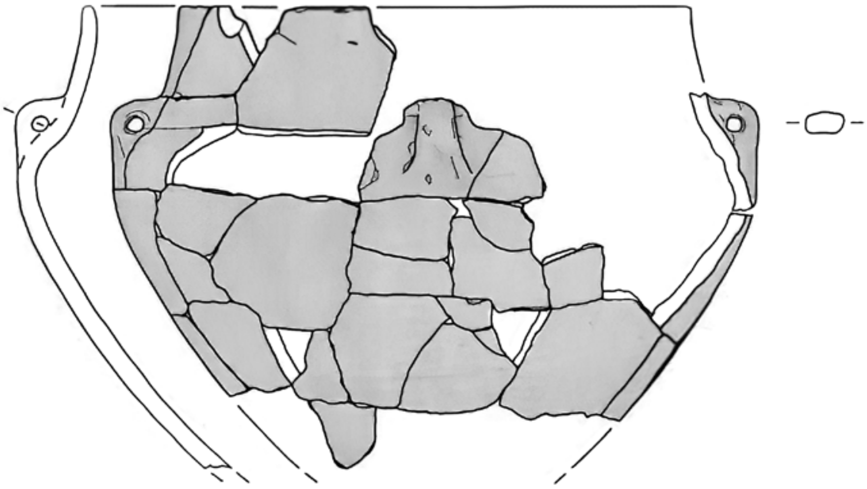

HK-Nr. 14:1054
BE-9

Belleben

1:4

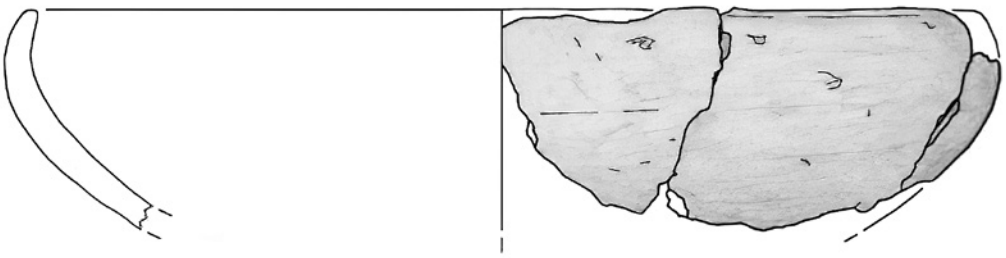

AD

HK-Nr. 1-95:1593
BE-5

Belleben

1:2

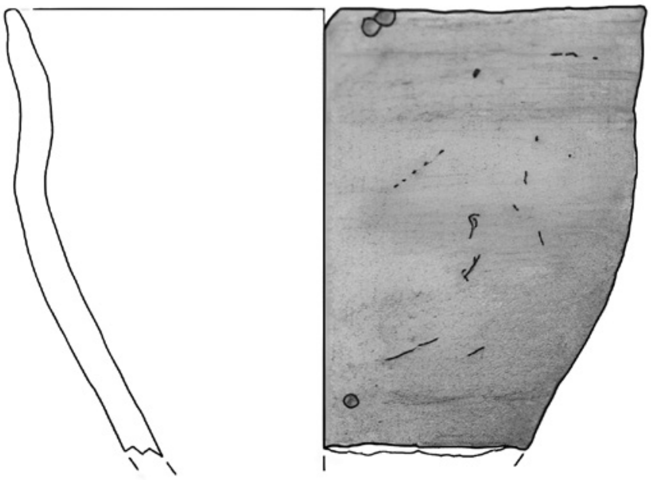

AR

HK-Nr. 13:461
BE-6

Belleben

1:2

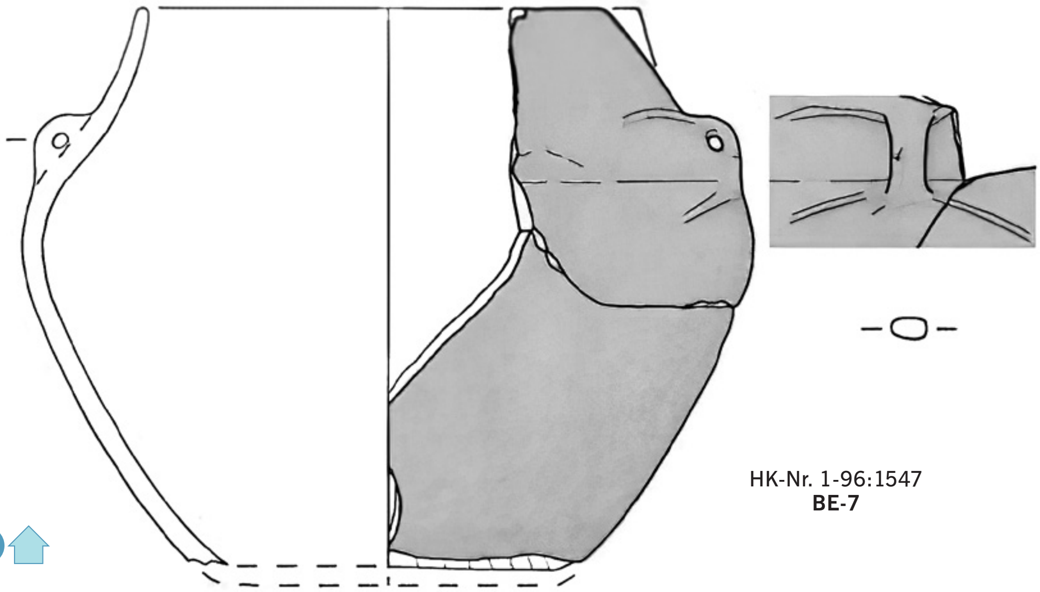

HK-Nr. 1-96:1547
BE-7

AD

Belleben

1:3

Baalberge Culture (3950–3375 BCE) – Funerary

Bef. 957 – child

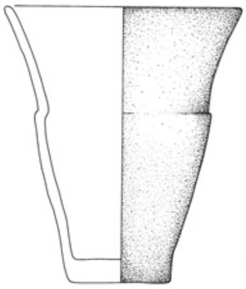

AD

HK-Nr. 2002:2481a
QU-40

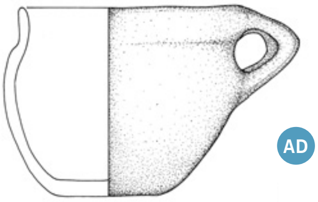

AD

HK-Nr. 2002:2481c
QU-42

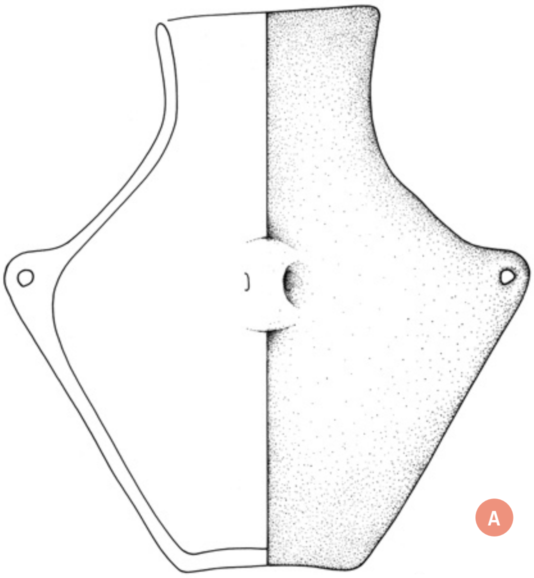

A

HK-Nr. 2002:2481b
QU-41

Queis

1:2

Bef. 961 – child or juvenile

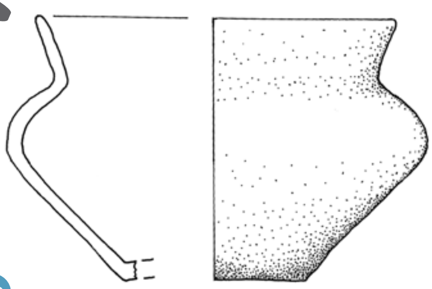

AD

HK-Nr. 2002:2483a
QU-46

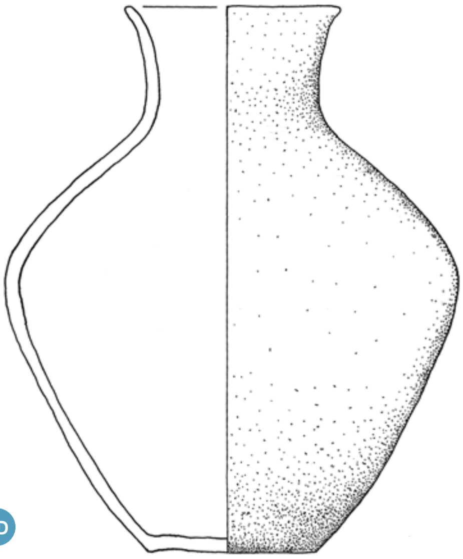

AD

HK-Nr. 2002:2483b
QU-47

Queis

1:2

Bef. 962 – child

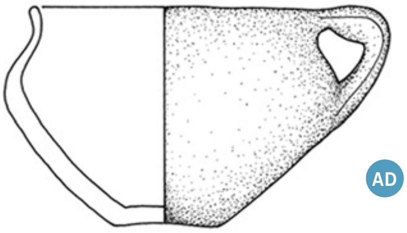

AD

HK-Nr. 2002:2484b
QU-49

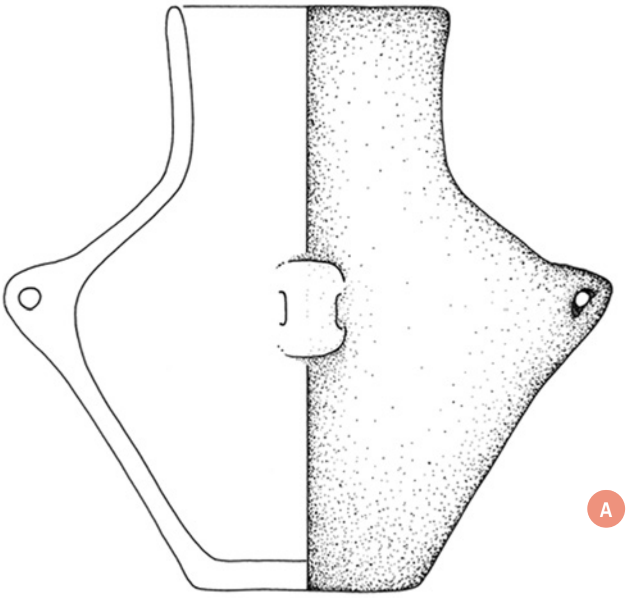

A

HK-Nr. 2002:2484a
QU-48

Queis

1:2

Bef. 960 – ♀ (mature); two children

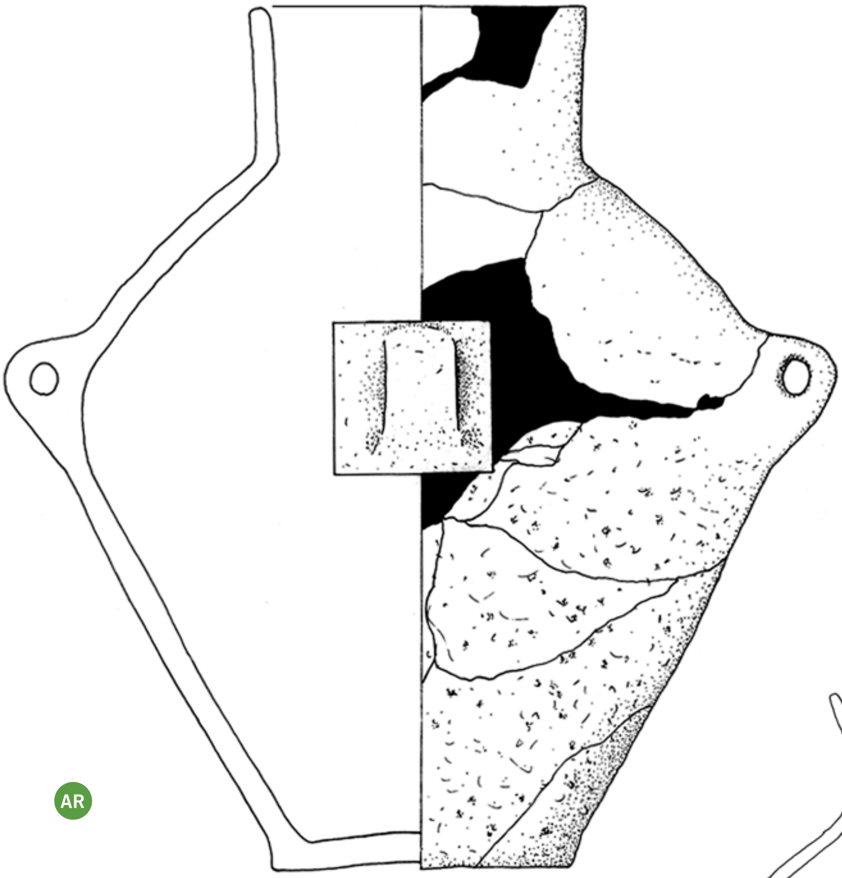

HK-Nr. 2002:2482c
QU-45

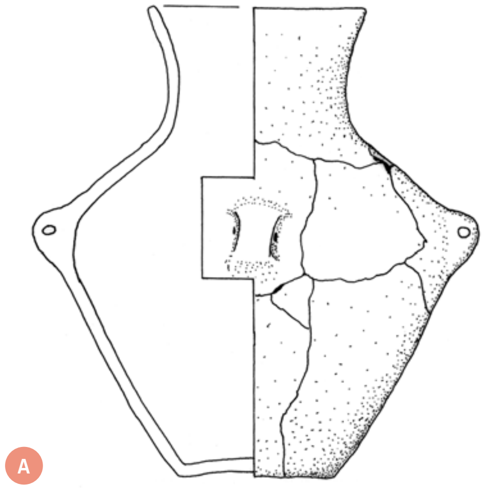

HK-Nr. 2002:2482a
QU-43

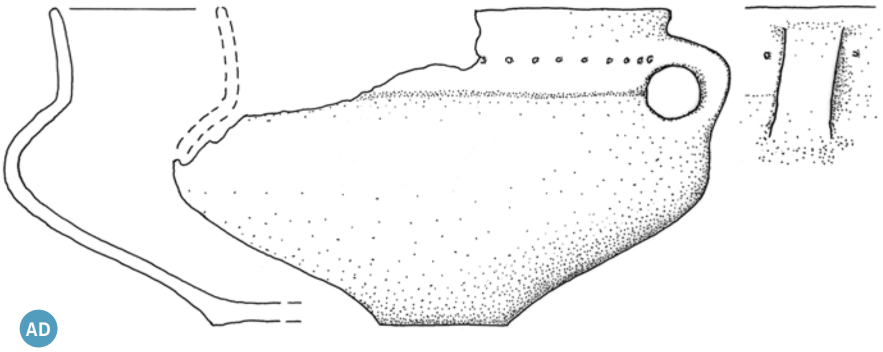

HK-Nr. 2002:2482b
QU-44

Queis

1:2

Baalberge Culture (3950–3375 BCE) – Funerary

20–30 y

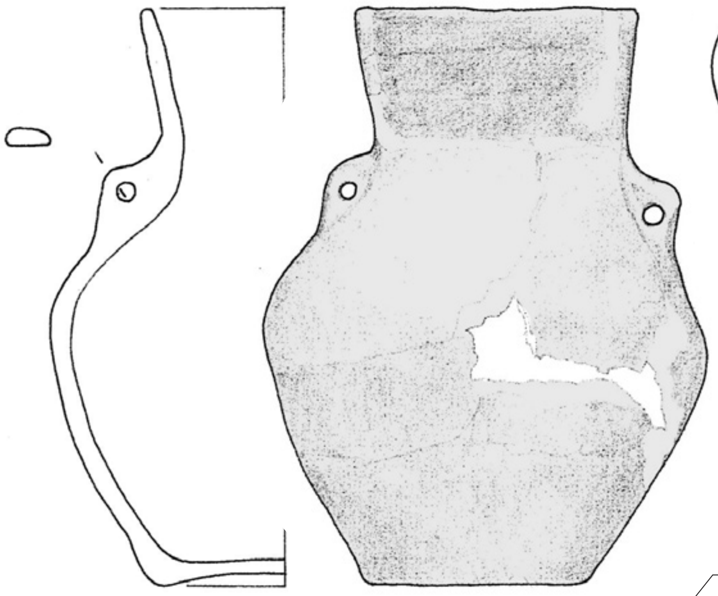

HK-Nr. 13476:2970:1a
PO-14

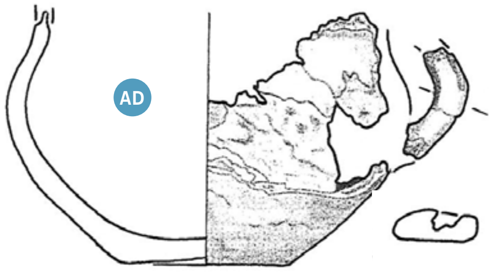

HK-Nr. 13476:2970:2a
PO-15

1:2

Pömmelte

?

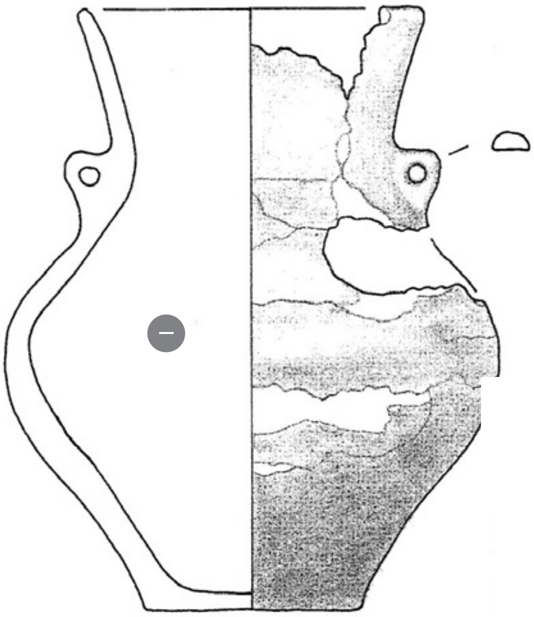

HK-Nr. 12820:2574:1a
PO-13

Pömmelte

1:2

40–50 y/♂

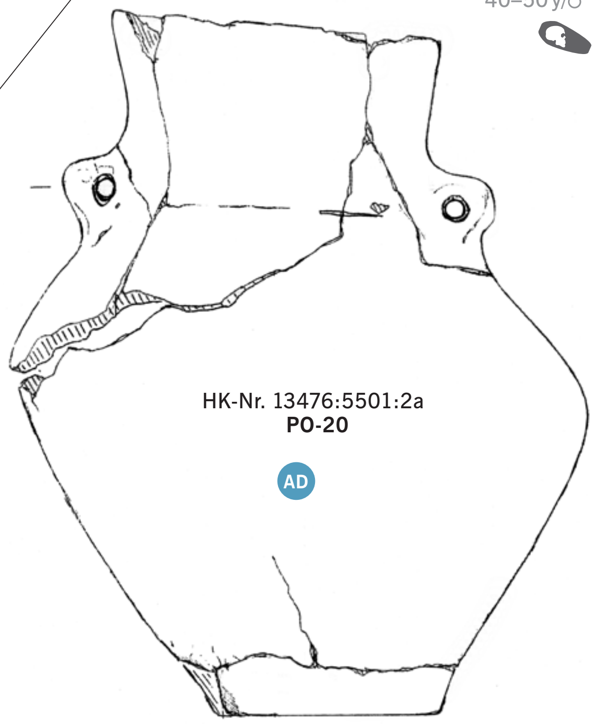

HK-Nr. 13476:5501:2a
PO-20

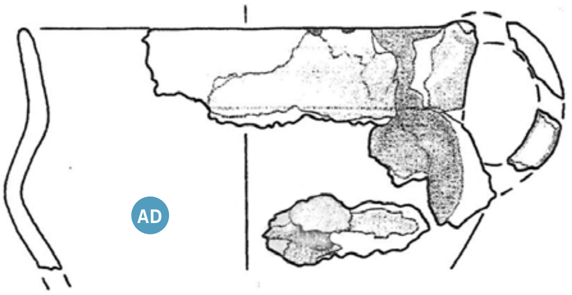

HK-Nr. 13476:5501:2b
PO-19

1:2

Corded Ware (2575–2200 BCE) – Funerary

16–18 y/♀

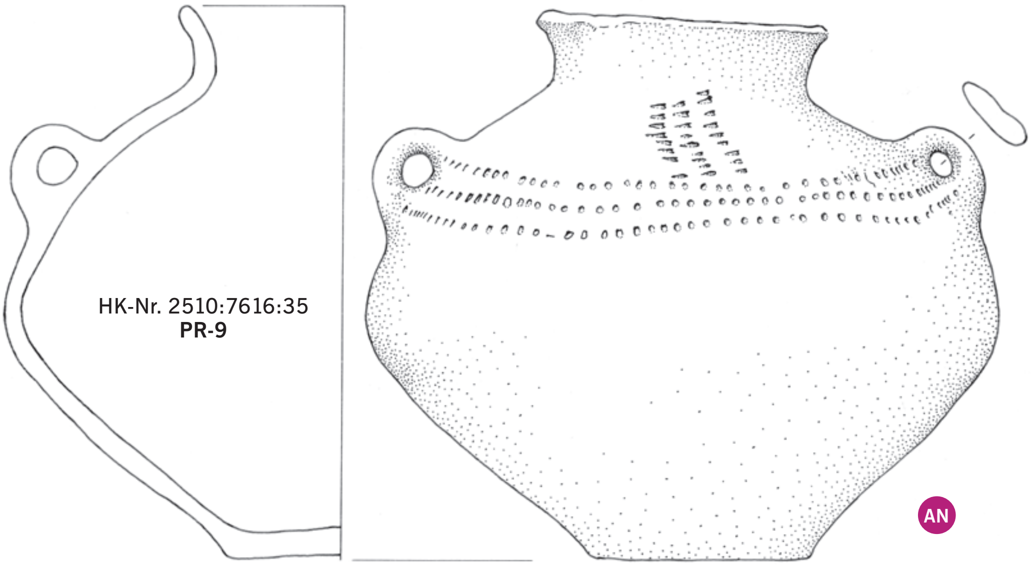

HK-Nr. 2510:7616:35
PR-9

AN

1:3

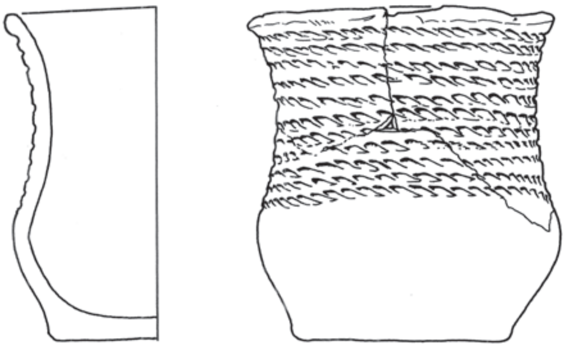

AD

HK-Nr. 2510:7616:43
PR-10

Profen

1:2

8–12y

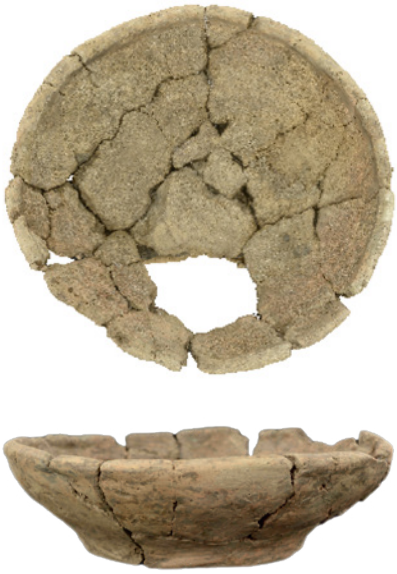

AR

HK-Nr. 2510:5068:7
PR-5

Profen

HK-Nr. 2510:5068:8
PR-6

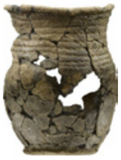

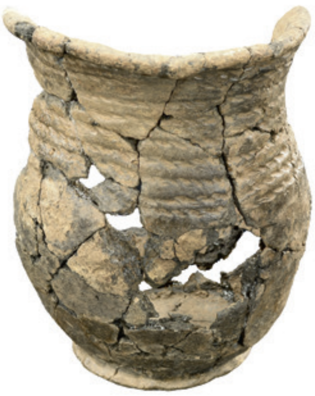

AR

1:3

Corded Ware (2575–2200 BCE) – Funerary

25–30 y/♀

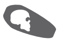

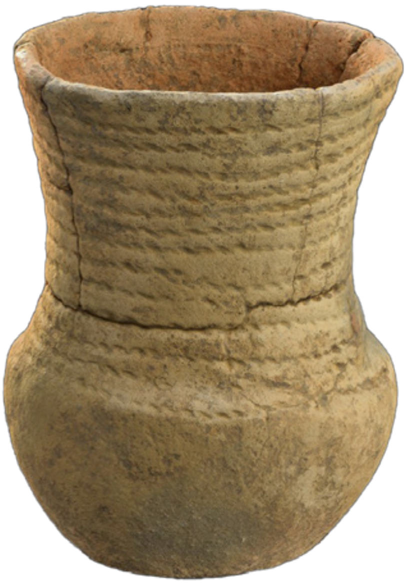

AR

HK-Nr. 2510:7868:34a
PR-17

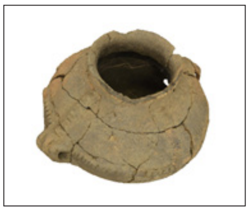

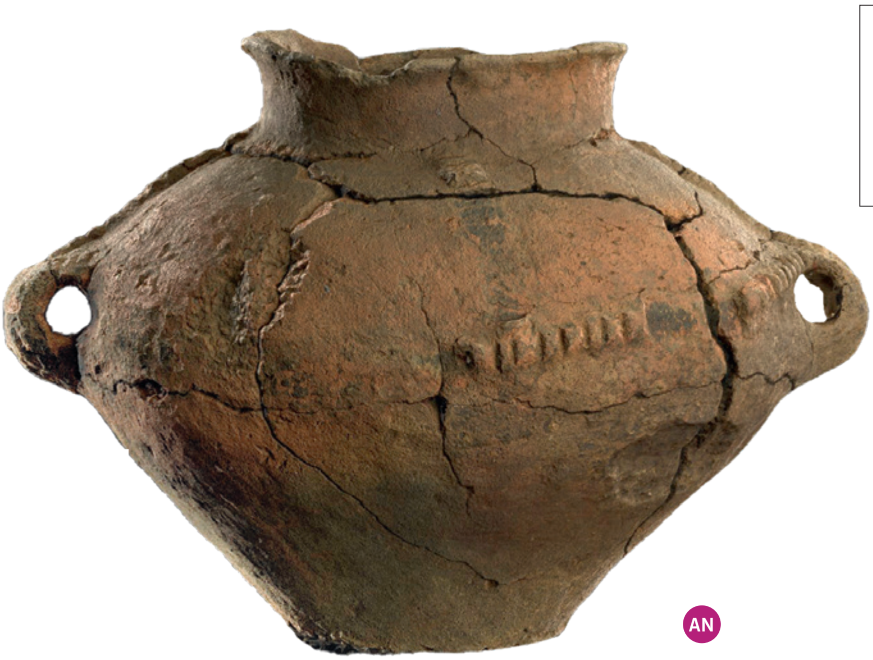

AN

HK-Nr. 2510:7868:33a
PR-16

Profen

1:2

30–50 y/♂

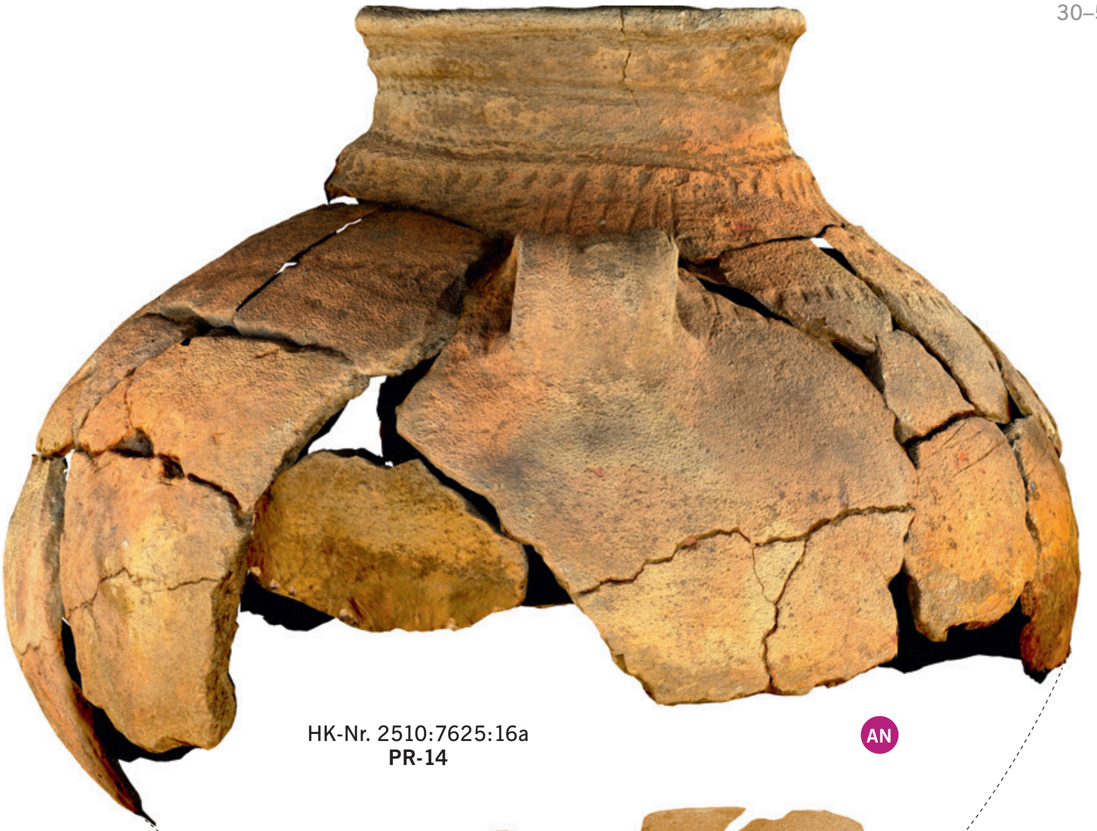

HK-Nr. 2510:7625:16a
PR-14

AN

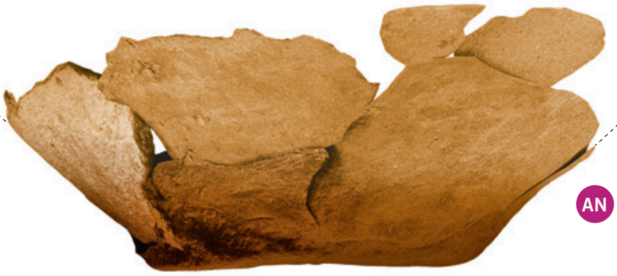

HK-Nr. 2510:7625:17a
PR-15

AN

Profen

1:2

Adult/♂

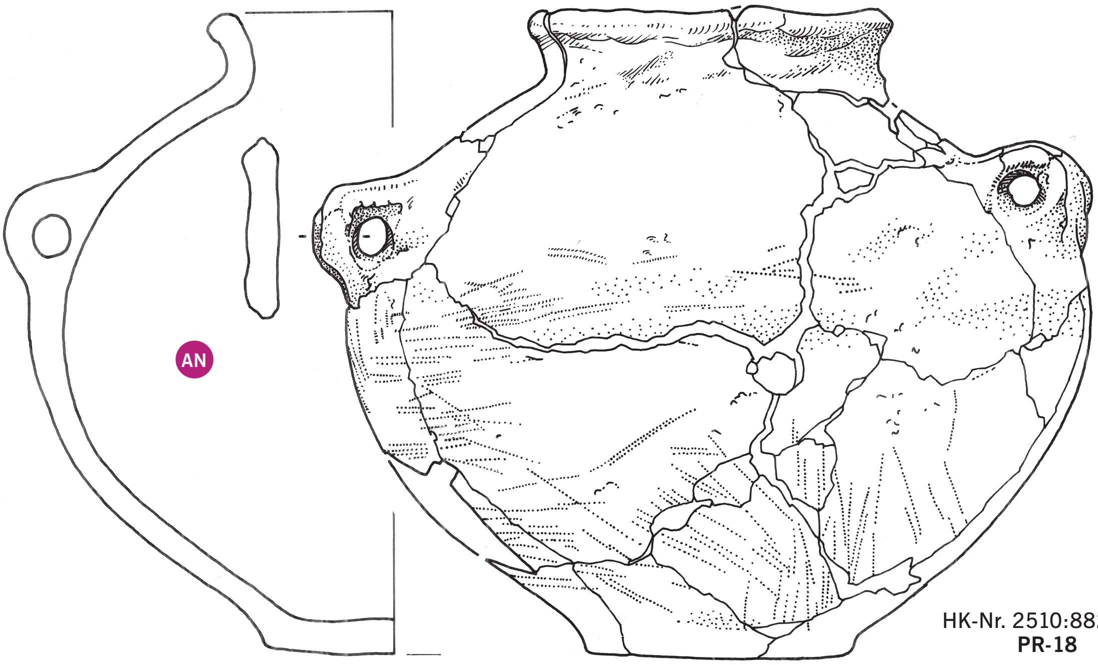

AN

HK-Nr. 2510:8821:11
PR-18

Profen

1:2

Corded Ware (2575–2200 BCE) – Funerary

16–25 y

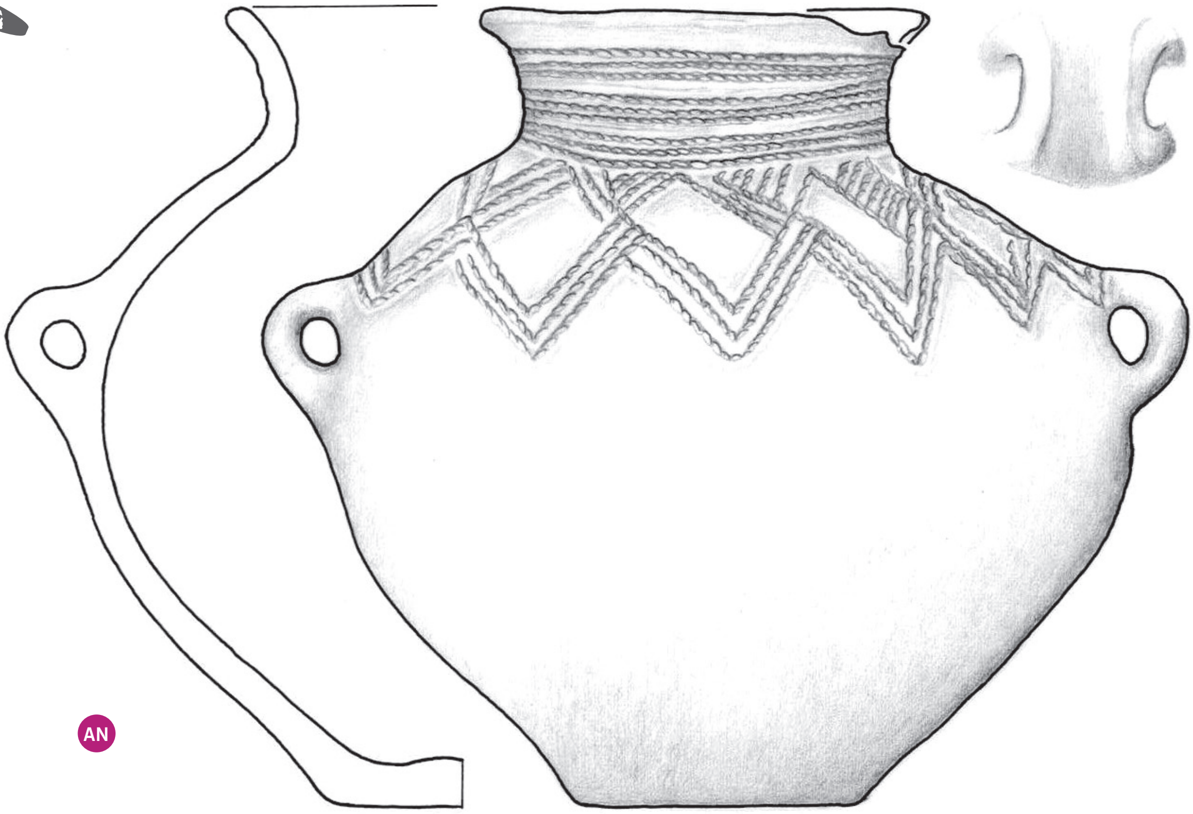

HK-Nr. 2510:7618:16
PR-11

AR V

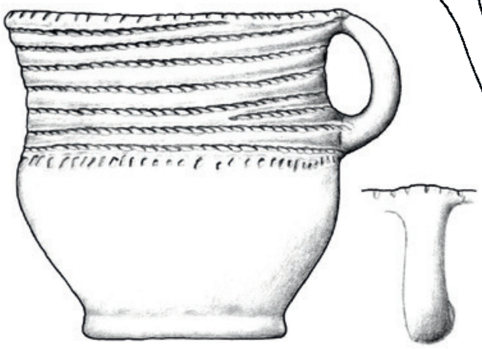

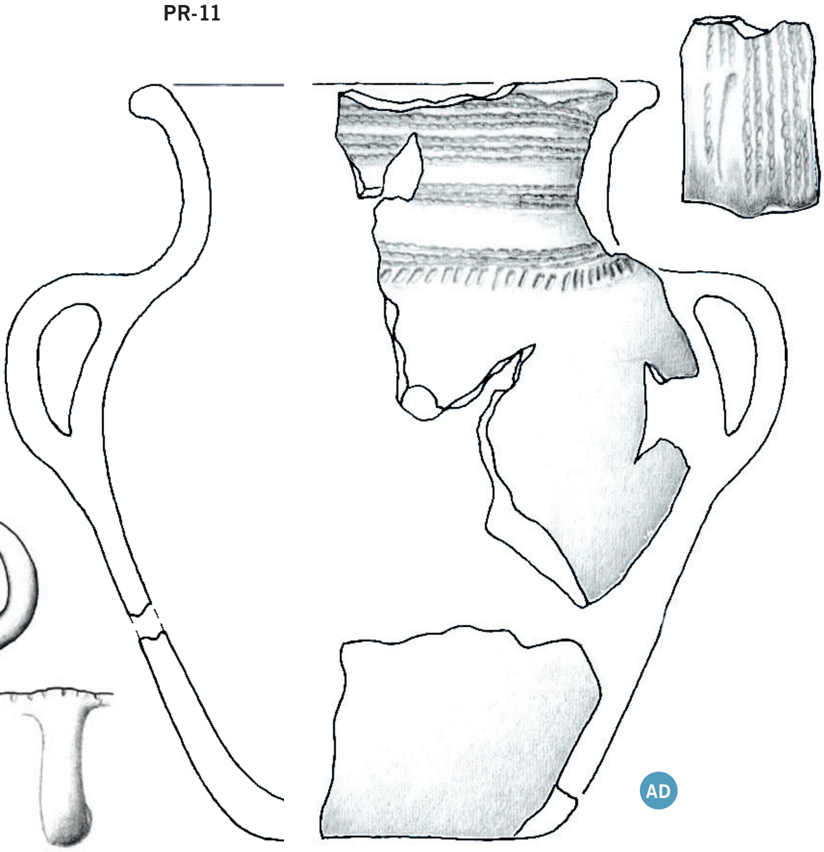

HK-Nr. 2510:7618:18
PR-13

HK-Nr. 2510:7618:17
PR-12

Profen

1:2

Adult

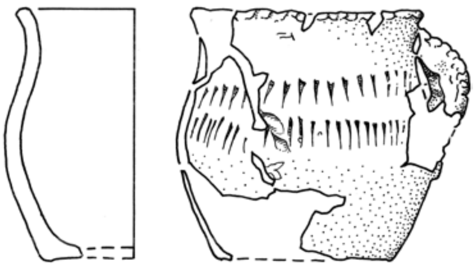

HK-Nr. 2510:5043:23
PR-1
AR

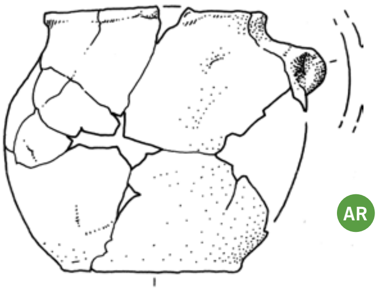

AR

HK-Nr. 2510:5043:24
PR-2

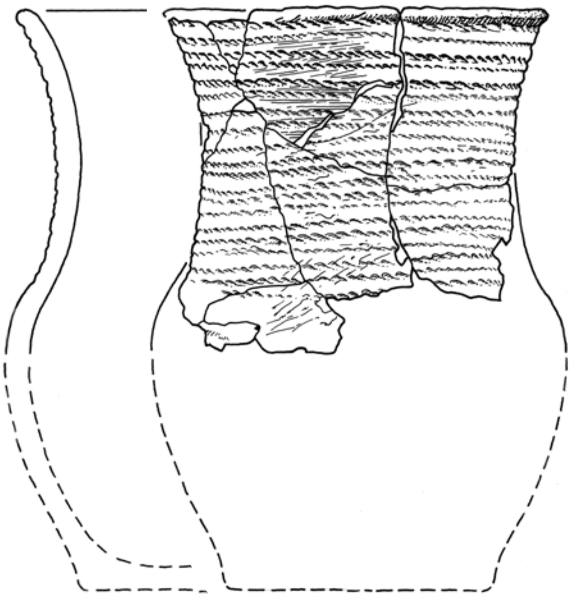

HK-Nr. 2510:5043:25
PR-3
AR

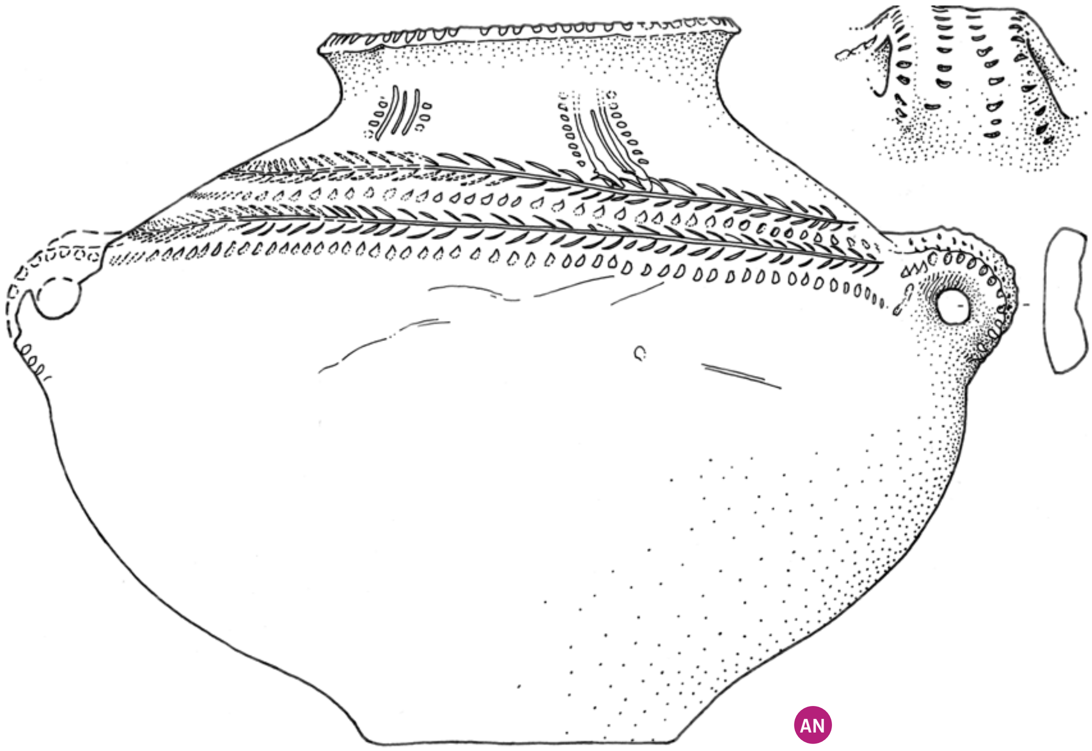

AN

HK-Nr. 2510:5043:27
PR-4

Profen

1:2

Corded Ware (2575–2200 BCE) – Funerary and Deposit

Adult–mature/♀

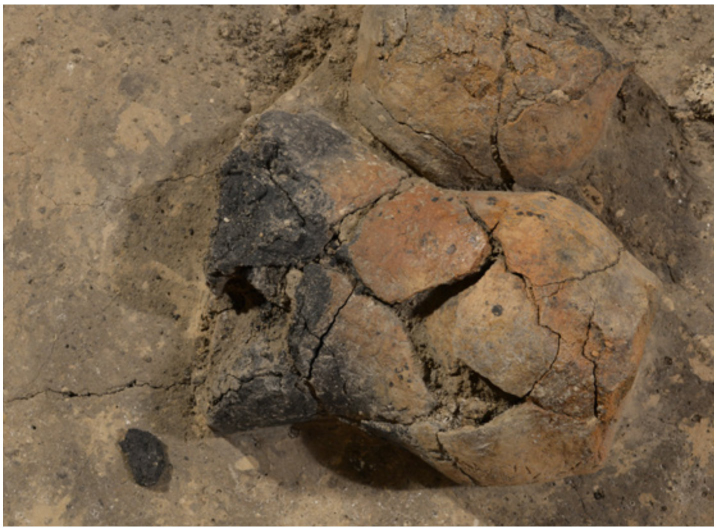

HK-Nr.
2671:25815:38
OE-7

AN

Oechlitz

no scale

40–50 y/♂

AR

HK-Nr. 3125:1581:7
BR-1

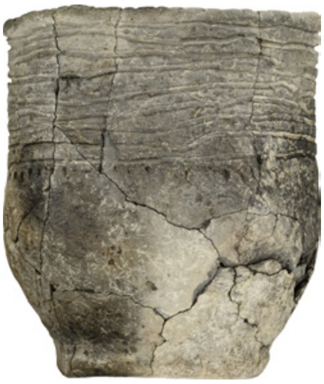

Bernburg

1:2

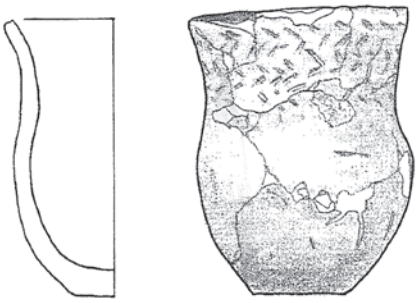

HK-Nr. 13476:5638:1a
PO-32

AD

Pömmelte

1:2

40–50 y/♂

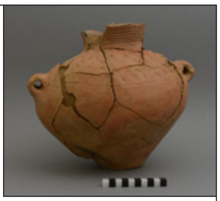

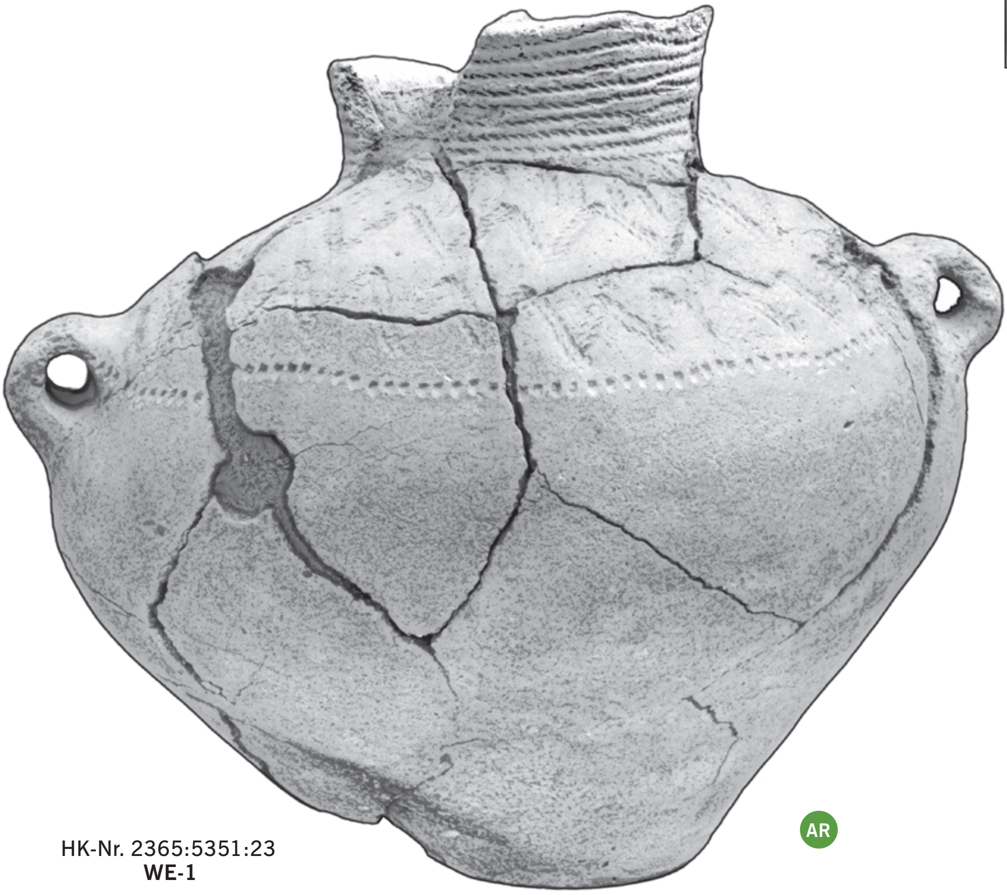

AR

HK-Nr. 2365:5351:23
WE-1

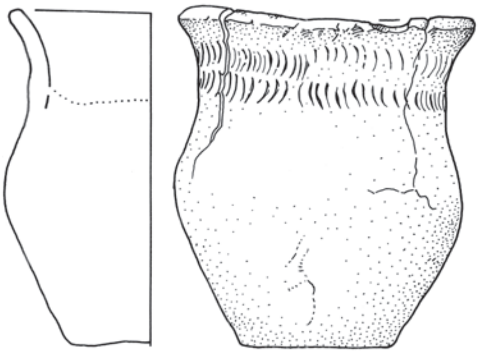

HK-Nr. 2365:5351:49a
WE-2

AD

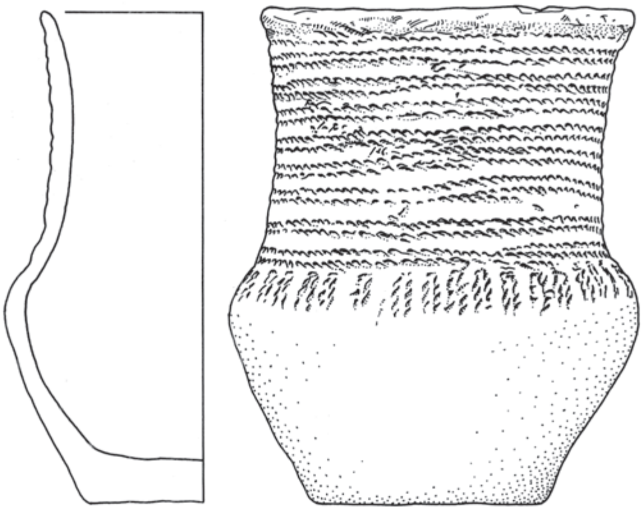

HK-Nr. 2365:5351:50a
WE-3

AD

Wennungen

1:2

Corded Ware (2575–2200 BCE) – Funerary

multiple burial (5 ind.)

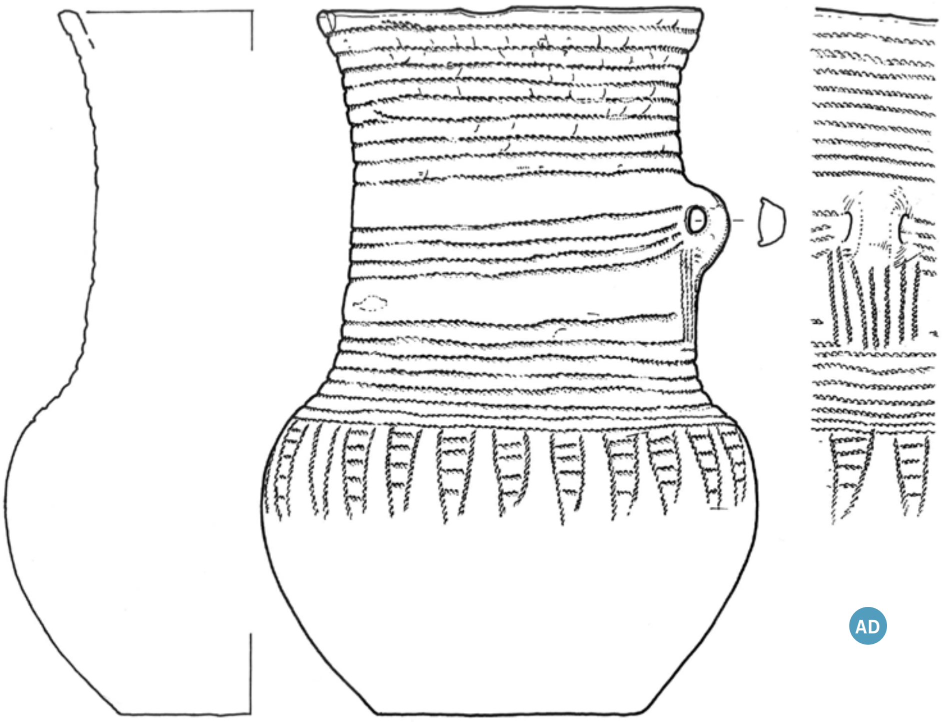

HK-Nr. 2671:25645:104
OE-4

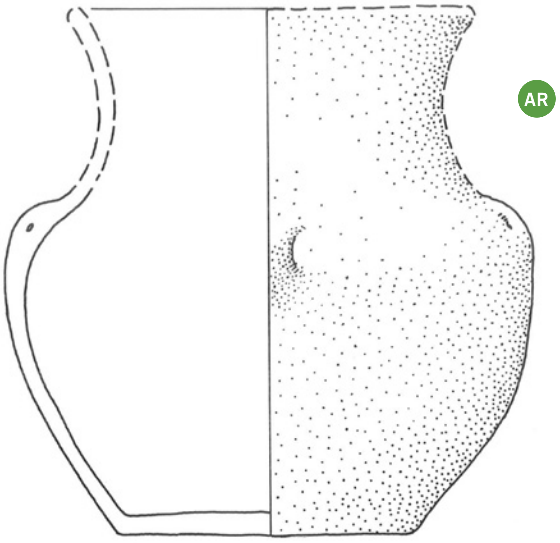

HK-Nr. 2671:25645:75 and 70
OE-5 and 6

Oechlitz

1:2

25–30 y/♀

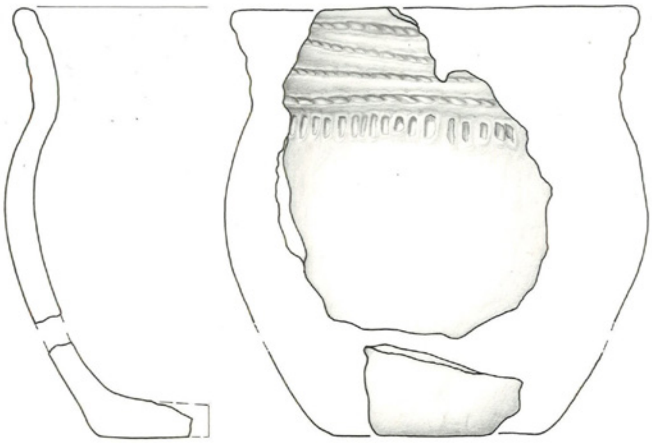

AR

HK-Nr. 2671:25355:26
OE-1

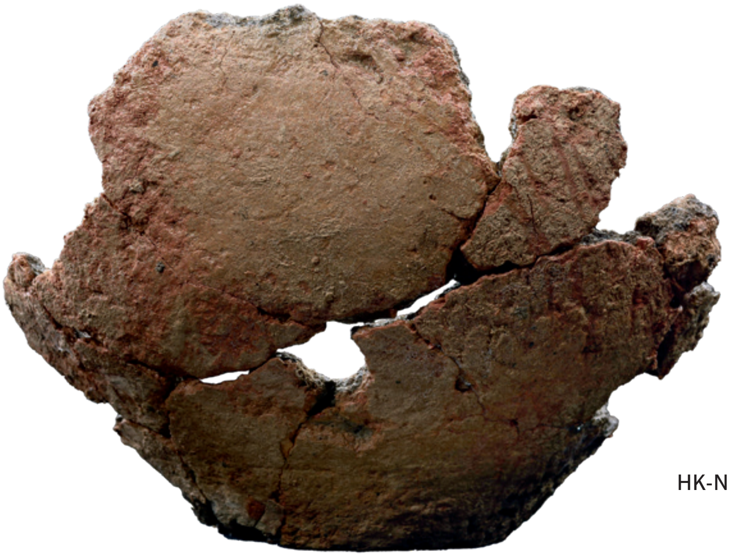

AN

HK-Nr. 2671:25355:27
OE-2

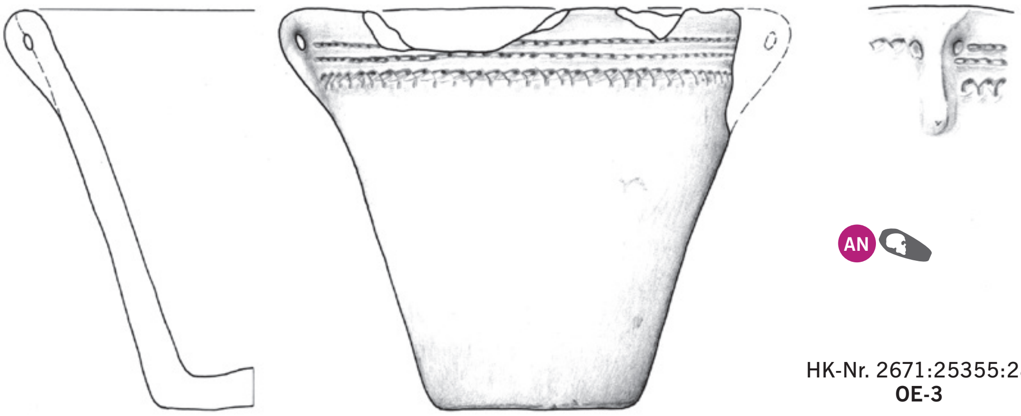

AN

HK-Nr. 2671:25355:28
OE-3

Oechlitz

1:2

Bell Beaker Culture (2500–2200 BCE) – Funerary

35-45 y/♂ + Infans II

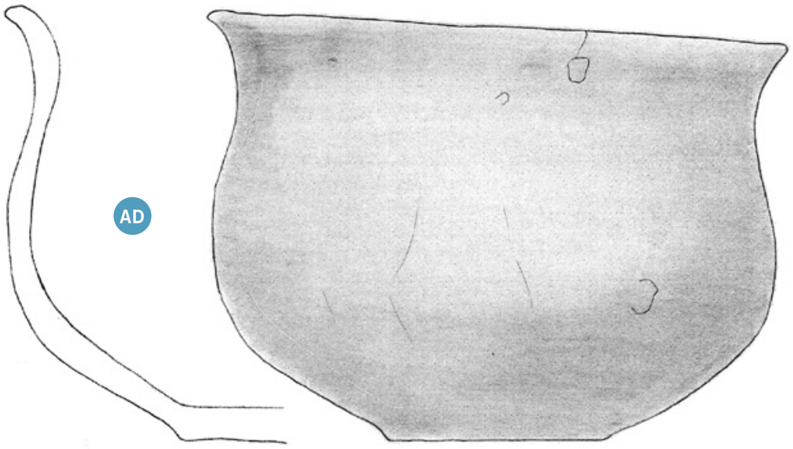

HK-Nr. 12820:1750:2a
PO-37

Pömmelte

1:2

20–30 y

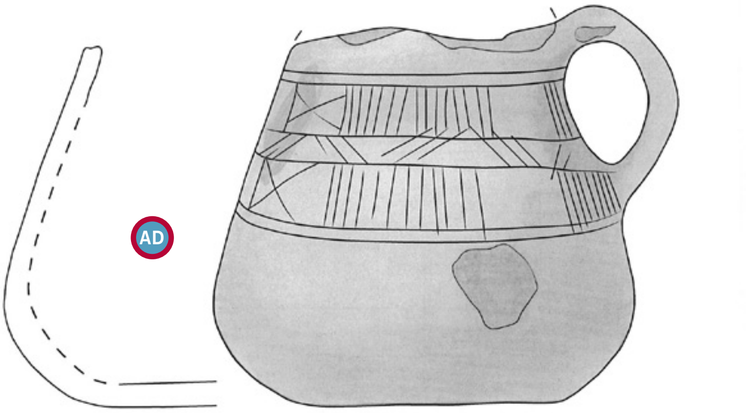

HK-Nr. 12820:1742:1a
PO-38

Pömmelte

1:2

25–30 y/♀ + Infans I+II (6y + 4y)

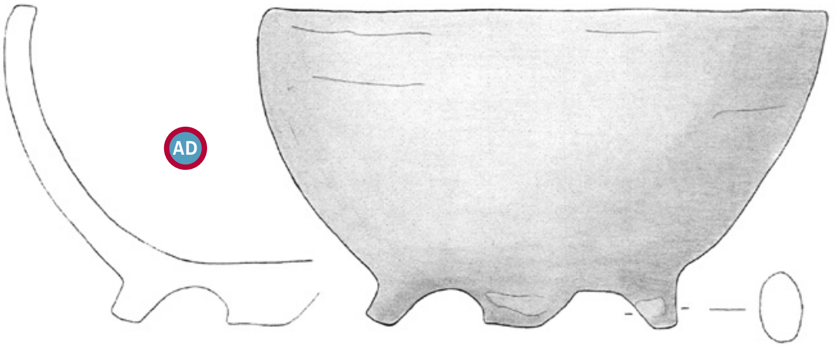

HK-Nr. 12820:1567:1
PO-34

Pömmelte

1:2

20–50 y

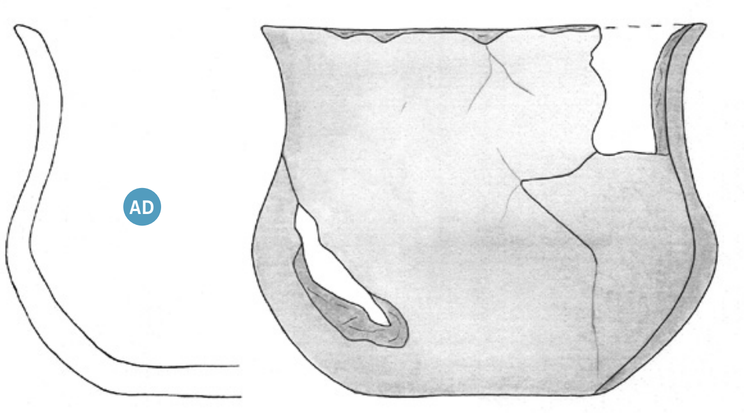

HK-Nr. 12820:1454:1a
PO-35

Pömmelte

1:2

juvenil–subadult

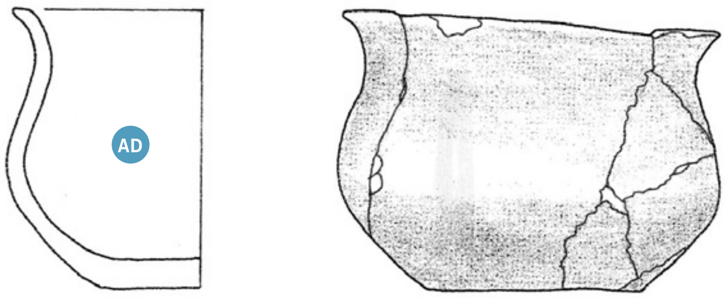

HK-Nr. 12820:1886:2a
PO-36

Pömmelte

1:2

16–20 y

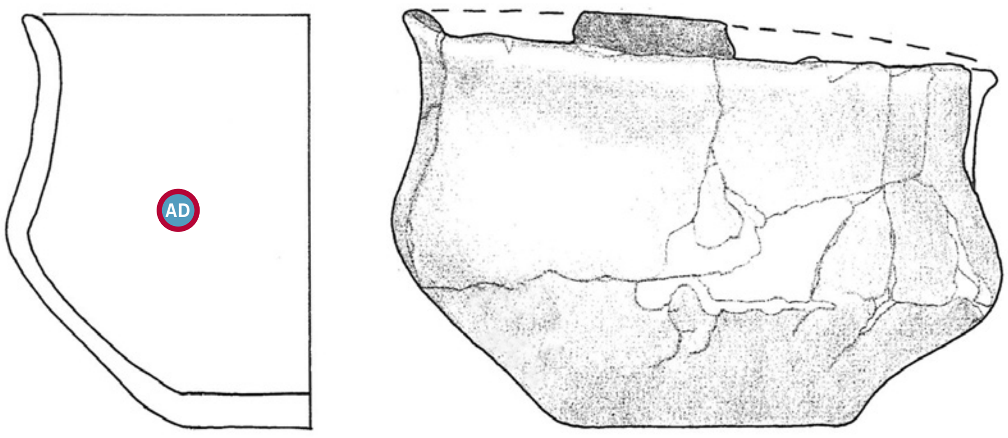

HK-Nr. 13476:4207:1a
PO-18

Pömmelte

1:2

Bell Beaker Culture (2500–2200 BCE) – Funerary

40–55 y

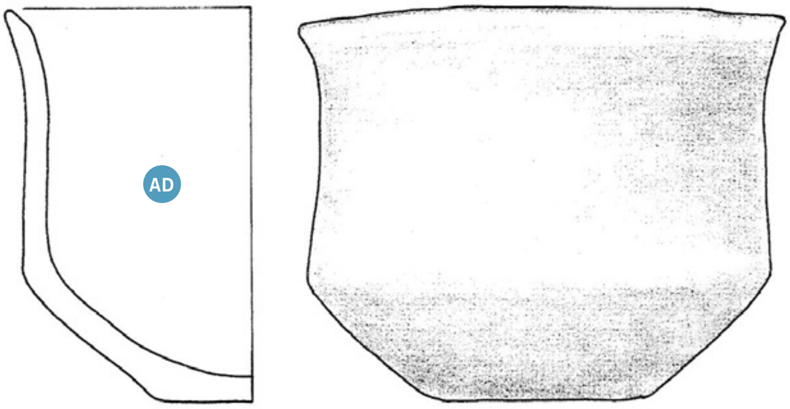

HK-Nr. 13476:5643:4a
PO-21

Pömmelte

1:2

20–30 y/♂ + Infans I

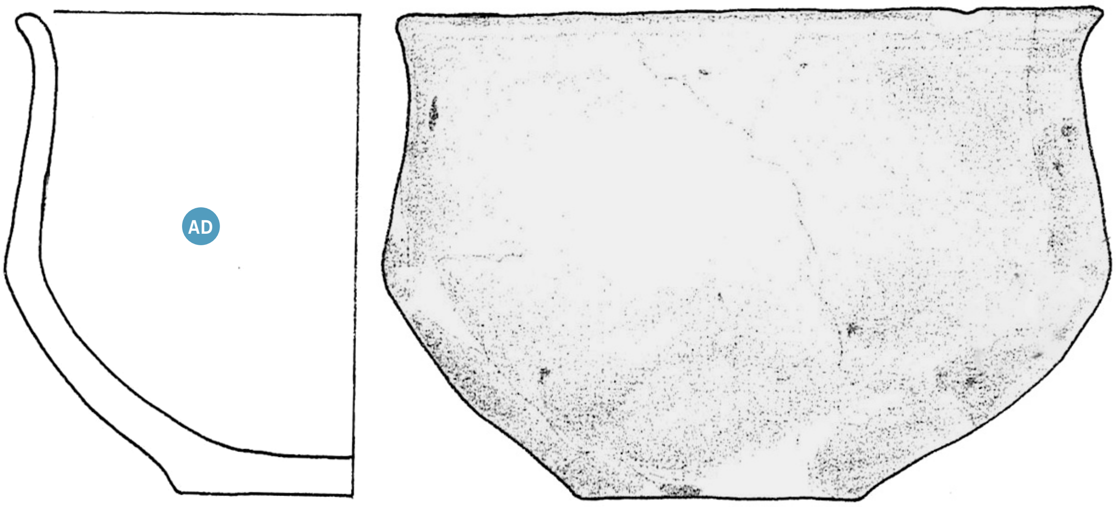

HK-Nr.
13476:4200:2a
PO-17

Pömmelte

1:3

Bell Beaker Culture (2500–2200 BCE) – Funerary

30–50 y/♀

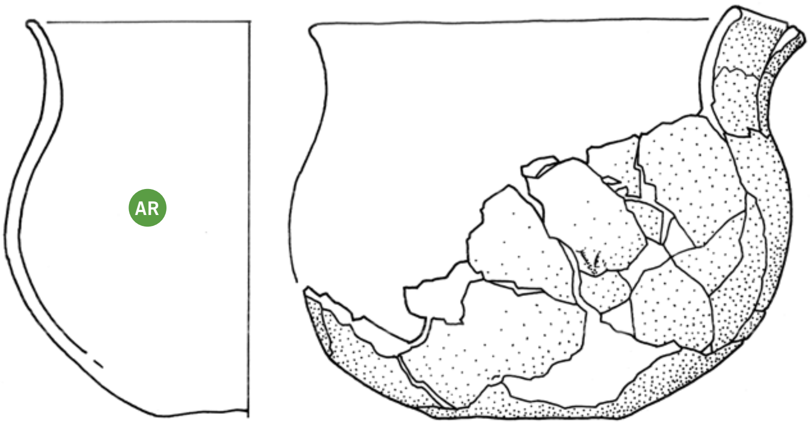

HK-Nr. 2510:5427:21
PR-7

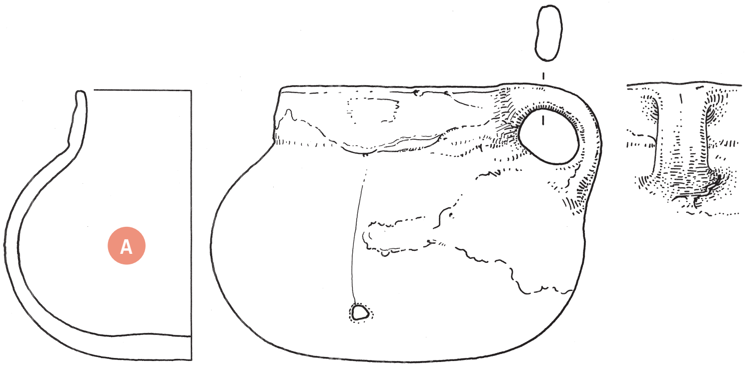

HK-Nr. 2510:5427:22
PR-8

Profen 1:2

Únětice Culture (2200–1775 BCE) – Funerary

?

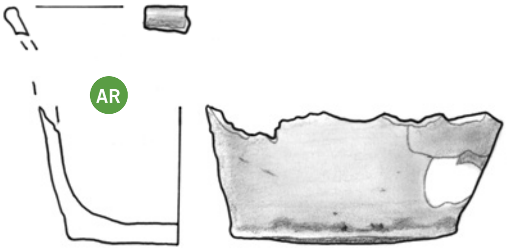

HK-Nr. 13476:3298:1
PO-16

Pömmelte 1:2

20–25 y

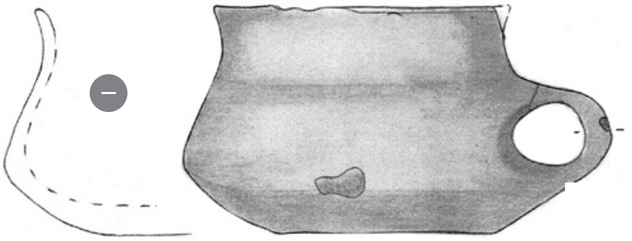

HK-Nr. 13476:2702:1a
PO-39

Pömmelte 1:2

Únětice Culture (2200–1775 BCE) – Settlement

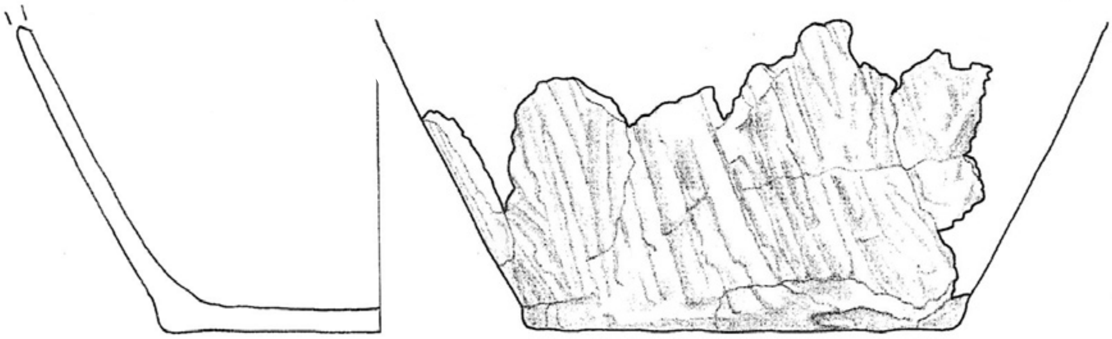

HK-Nr. 12820:2372:1a
PO-12

Pömmelte

1:4

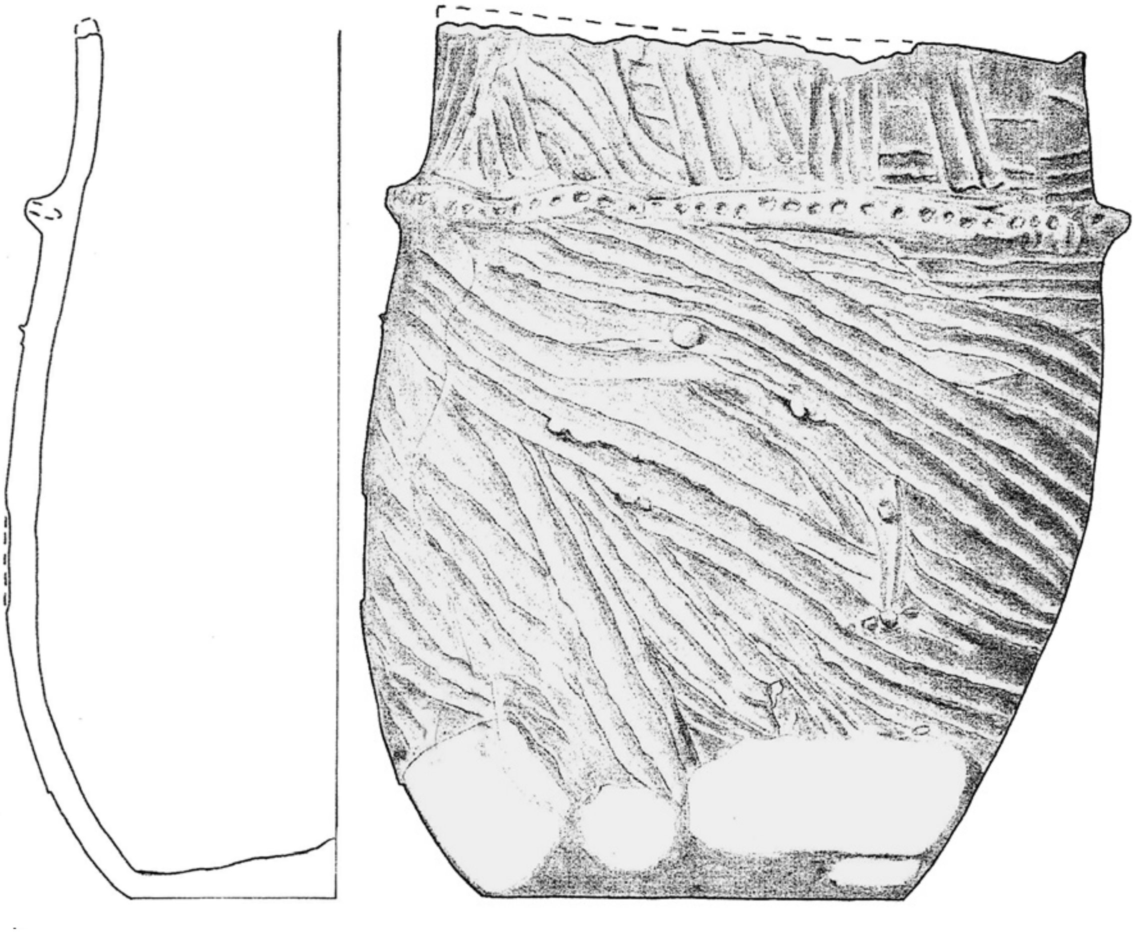

HK-Nr. 13476:2974:1a
PO-33

Pömmelte

1:4

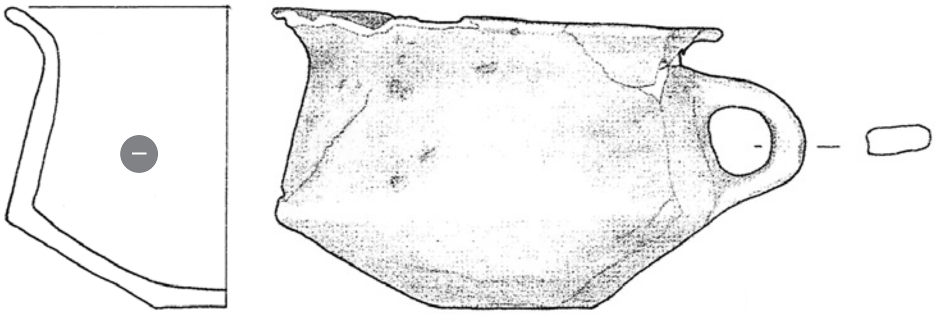

HK-Nr. 13476:4522:2b
PO-29

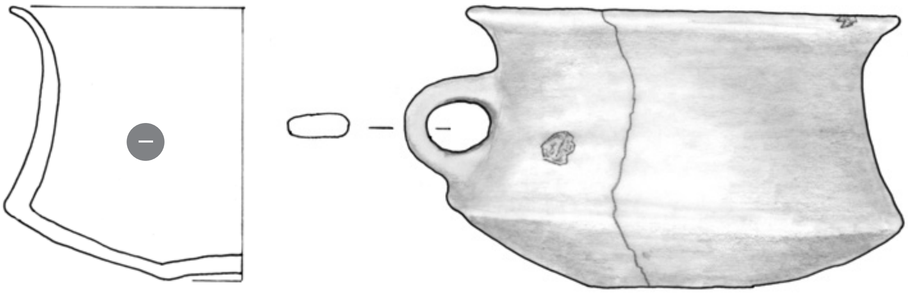

HK-Nr. 13476:4522:3b
PO-23

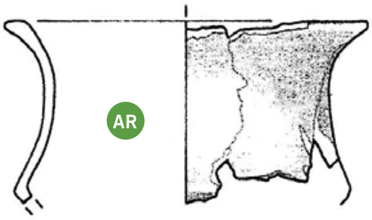

HK-Nr. 13476:4522:8a
PO-30

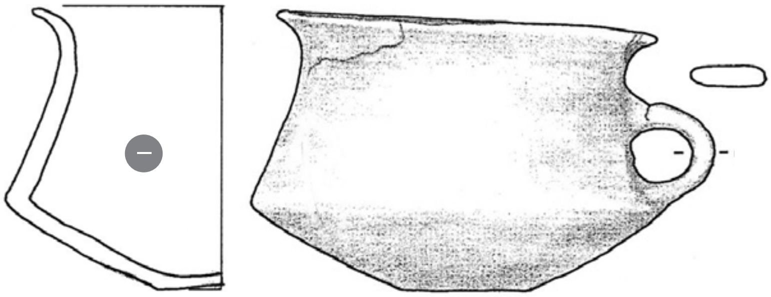

HK-Nr. 13476:4522:6a
PO-31

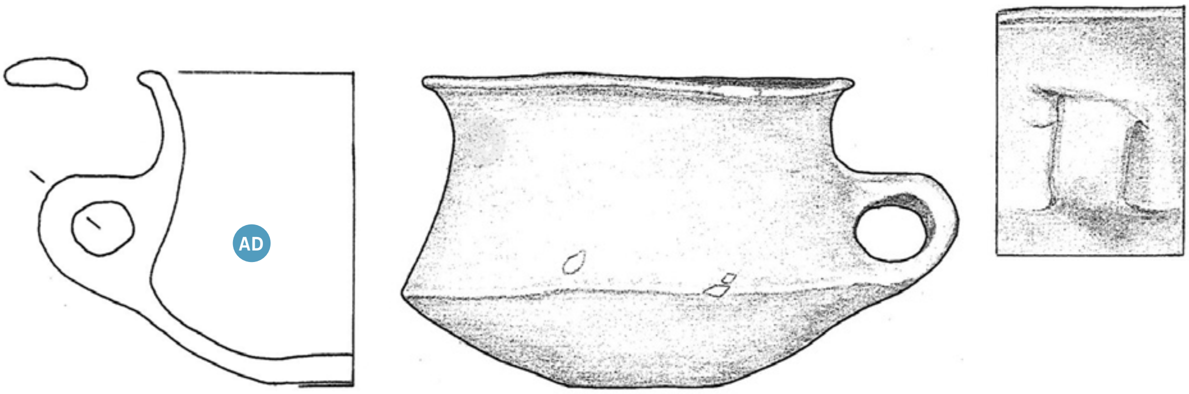

HK-Nr. 13476:4522:7a
PO-26

Pömmelte

1:2

Únětice Culture (2200–1775 BCE) – Deposit

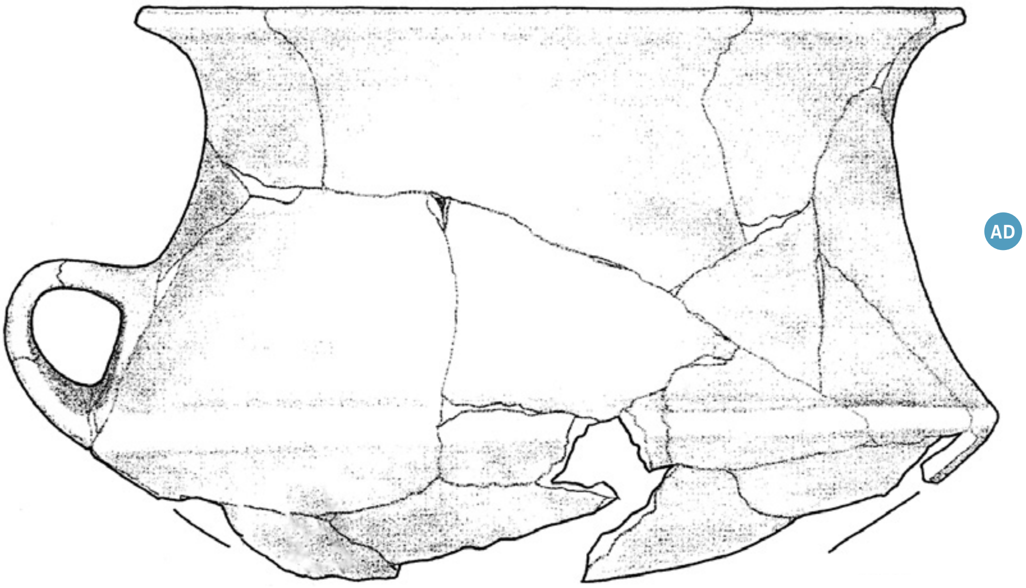

HK-Nr. 13476:4522:2a
PO-27

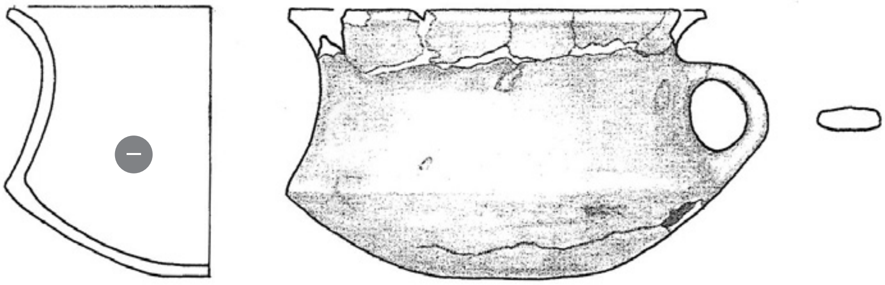

HK-Nr. 13476:4522:3a
PO-28

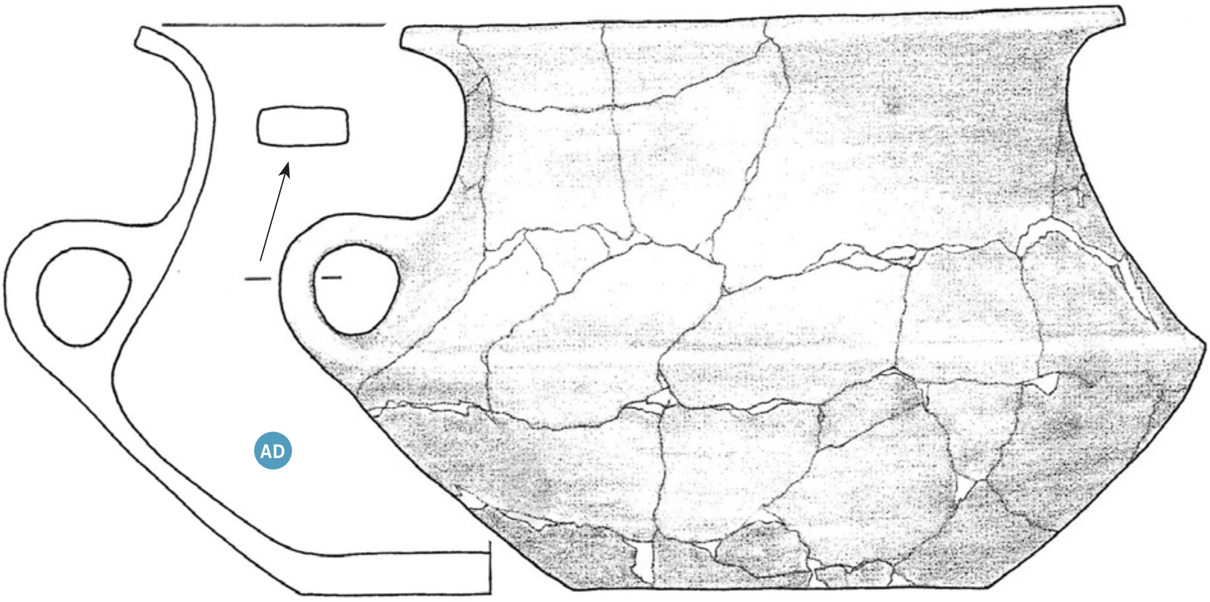

HK-Nr. 13476:4522:5a
PO-22

Pömmelte

1:2

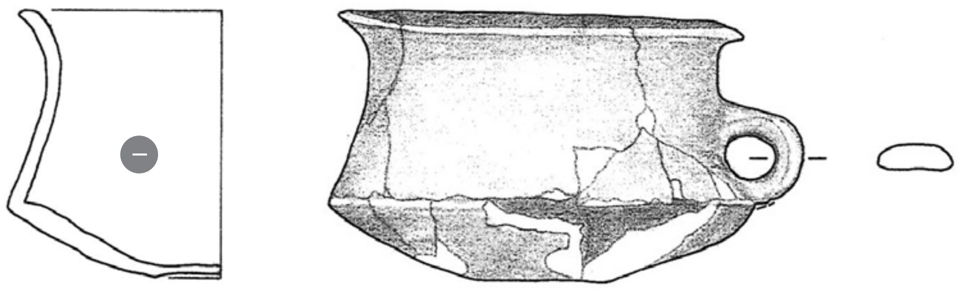

HK-Nr. 13476:4522:7b
PO-24

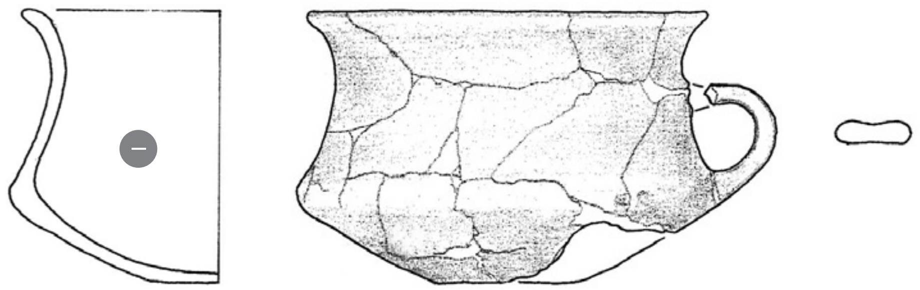

HK-Nr. 13476:4522:4a
PO-25

Pömmelte

1:2

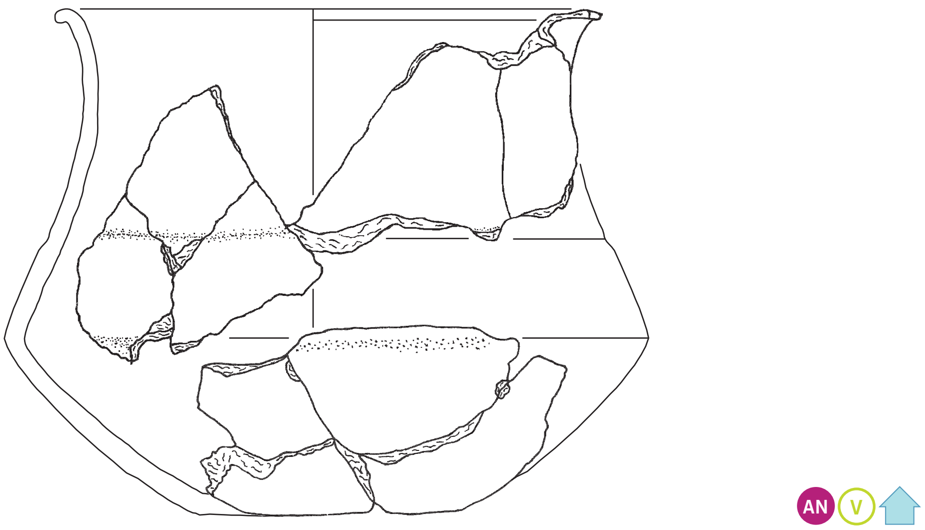

HK-Nr. 3615:20147:4a
KL-2

Kleinpaschleben

1:2

Únětice Culture (2200–1775 BCE) – Settlement

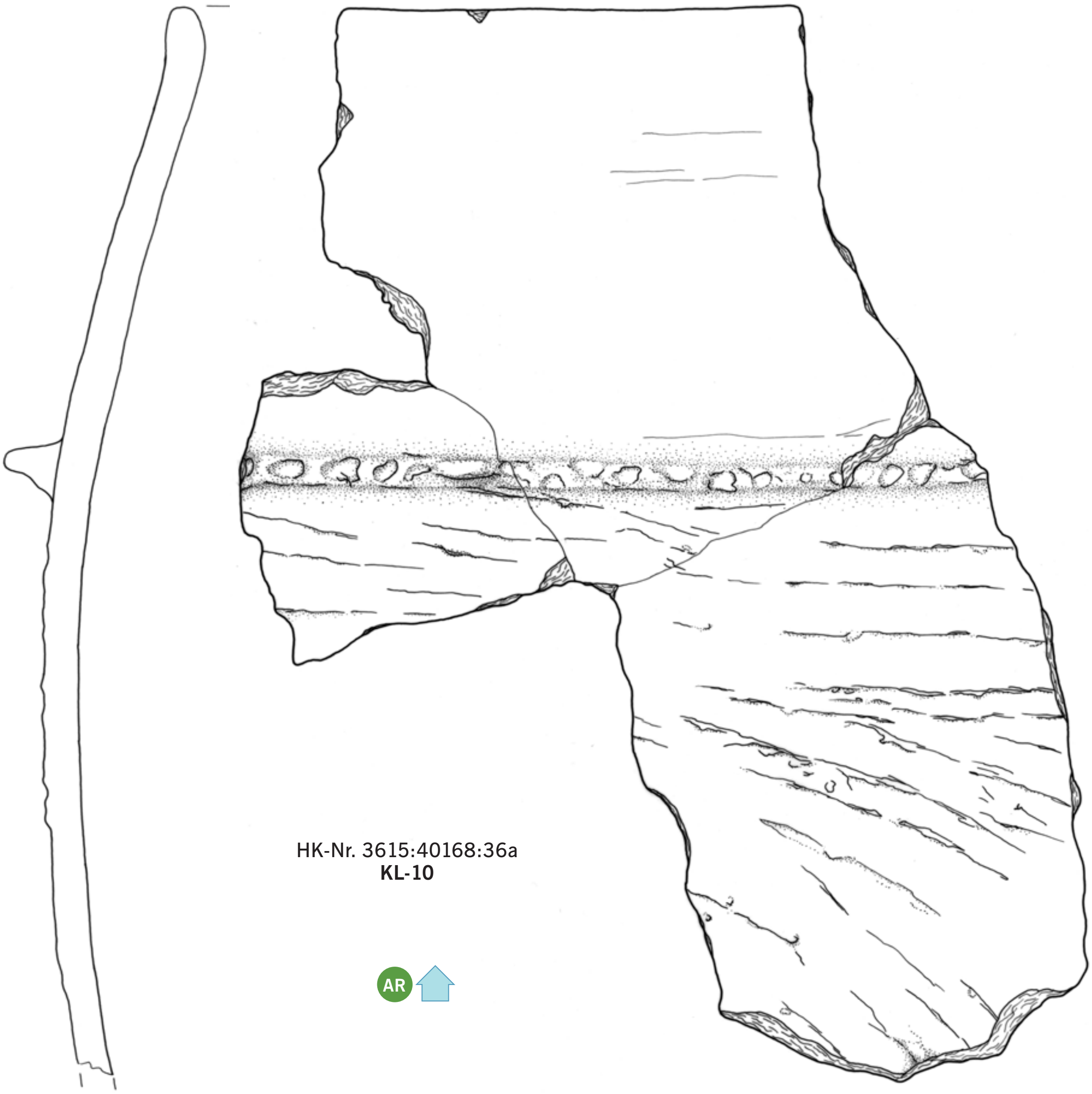

HK-Nr. 3615:40168:36a
KL-10

AR

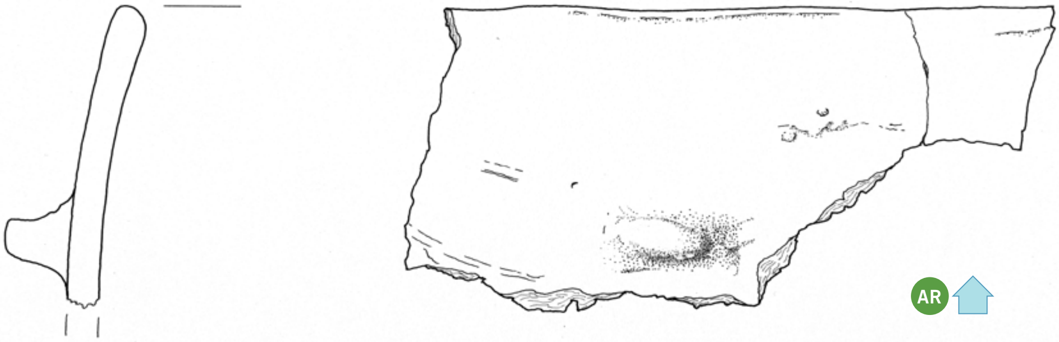

AR

HK-Nr. 3615:40168:3a
KL-4

Kleinpaschleben

1:2

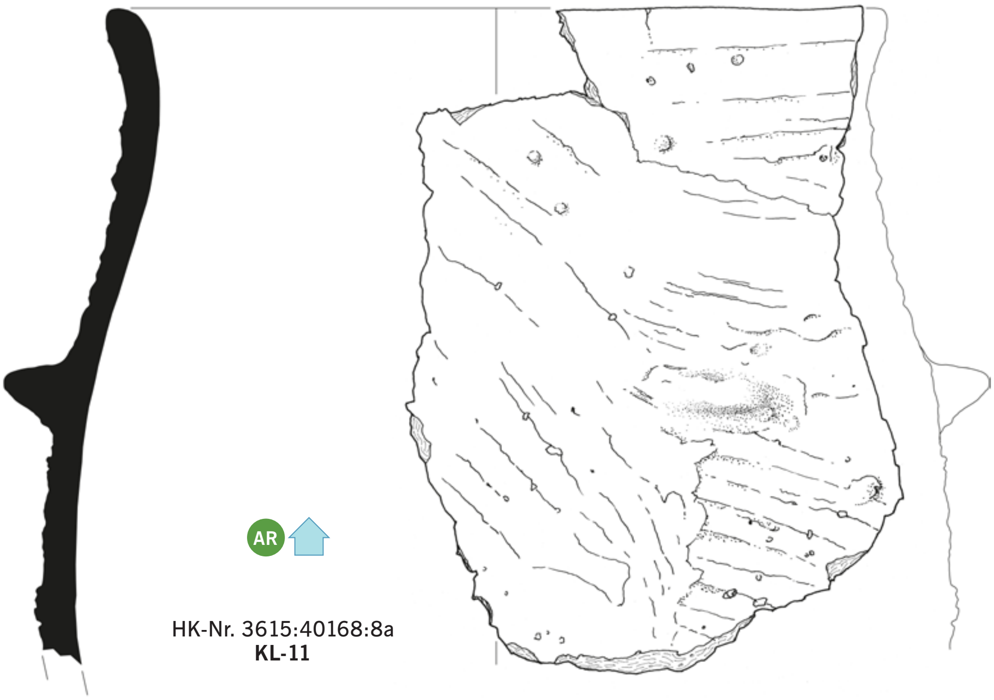

HK-Nr. 3615:40168:8a
KL-11

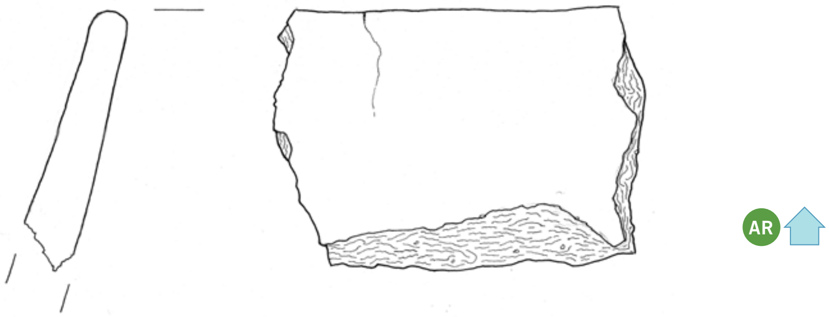

HK-Nr. 3615:40168:15a
KL-5

Kleinpaschleben

1:2

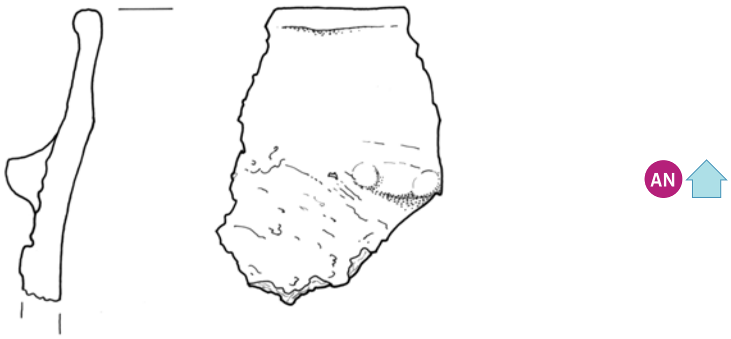

HK-Nr. 3615:30137:1a
KL-6

Kleinpaschleben

1:2

Únětice Culture (2200–1775 BCE) – Settlement

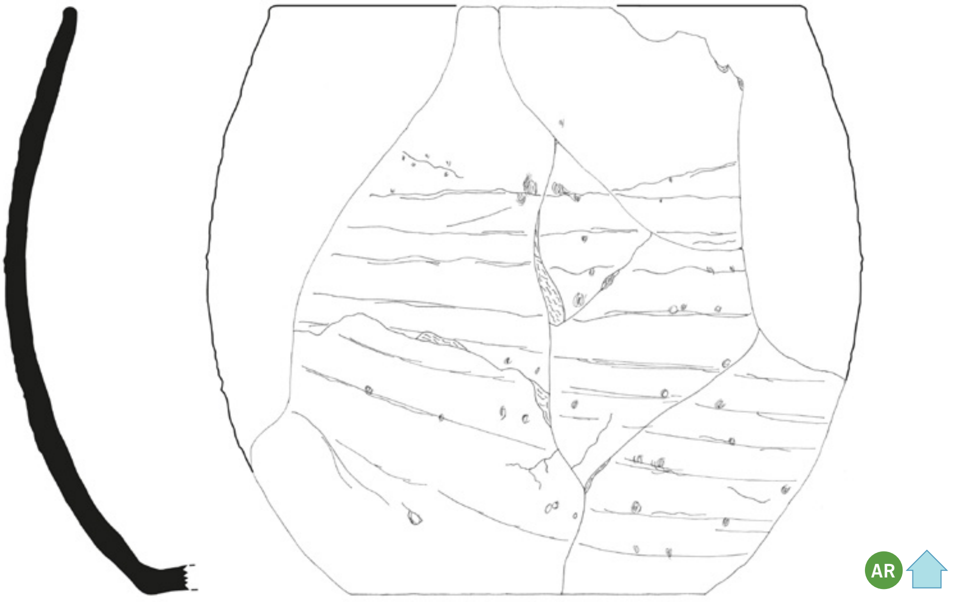

HK-Nr. 3615:40180:1a
KL-7

Kleinpaschleben

1:3

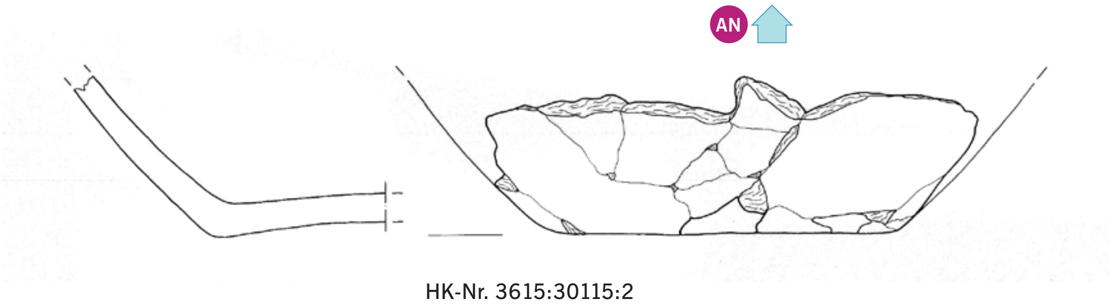

HK-Nr. 3615:30115:2
KL-1

Kleinpaschleben

1:2

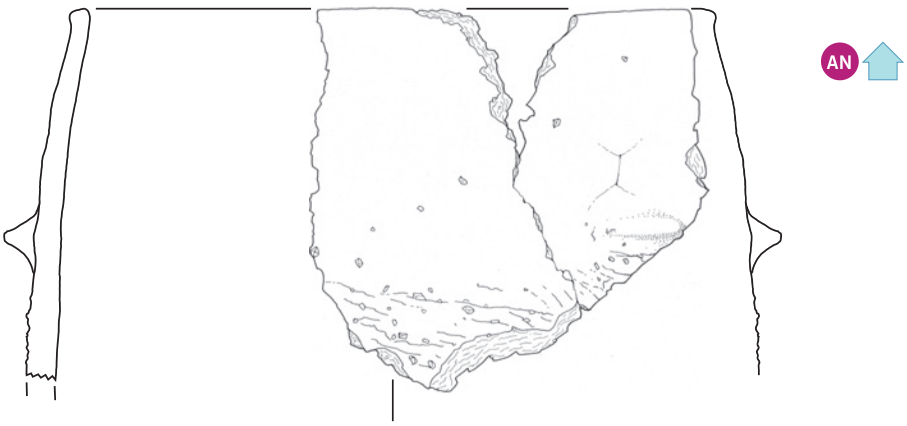

HK-Nr. 3615:20147:2a
KL-3

Kleinpaschleben

1:3

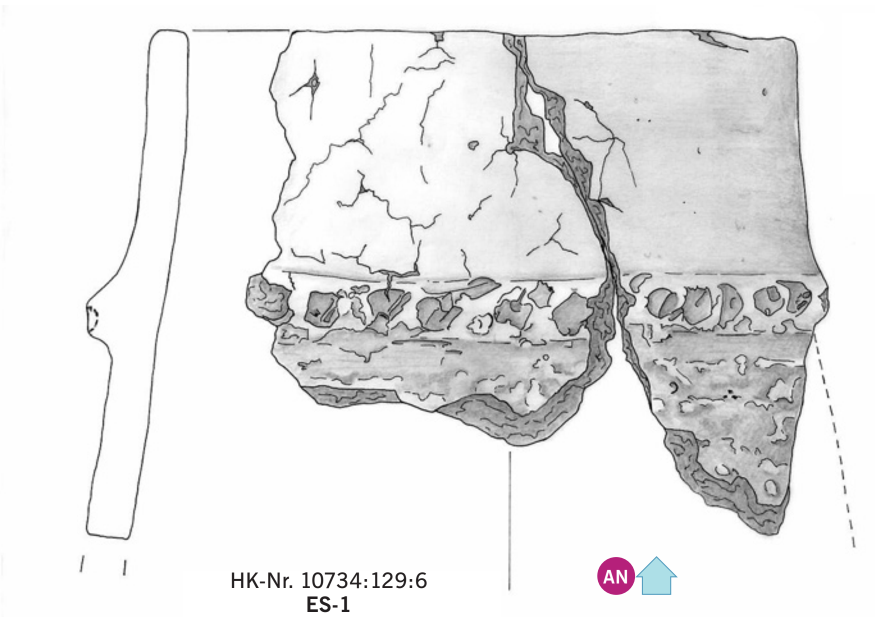

HK-Nr. 10734:129:6
ES-1

Esperstedt

1:2

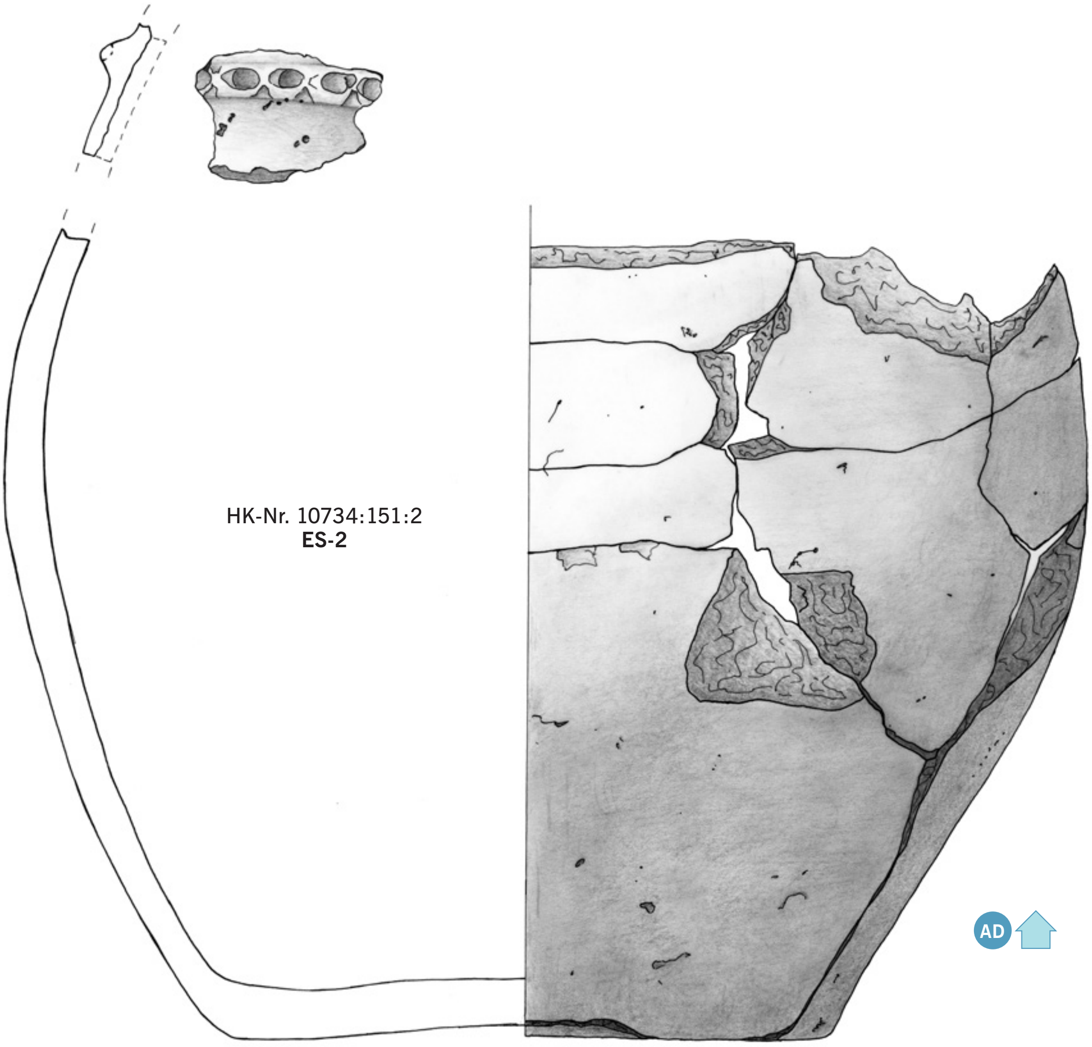

HK-Nr. 10734:151:2
ES-2

Esperstedt

1:2

Únětice Culture (2200–1775 BCE) – Settlement and Funerary

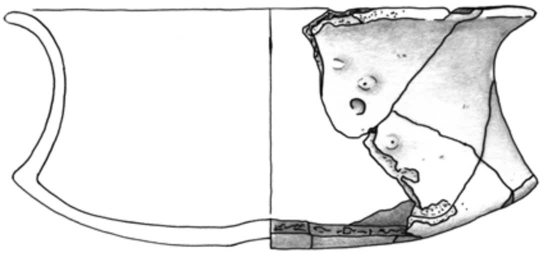

HK-Nr. 11243:330:2a
ME-5

AN

Merseburg

1:2

Adult/♀

AN

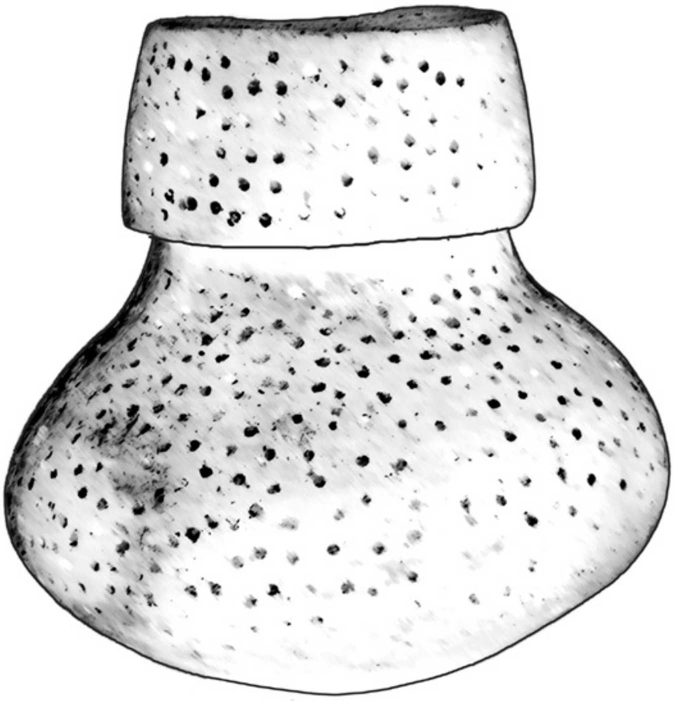

HK-Nr. 2607:290:592
OE-10
and OE-20
(vessel contents)

AN

Oechlitz

1:1

neonatus

HK-Nr. 2671:25960:14
OE-8

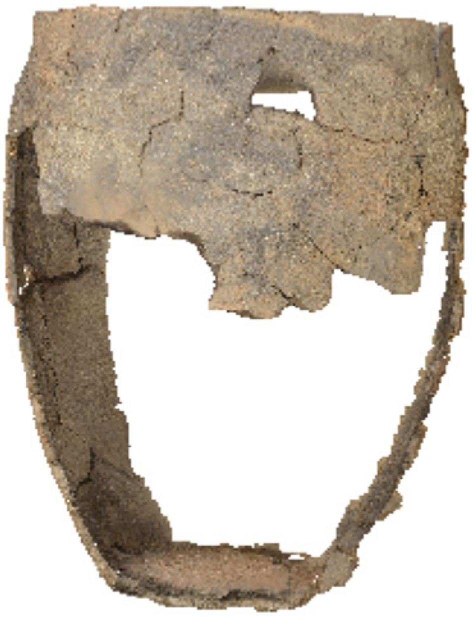

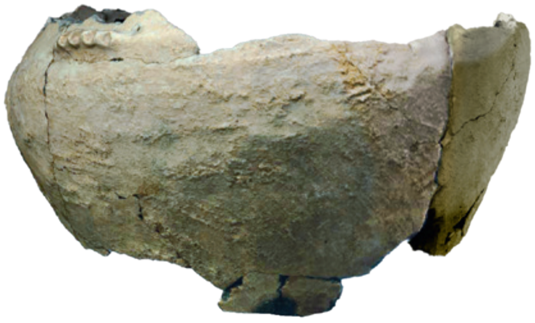

HK-Nr. 2671:25960:15
OE-9

Oechlitz

1:6

Late Bronze Age – Early Saalemündung group (1075–1000 BCE) – Funerary

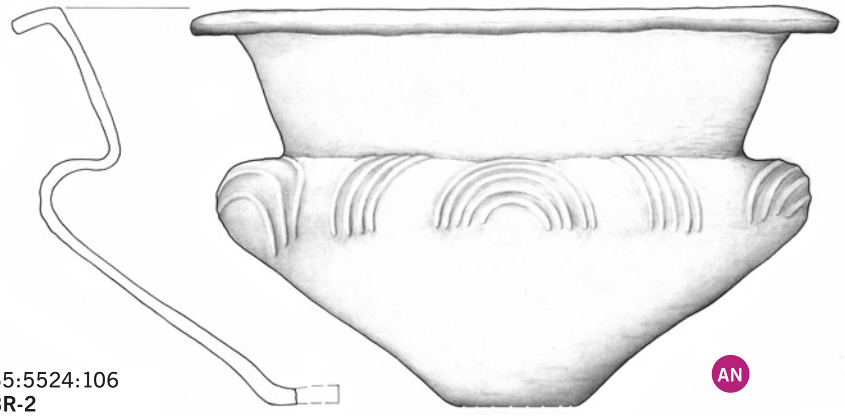

HK-Nr. 3665:5524:106
BR-2

1:6

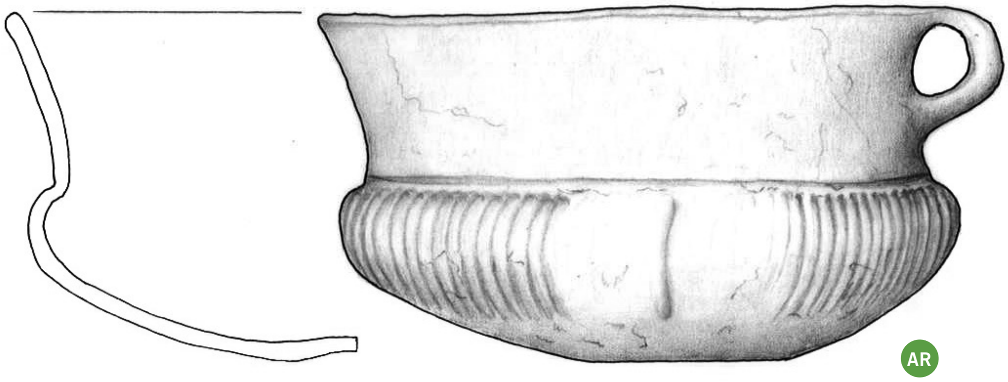

HK-Nr. 3665:5524:110a
BR-3

Bernburg

1:2
