# Supplementary material for: Pottery spilled the beans: Patterns in the processing and consumption of dietary lipids in Central Germany from the Early Neolithic to the Bronze Age
Source: PLoS One. 2024 May 16;19(5):e0301278. doi: 10.1371/journal.pone.0301278 (PMC11098342; doi:10.1371/journal.pone.0301278)
Supplement: S1 Table — (DOCX) [file pone.0301278.s002.docx]

| Period | Site | Vessel ID | Find ID* | Maximum diameter (mm) | Height (mm) | Vessel parts sampled | Extractions | TLE | P/S | Biomarkers | δ^13^C 16:0 (‰) | δ^13^C 18:0 (‰) | Δ^13^C |
| --- | --- | --- | --- | --- | --- | --- | --- | --- | --- | --- | --- | --- | --- |
| Linear Pottery | Harsleben | HA-1 | 10646:634:9 | 155 | 112 | Central | Solvent | 22 | 0.9 | FFA, TAGs | -23.7 | -27.4 | -3.6 |
|  |  | HA-2 | 10646:634:10 | - | - |  | Solvent, Acid | 762 | 0.6 | FFA | -27.1 | -27.8 | -0.6 |
|  |  | HA-3 | 10646:634:11 | - | - |  | Solvent | 76 | 0.4 | FFA, TAGs | -25.7 | -27.0 | -1.3 |
|  |  | HA-4 | 10756:38:2a  10756:38:2b | - | - | Upper and lower |  | 191 | 0.8 | FFA, TAGs | -22.6 | -23.5 | -1.0 |
|  |  | HA-5 | 10756:142:2 | - | - | Central | Solvent, Acid | 80 | 0.9 | FFA | -26.8 | -25.8 | 0.9 |
|  |  | HA-11 | 10756:27:2b | - | - |  | Solvent | 61 | 1.0 | FFA, TAGs | -24.2 | -26.4 | -2.2 |
|  |  | HA-12 | 10756:94:2c | - | - | Rim | Solvent, Acid | 98 | 0.8 | FFA | -26.2 | -27.8 | -1.6 |
| Schöningen |  | HA-6 | 10757:11001:15 | - | - | Central | Solvent | 136 | 0.7 | FFA, TAGs | -25.0 | -29.5 | -4.6 |
|  |  | HA-7 | 10757:11040:4c | >145 | >75 | Base |  | 92 | 0.7 | FFA, TAGs | -23.4 | -26.1 | -2.7 |
|  |  | HA-8 | 10757:11040:11f+d | >290 | >95 |  |  | 13 | 0.6 | FFA | -24.2 | -27.2 | -3.0 |
|  |  | HA-9 | 10757:11040:41a | 188 | - | Central |  | 127 | 0.3 | FFA, TAGs | -25.1 | -29.8 | -4.7 |
|  |  | HA-10 | 10757:11094:35 | 260 | 170 | Upper |  | 71 | 1.2 | FFA,TAGs | -25.1 | -23.7 | 1.3 |
|  |  | HA-13 | 10757:11058:2a | 260 | 190 | Central | Solvent, Acid | **3416** | 1.2 | FFA,TAGs | -25.2 | -26.4 | -1.2 |
|  |  | HA-14 | 10757:11058:1a | 280 | 200 |  | Solvent | 5 | 1.1 | FFA | -27.3 | -28.9 | -1.6 |
| Schiepzig | Libehna | LI-1 | 9495:1031:40a | 270 | 215 |  | Solvent, Acid | 17 | 1.0 | FFA | -27.3 | -26.9 | 0.4 |
|  |  | LI-2 | 9495:1031:41a | 356 | 253 |  |  | 42 | 0.8 | FFA | -27.5 | -26.7 | 0.8 |
|  |  | LI-3 | 9495:1034:35a | 285 | 262 |  | Solvent | 10 | 0.8 | FFA | -21.8 | -23.6 | -1.8 |
|  |  | LI-4 | 9495:2030:22 | 337 | 541 |  | Solvent, Acid | 209 | 0.8 | FFA | -28.0 | -26.9 | 1.1 |
|  |  | LI-5 | 9495:2030:31 | 720 | 800 |  |  | 28 | 1.0 | FFA | -28.7 | -27.5 | 1.1 |
| Baalberge | Belleben | BE-1 | BEL I-12:251; :114; :281; :125 | 213 | 207 | Upper and lower | Acid | 98 | 0.7 | FFA, MAGs | -28.0 | -30.1 | -2.1 |
|  |  | BE-2 | 14:1068 | 75 | 78 |  |  | 53 | 0.9 | FFA | -27.1 | -29.8 | -2.7 |
|  |  | BE-3 | 13:548+462; 13:426+642+486+544 | 210 | 212 | Lower |  | 63 | 0.9 | FFA, Py | -27.5 | -31.5 | -4.0 |
|  |  | BE-4 | 14:1054 | 164 | 110 | Upper and lower |  | 540 | 0.6 | FFA, Py | -28.1 | -33.1 | -5.0 |
|  |  | BE-5 | 1-95:1598;1593 | 260 | >60 |  |  | 589 | 1.1 | FFA, Py | -27.8 | -32.2 | -4.4 |
|  |  | BE-6 | 13:461 | 168 | c.170 |  |  | 752 | 0.6 | FFA, Py, Pr, C35D7O | -27.7 | -30.7 | -3.0 |
|  |  | BE-7 | 1-96:1543+1547;1-97:1578 | 284 | 220 |  |  | 18 | 2.1 | FFA | -27.5 | -31.1 | -3.6 |
|  |  | BE-8 | 14:1146 | 484 | 445 | Unknown |  | 23 | 2.4 | FFA | -27.4 | -30.4 | -3.0 |
|  |  | BE-9 | 14:1054+887+1068; 14:1110+1132+1068 | 330 | c.250 | Upper and lower |  | 107 | 0.9 | FFA, **APAA** | -27.5 | -33.2 | -5.7 |
|  |  | BE-10 | 10:17 | - | - |  |  | 147 | 0.7 | FFA, Py, Pr | -27.5 | -33.3 | -5.8 |
|  |  | BE-11 | 10:17 | Small | Small |  |  | 148 | 0.5 | FFA, Py, Pr | -27.6 | -34.0 | -6.4 |
|  | Queis | QU-40 | 2002:2481a | 93 | 108 |  |  | 773 | 1.1 | FFA, Py, DHA | -27.3 | -33.9 | -6.6 |
|  |  | QU-41 | 2002:2481b | 207 | 225 |  |  | 17 | 1.0 | FFA, DHA | - | - | - |
| Baalberge | Queis | QU-42 | 2002:2481c | 117 | 75 | Upper and lower | Acid | 417 | 0.7 | FFA, Py, C35D7O, DHA, 7ODA | -27.9 | -33.0 | -5.1 |
|  |  | QU-43 | 2002:2482a | 177 | 186 |  |  | 38 | 1.2 | FFA | - | - | - |
|  |  | QU-44 | 2002:2482b | 219 | 126 |  |  | 611 | 1.3 | FFA, Py, C35D7O, DHA, 7ODA, 7ODDA | -28.5 | -34.6 | -6.1 |
|  |  | QU-45 | 2002:2482c | 330 | 342 |  |  | 48 | 1.2 | FFA, DHA, 7ODA | -27.0 | -29.4 | -2.4 |
|  |  | QU-46 | 2002:2483a | 165 | 105 |  |  | 331 | 1.1 | FFA, Py, C35D7O, DHA, 7ODA | -28.6 | -35.5 | -6.9 |
|  |  | QU-47 | 2002:2483b | 180 | 216 |  |  | 33 | 0.9 | FFA, DHA | -25.6 | -30.7 | -5.1 |
|  |  | QU-48 | 2002:2484a | 240 | 231 |  |  | 26 | 1.6 | FFA | - | - | - |
|  |  | QU-49 | 2002:2484b | 153 | 90 |  |  | 406 | 1.2 | FFA, Py, C35D7O, DHA, 7ODA, 7ODDA | -27.2 | -33.1 | -5.9 |
|  | Pömmelte | PO-13 | 12820:2574:1a | 135 | 164 | Lower |  | 1.0 | 1.0 | FFA | - | - | - |
|  |  | PO-14 | 13476:2970:1a | 119 | 158 |  |  | 5 | 1.1 | FFA | - | - | - |
|  |  | PO-15 | 13476:2970:2a | 107 | 67 |  |  | 14 | 1.1 | FFA, Py, C35D7O, DHA(tr) | -27.0 | -31.9 | -4.9 |
|  |  | PO-19 | 13476:5501:2b | 129 | 65 | Upper |  | 174 | 1.2 | FFA, Py, Pr, C35D7O, DHA, 7ODA | -27.3 | -32.5 | -5.2 |
|  |  | PO-20 | 13476:5501:2a | 151 | 187 | Lower |  | 2.0 | 1.1 | FFA | - | - | - |
| Corded Ware | Bernburg | BR-1 | 3125:1581:7 | 85 | 100 | Central | Solvent | 9 | 0.9 | FFA | -31.0 | -32.5 | -1.5 |
|  | Oechlitz | OE-1 | 2671:25355:26 | 112 | 89 |  | Solvent, Acid | 64 | 1.1 | FFA | -27.2 | -27.3 | -0.2 |
|  |  | OE-2 | 2671:25355:27 | 223 | >124 |  |  | 94 | 0.9 | FFA | -27.4 | -27.2 | 0.2 |
|  |  | OE-3 | 2671:25355:28 | 115 | 103 |  |  | 17 | 1.6 | FFA | -28.3 | -28.1 | 0.2 |
|  |  | OE-4 | 2671:25645:104 | 131 | 187 |  |  | 373 | 0.9 | FFA | -28.4 | -32.6 | -4.2 |
|  |  | OE-5 | 2671:25645:70 | 150 | 143 | Upper | Solvent | 110 | 0.8 | FFA, MAGs, DAGs, TAGs | -28.7 | -30.7 | -2.0 |
|  |  | OE-6 | 2671:25645:75 | 150 | 143 | Lower | Solvent, Acid | **9469** | 0.9 | FFA, MAGs, DAGs, TAGs | -29.2 | -31.4 | -2.2 |
|  |  | OE-7 | 2671:25815:38 | ND | ND | Central |  | 126 | 3.1 | FFA | -26.5 | -25.0 | 1.4 |
|  | Profen | PR-1 | 2510:5043:23 | 68 | 65 |  |  | 222 | 1.0 | FFA, TAGs | -25.4 | -25.7 | -0.3 |
|  |  | PR-2 | 2510:5043:24 | 80 | 69 |  |  | 92 | 1.0 | FFA | -30.1 | -30.4 | -0.3 |
|  |  | PR-3 | 2510:5043:25 | 110 | 155 | Upper |  | 40 | 1.2 | FFA | -28.9 | -29.4 | -0.5 |
|  |  | PR-4 | 2510:5043:27 | 247 | 195 | Central |  | 553 | 1.2 | FFA | -27.7 | -27.0 | 0.7 |
|  |  | PR-5 | 2510:5068:7 | 153 | 50 |  |  | 578 | 1.1 | FFA | -25.5 | -26.5 | -1.1 |
|  |  | PR-6 | 2510:5068:8 | 102 | 136 |  |  | **5006** | 0.5 | FFA, MAGs, DAGs, TAGs | -27.5 | -30.3 | -2.7 |
|  |  | PR-9 | 2510:7616:35 | 68 | 65 |  |  | **1740** | 1.1 | FFA, | -27.9 | -26.9 | 1.0 |
|  |  | PR-10 | 2510:7616:43 | 84 | 87 |  | Solvent, Acid | **2245** | 0.9 | FFA, Chol, C35D7O, MAGs, DAGs | -28.2 | -32.6 | -4.4 |
| Corded Ware | Profen | PR-11 | 2510:7618:16 | 214 | 214 | Central | Solvent | 6 | 0.9 | FFA, Chol, MAGs, DAGs | -28.8 | -28.2 | 0.7 |
|  |  | PR-12 | 2510:7618:17 | 174 | 200 |  | Solvent, Acid | 599 | 0.9 | FFA | -29.4 | -33.7 | -4.3 |
|  |  | PR-13 | 2510:7618:18 | 85 | 85 |  | Solvent | 132 | 1.2 | FFA, MAGs, DAGs, C35D7O, **Campesterol, β-Sitosterol** | -28.5 | -31.6 | -3.1 |
|  |  | PR-14 | 2510:7625:16a | c.330 | >143 | Upper | Solvent, Acid | **2152** | 0.8 | FFA | -30.7 | -30.2 | 0.6 |
|  |  | PR-15 | 2510:7625:17a | c.330 | >143 | Lower |  | 127 | 1.0 | FFA | -28.5 | -28.3 | 0.1 |
|  |  | PR-16 | 2510:7868:33a | 195 | 155 | Central |  | **1595** | 0.8 | FFA | -31.0 | -30.5 | 0.5 |
|  |  | PR-17 | 2510:7868:34a | 79 | 100 |  |  | **2760** | 0.8 | FFA, MAGs, DAGs, TAGs(tr) | -29.7 | -31.4 | -1.7 |
|  |  | PR-18 | 2510:8821:11 | 198 | 174 |  |  | **1162** | 0.8 | FFA | -28.1 | -27.2 | 0.9 |
|  | Wennungen | WE-1 | 2365:5351:23 | c.250 | c.250 |  |  | 291 | 0.9 | FFA | -30.6 | -32.0 | -1.5 |
|  |  | WE-2 | 2365:5351:49a | 76 | 90 |  |  | **7695** | 0.9 | FFA | -28.7 | -33.2 | -4.5 |
|  |  | WE-3 | 2365:5351:50a | 108 | 133 |  |  | **1446** | 1.1 | FFA, Py. 7ODA, **APAA** | -28.7 | -32.1 | -3.5 |
|  | Pömmelte | PO-32 | 13476:5638:1a | 60 | 78 | Lower | Acid | 29 | 0.8 | FFA, Py | -25.9 | -30.7 | -4.8 |
| Bell Beaker | Profen | PR-7 | 2510:5427:21 | 130 | 100 | Central | Solvent, Acid | 898 | 1.1 | FFA | -28.7 | -30.0 | -1.3 |
|  |  | PR-8 | 2510:5427:22 | 98 | 74 |  |  | 232 | 1.5 | FFA, Contaminated | - | - | - |
|  | Pömmelte | PO-17 | 13476:4200:2a | 146 | 100 | Upper | Acid | **1185** | 0.9 | FFA, Py, Pr, C35D7O | -27.3 | -31.6 | -4.3 |
|  |  | PO-18 | 13476:4207:1a | 167 | 113 | Lower |  | 129 | 1.0 | FFA, Py, C35D7O, **LCK**, APAA(tr) | -26.6 | -31.5 | -4.9 |
|  |  | PO-21 | 13476:5643:4a | 132 | 102 |  |  | 166 | 0.9 | FFA | -27.6 | -33.2 | -5.6 |
|  |  | PO-34 | 12820:1567:1 | 159 | 84 |  |  | 781 | 1.3 | FFA, Py, **LCK, APAA, ACPAA** | -25.9 | -31.1 | -5.2 |
|  |  | PO-35 | 12820:1454:1a | 123 | 89 | Upper |  | 201 | 0.5 | FFA, Py | -27.1 | -31.7 | -4.6 |
|  |  | PO-36 | 12820:1886:2a | 104 | 76 | Lower |  | 133 | 0.5 | FFA, Py, Pr | -26.1 | -31.8 | -5.7 |
|  |  | PO-37 | 12820:1750:2a | 154 | 116 |  |  | 175 | 1.3 | FFA, Py, Pr | -26.8 | -32.0 | -5.2 |
|  |  | PO-38 | 12820:1742:1a | 112 | 101 |  |  | 345 | 0.8 | FFA, Py, Pr, C35D7O, **APAAC18, ACPAA, oxoVLCFA** | -24.2 | -27.8 | -3.6 |
| Únětice | Esperstedt | ES-1 | 10734:129:6 | >220 | >130 | Upper |  | **1193** | 0.8 | FFA | -32.2 | -31.7 | 0.5 |
|  |  | ES-2 | 10734:151:2 | 285 | >210 | Central |  | 822 | 1.1 | FFA, VLCFA | -29.2 | -33.7 | -4.5 |
|  | Kleinpaschleben | KL-1 | 3615:30115:2 | Large | Large | Base | Solvent | 122 | 1.1 | FFA | - | - | - |
|  |  | KL-2 | 3615:20147:4a | 170 | 132 | Central |  | 465 | 0.9 | FFA, DHA, **Pimaric**, 2DHA, 7ODA | -28.0 | -27.4 | 0.6 |
|  |  | KL-3 | 3615:20147:2a | C.300 | C.320 | Upper |  | 470 | 1.9 | FFA | -30.0 | -29.0 | 1.1 |
|  |  | KL-4 | 3615:40168:3a | 270 | - |  |  | **1288** | 0.7 | FFA | -29.5 | -31.0 | -1.5 |
| Únětice | Kleinpaschleben | KL-5 | 3615:40168:15a | 270 | - |  |  | 166 | 1.0 | FFA | -28.0 | -28.9 | -0.8 |
|  |  | KL-6 | 3615:30137:1a | 135 | - |  | Solvent, Acid | 311 | 0.8 | FFA | -30.9 | -30.5 | 0.3 |
|  |  | KL-7 | 3615:40180:1a | 263 | 235 | Central |  | **3626** | 0.7 | FFA, Py. **LCK, APAA, ACPAA** | -29.3 | -31.3 | -2.0 |
|  |  | KL-10 | 3615:40168:36a | Large | >300 |  | Solvent | 5 | 1.5 | FFA, MAGs, DAGs, DAGs(tr) | -29.5 | -29.9 | -0.4 |
|  |  | KL-11 | 3615:40168:8a | 240 | >180 |  | Solvent, Acid | 223 | 1.1 | FFA | -30.6 | -30.8 | -0.2 |
|  | Helmsdorf | HE-1 | Tomb | - | - |  | Solvent | 27 | NC | Contamination | - | - | - |
|  |  | HE-2 | Tomb | - | - |  |  | 628 | 2.3 | Contamination | - | - | - |
|  |  | HE-3 | Tomb | - | - |  |  | NC | NC | Contamination | - | - | - |
|  | Leubingen | LE1 | Tomb | - | - | Rim |  | NC | NC | Contamination | - | - | - |
|  |  | LE2 | Tomb | - | - | Central |  | NC | NC | Contamination | - | - | - |
|  |  | LE3 | Tomb | - | - |  |  | NC | NC | Contamination | - | - | - |
|  | Merseburg | ME-1 | 11243:105:1 | - | - |  | Acid | 328 | 0.8 | FFA | -30.9 | -31.1 | -0.2 |
|  |  | ME-2 | 11243:197:3c | - | - |  |  | **1112** | 0.8 | FFA | -30.4 | -30.6 | -0.2 |
|  |  | ME-3 | 11243:197:5b | - | - |  |  | 279 | 0.8 | FFA | -31.6 | -32.6 | -1.0 |
|  |  | ME-4 | 11243:254:1a | - | - |  |  | 184 | 0.8 | FFA | -31.0 | -31.0 | 0.0 |
|  |  | ME-5 | 11243:330:2a | 139 | 63 |  |  | 12 | 0.9 | FFA | -26.0 | -25.1 | 1.0 |
|  | Oechlitz | OE-8 | 2671:25960:14 | 365 | 470 |  | Solvent, Acid | 164 | 0.8 | FFA | -27.6 | -27.3 | 0.3 |
|  |  | OE-9 | 2671:25960:15 | >250 | - |  |  | 113 | 1.6 | FFA | -27.1 | -26.4 | 0.7 |
|  |  | OE-10 | 2607:290:592 | 92 | 75 | Rim |  | >5 | NC | FFA | -24.2 | -24.3 | -0.1 |
|  |  | OE-20 | 2607:290:587 | - | - | Visible residue |  | >5 | NC | FFA | -25.9 | -25.7 | 0.3 |
|  | Pömmelte | PO-12 | 12820:2372:1a | 375 | 167 | Base | Acid | 1.0 | 1.2 | FFA | - | - | - |
|  |  | PO-16 | 13476:3298:1 | >78 | >37 |  |  | 48 | 0.7 | FFA | -27.8 | -29.7 | -1.9 |
|  |  | PO-22 | 13476:4522:5a - V1 | 226 | 158 | Lower |  | 9 | 0.9 | FFA | -27.3 | -33.1 | -5.8 |
|  |  | PO-23 | 13476:4522:3b - V2 | 120 | 75 |  |  | 1.0 | 1.0 | FFA | - | - | - |
|  |  | PO-24 | 13476:4522:7b - V 3 | 113 | 72 | Lower |  | 1.0 | 1.1 | FFA | - | - | - |
|  |  | PO-25 | 13476:4522:4a - V4 | 109 | 72 | Upper |  | 0.0 | 0.7 | FFA | - | - | - |
|  |  | PO-26 | 13476:4522:7a - V5 | 123 | 84 |  | Acid | 55 | 1.2 | FFA | -27.3 | -31.7 | -4.4 |
|  |  | PO-27 | 13476:4522:2a - V6 | 244 | 154 |  |  | 177 | 0.9 | FFA, Py, Pr, **TMTD**, C35D7O | -27.0 | -31.2 | -4.2 |
|  |  | PO-28 | 13476:4522:3a - V7 | 114 | 72 |  |  | 3 | 1.1 | FFA | - | - | - |
|  |  | PO-29 | 13476:4522:2b - V8 | 126 | 85 |  |  | 1.0 | 1.0 | FFA | - | - | - |
|  |  | PO-30 | 13476:4522:8a - V9 | 96 | 49 |  |  | 14 | 0.6 | FFA, Py, Pr (tr) | -29.0 | -31.5 | -2.5 |
|  |  | PO-31 | 13476:4522:6a - V10 | 109 | 75 | Lower |  | 3 | 0.9 | FFA | - | - | - |
|  |  | PO-33 | 13476:2974:1a | 418 | 489 |  |  | 0 | NC | FFA | - | - | - |
|  |  | PO-39 | 13476:2702:1a | 89 | 64 | Upper |  | 7 | 0.7 | FFA | - | - | - |
| Saalemündung | Bernburg | BR-2 | 3665:5524:106 | 518 | 318 | Rim, upper and lower | Solvent, Acid | 7529 | 0.8 | Contamination in rim and upper body. | -31.1 | -30.7 | 0.5 |
|  |  | BR-3 | 3665:5524:110a | 333 | 186 | Rim | Solvent | 12 | 0.9 | FFA | -25.5 | -25.8 | -0.3 |
| Reference samples | Elsteraue | EL-1 | 2373:4262:1025 | - | - |  |  | 18 | 0.9 | FFA | -30.4 | -30.8 | -0.4 |
|  | Eisleben | EI-1 |  | - | - |  |  | 11 | 1.1 | FFA | -31.2 | -30.2 | 1.0 |
|  | Preußlitz | PE-1 | 171:3055:31089 | - | - |  | Acid | 3034 | 0.7 | FFA | -26.2 | -27.5 | -1.3 |

**Table 1: FFA:** free saturated and monounsaturated fatty acids 12 to 20 carbons long dominated by C16:0 and C18:0**. TLE:** total lipid extract expressed in μg·g^-1^, vessels yielding very high concentrations of lipids
(>1 mg g^-1^) are marked in bold. **P/S:** Palmitic to Stearic acid ratio. **MAGs**: Monoacylglycerols. **DAGs**: Diacylglycerols, **TAGs**: Triacylglycerols. **Py**: Phytanic acid. **Pr**: Pristanic acid. **Chol**: Cholesterol methyl ester. **C35D7O**: Cholesta-3,5-dien-7-one. **APAA**: ω-(o-alkylphenyl)alkanoic acids 18 carbons long, **ACPAA**: ω-(2-alkylcyclopentyl)alkanoic acids, **oxoVLCFA**s: very long chained oxo fatty acids, **TMTD**: 4,8,12-Trimethyltridecanoic acid. **LCK**: Long chained ketones (K31, K33, K35). **DHA**: Dehydroabietic acid methyl ester, **2DHA**: didehydroabietic acid, **7ODA**: 7-oxo-dehydroabietic acid. **7ODDA**: 7-oxo-dehydrodehydroabietic acid. **tr**: molecule detected in trace amounts. **NC**: Not calculated. *Full find codes. Shorthened codes appear in S1 for reference.
