# Supplementary material for: Pottery spilled the beans: Patterns in the processing and consumption of dietary lipids in Central Germany from the Early Neolithic to the Bronze Age
Source: PLoS One. 2024 May 16;19(5):e0301278. doi: 10.1371/journal.pone.0301278 (PMC11098342; doi:10.1371/journal.pone.0301278)
Supplement: S1 File — (DOCX) [file pone.0301278.s003.docx]

**Supplementary materials 3: analytical parameters**

**3.1 Extraction procedures:**

*Dichloromethane/methanol* (solvent extraction)

Samples were processed with a procedure adapted from Evershed et al. [1]. An *n*-tetratriacontane standard (40 μL of a 548 ng μL^-1^ solution in isooctane) was added to the sample for quantification of target analytes and to appraise their analytical recoveries. The pulverised sherds were solvent extracted twice with 10 mL of DCM/MeOH (2:1 v/v) for 15 min. with an ultrasonic bath. Following separation from the ground potsherd, the solvent extract was evaporated under a gentle stream of nitrogen under mild heat to obtain the total lipid extract, which was treated with N,O-bis(trimethylsilyl)trifluoroacetamide (BSTFA) at 70ºC for 1h prior to analysis by gas chromatography (GC).

*Acidified methanol*

After spiking the samples with the same internal standard (n-C34, 40 μL of a 548 ng μL^-1^ solution in isooctane), 4 mL of MeOH were added to the pulverised sherds before ultrasonication for 15 min. Subsequently, the samples were acidified with concentrated sulphuric acid (800 μl). The acidified solution was heated on a hot plate for 4 h at 70ºC and then cooled to room temperature. The solution was centrifuged (3000 r.p.m. for 5 min) and the supernatant was transferred to a clean glass tube to extract the target lipids with n-hexane (3 times x 2 ml) through a pipette packed with potassium carbonate. The combined *n*-hexane extracts were evaporated to dryness under a gentle stream of nitrogen under mild heat and re-dissolved in isooctane in a vial to be directly analysed by GC using standard protocols. In selected cases, samples were derivatised with BSTFA+1%TMCS for 1h at 70ºC to detect additional compounds.

The acidified methanol protocol delivered a higher lipid yield in comparison to the use of DCM/MeOH with simultaneous production of methyl esters of fatty acids (FAME), (fig. 2) (Correa-Ascencio and Evershed 2014). However, it also leads to the acid hydrolysis of complex moieties (such as acylglycerols, wax esters), which complicates their identification in the potsherds organic residues.


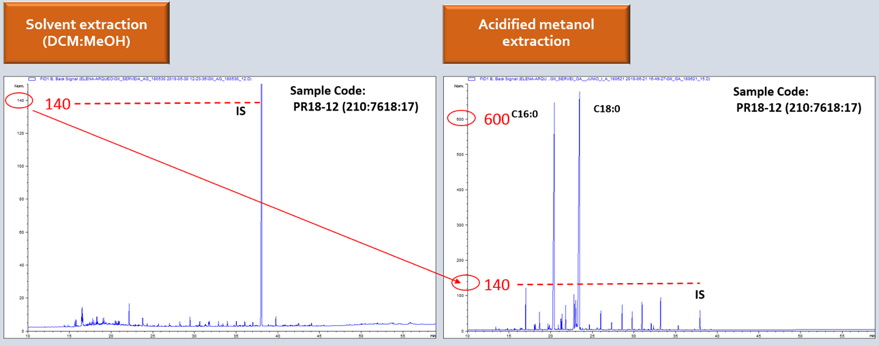


Partial GC-MS (total ion current) mass chromatogram of sample PR-12. Peak identities: Cx:0 correspond to long-chain fatty acids with nº carbon atoms. IS: Internal Standard (AL34: n-C34). The same sample was analysed with both extraction protocols. The figure on the left shows the results obtained with DCM/MeOH solvent extraction. The recovery was very low and no fatty acids were identified. The figure on the right shows the results obtained with the acidified MeOH protocol. The recovery was five times higher than with DCM/MeOH.

**3.2 Instrumental parameters:**

GC-FID parameters

GC analyses were performed on an Agilent 7890A Series GC system fitted with a flame ionization detector and using H as a carrier gas. Sample aliquots (1 μL) were introduced by on-column injection into a HP1 capillary column (60 m length, 0.25 mm internal diameter, and 0.25 μm film thickness). The temperature programme was as follows: the initial temperature was held at 70 °C for 5 min, then it increased at 10 °C min^-1^ to 320 °C and held at that temperature for 20 min. Peaks were identified by their relative retention times and by comparison with retention times of an external standard.

GC-MS parameters:

This analysis was carried out on an Agilent 7820A Series GC coupled to an Agilent 5975C mass spectrometric detector using He as a carrier gas. The GC was fitted with a split/splitless injector in splitless mode and an Agilent DB5-MS capillary column (15m length, 0.25 mm internal diameter and 0.25 μm film thickness). The MS was run in electron impact ionisation mode (70 eV), and scanning a mass range from 50 to 800 m/z. The GC oven temperature was held at 50 °C for 2 min, increased to 340 °C at a rate of 10 °C min^-1^, and held at 340 °C for 29 min.

GC-C-IRMS parameters:

Analyses were carried out using a Thermo Ultra GC connected to a Thermo MAT253 IRMS via a platinum/copper/nickel oxide combustion furnace. The GC was fitted with a (5% phenyl)-methylpolysiloxane capillary column (60 m length, 0.25 mm internal diameter, and 0.25μm film thickness). The GC oven was programmed as follows: 80 °C isothermal for 1 min, 80 to 120 °C at 30 °C min^-1^, 120 to 320 °C at 6 °C min^-1^, and held at 320 °C for 21 minutes. The combustion furnace was heated to 940 °C. Post combustion water was removed by a water permeable nafion membrane.

**3.3 Statistical treatment of the TLE values:**

The amount of lipids recovered from a pottery vessel, the Total Lipid Extract (TLE), is expressed in microgrammes of fat per gramme of pottery (μg g^-1^) and it is used to discriminate between positive and negative samples. Typically, 5 μg g^-1^ are used as the threshold, more being interpreted as positive and less being interpreted as negative. The two types of extractions (DCM/MeOH and Acidified Methanol) used to prepare the samples are known to yield different Total Lipid Extracts, with the Acidified Methanol extraction being almost always superior [2,3]. This makes it hard to compare TLEs between extractions.


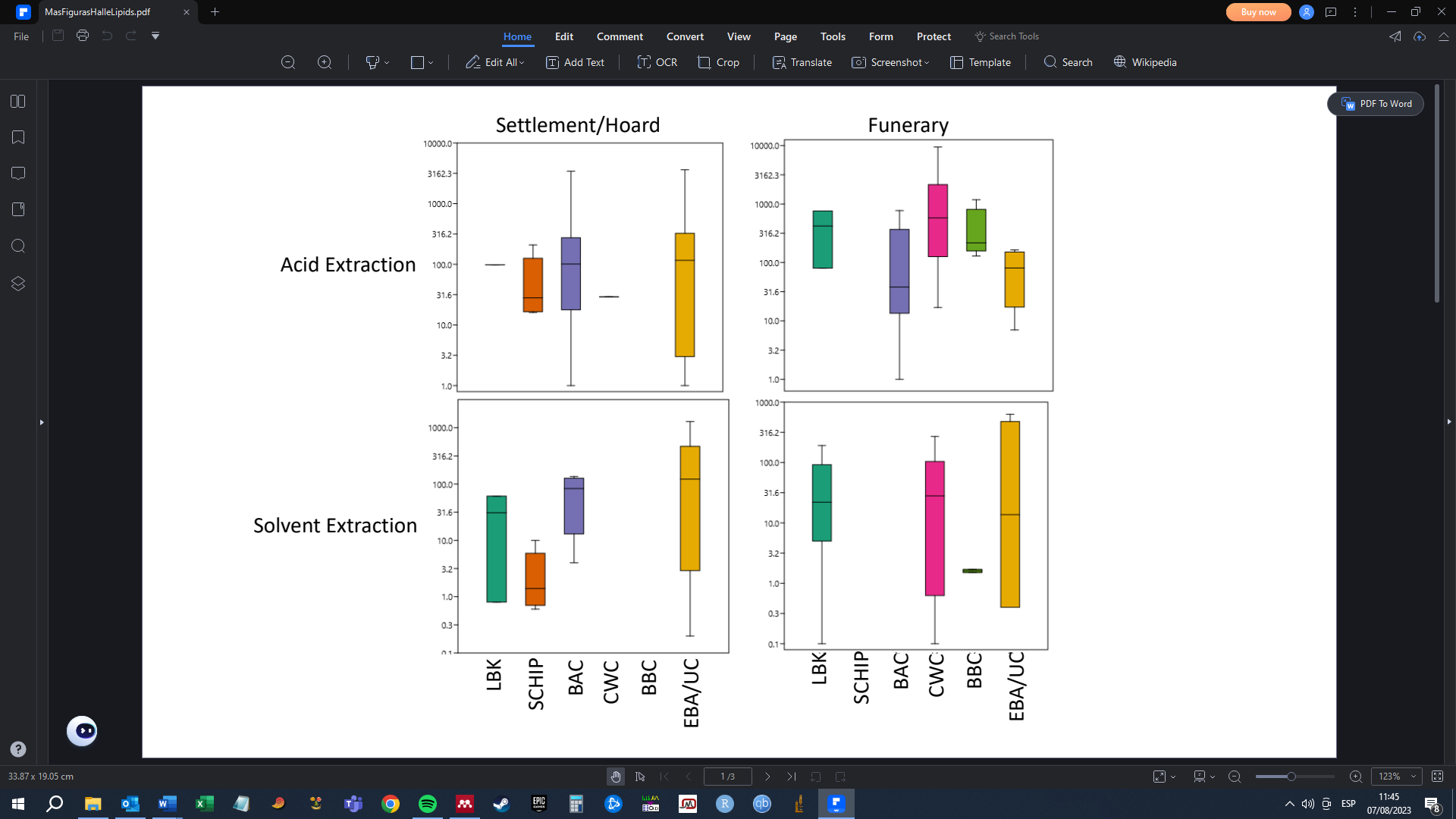


Due to these differences, a direct integration of DCM/MeOH and Acidified Methanol TLEs is not possible. For figure 3 in the main text, an alternative approach was used. Initially, samples were grouped by extraction type and ordered by rank from lowest to highest TLE within their group. The ranks were then normalised, thus giving the sample with the highest rank a value of 1 within each group. This approach allows the determination of samples with “high” or “low” quantities of lipids within the study by assigning them a value from 0 to 1.


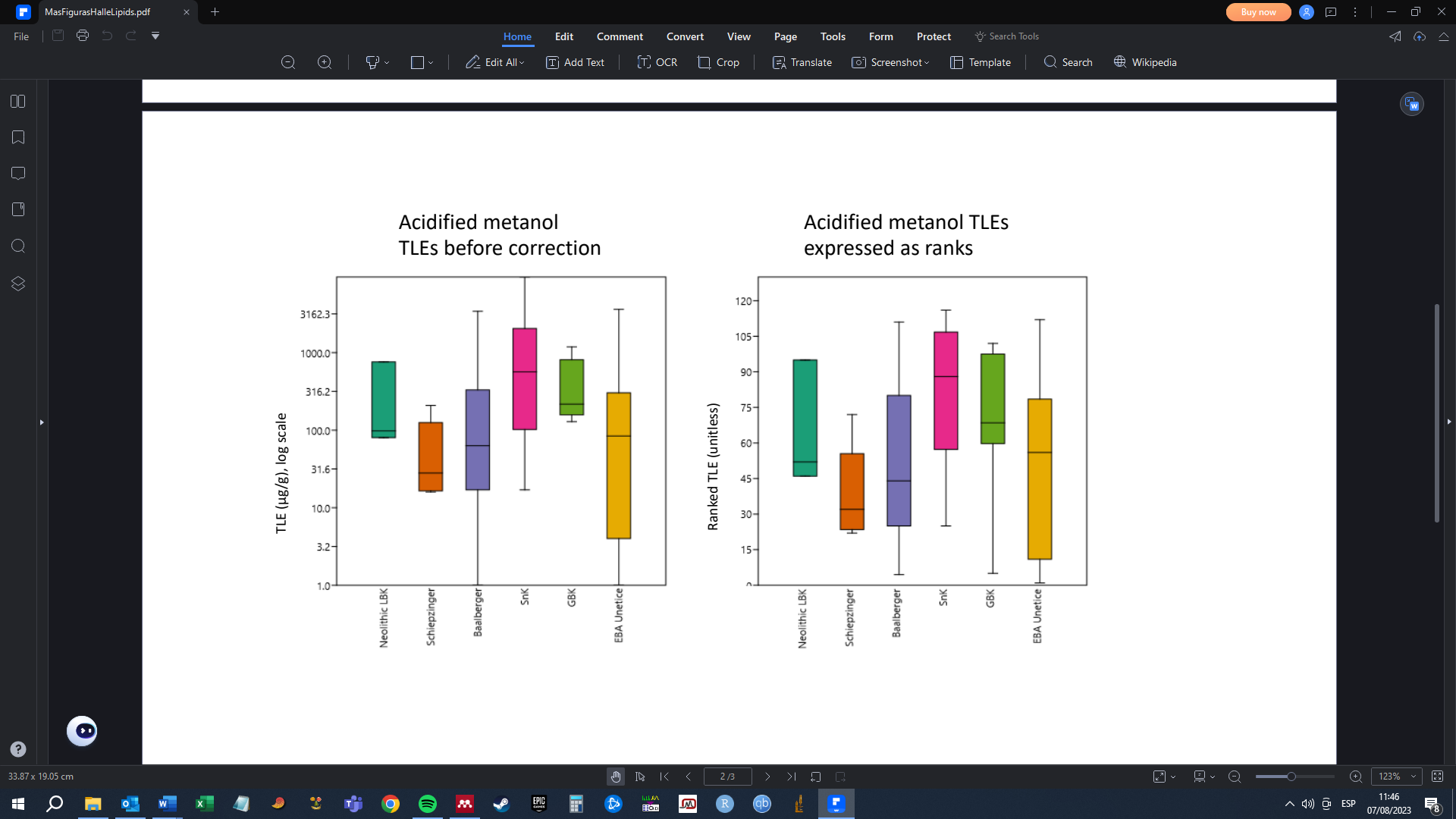


Comparisons of boxplots before and after correction show that the data treatment does not alter the results in a manner which may change the interpretation of the results. This approach rests on the fact that published studies from different teams and labs performing both acidified methanol and solvent extractions on powder from the same sherds show a positive correlation between their respective total lipid extracts expressed in microgrammes per grame of fat.


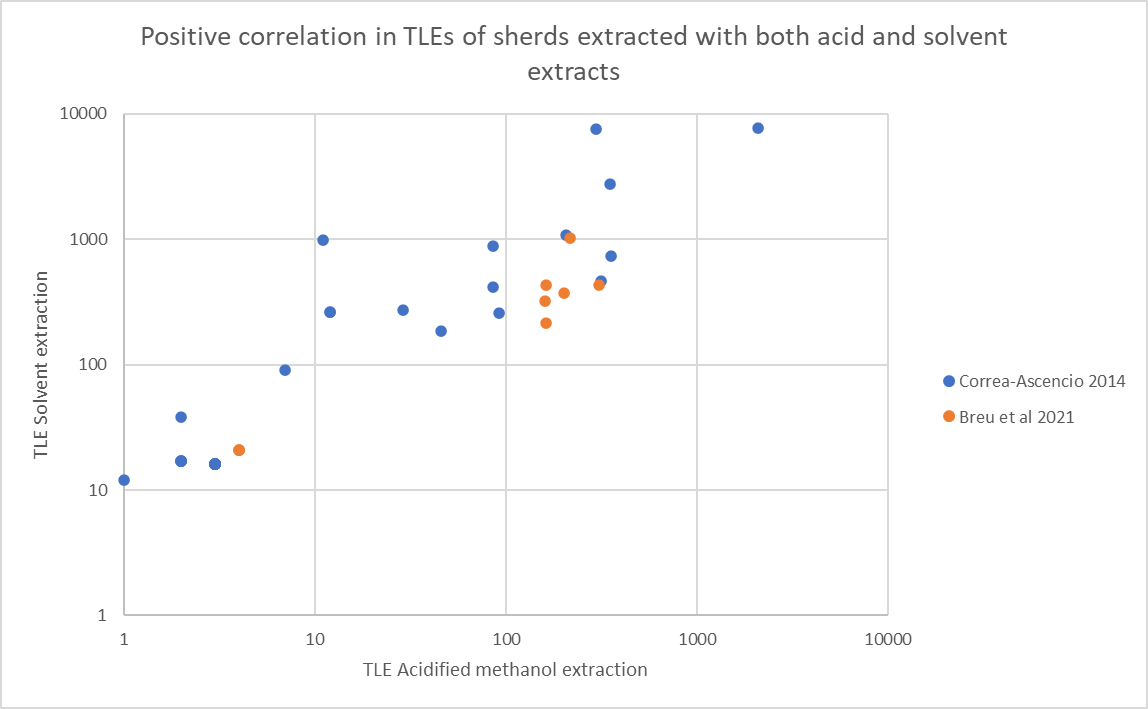


**3.4 Sampling and assessment and mitigation of contamination.**

Given that the storage areas of the State Office for Heritage Management and Archaeology of Saxony-Anhalt were affected by the Saale river floods of 2013, three undated pottery sherds from the undated sites of Elsteraue, Eisleben, and Preußlitz, located in apparently unaffected storage shelves were extracted with solvent and Acidified Methanol extractions to validate the preservation state of the ceramic assemblages prior to performing extensive analyses. The recovery of satisfactory amounts of lipid residues and limited to no contamination, akin to other published studies, informed further sampling decisions. Even though the isotopic values of palmitic and stearic acids could be analysed under the same requirements as all other samples in this study, they have not been included in the discussion as the fragments are not associated with a secure archaeological context.

Additionally, six highly contaminated samples correspond to vessels discovered in the “princely graves” of Leubingen and Helmsdorf, which were excavated in the 19^th^ century and stored since then in different magazines and museums. The lipids detected in samples HE-1, HE-2, HE-3, LE-4, LE-5 and
LE-6 showed high levels of phthalate plasticiser contamination, which could be caused by the way the vessels had been stored for more than two centuries. Signs of extensive contamination also included the presence of the unresolved complex mixture (UCM) in the gas chromatogram which could be attributed to a petrogenic source or the presence of traces of adhesives that were used to glue the different fragments of the pot.

Three samples from a large Late Bronze Age vessel (*amphora* BR-2) from Bernburg were chosen to test if residues were preserved at different levels of the inner surface (rim, upper body and lower body; subsamples: BR-2-1, BR-2-2 & BR-2-3). Unfortunately, when applying the DCM/MeOH extraction protocol, samples BR-2-2 and BR-2-3, taken from the rim, showed strong signs of contamination. The same extracted pottery powder was then submitted to an acidified methanol extraction, which yielded uncontaminated results. DCM/MeOH extractions have been used before to remove contaminants from archaeological samples [4] in the same manner as performed for the Bernburg vessel, although this approach has to be applied carefully as information from lipids not strongly bound to the pottery matrix may be lost.

**3.5 Diversity indices**

Borrowing from ecology, diversity indices such as Simpson’s D or H indices, Margalef’s richness index, or the Berger-Parker dominance value [5], were originally devised to study the richness and evenness of the distribution of species in different environments. Similarly, these same indices were retooled for archaeology [6–8], and are commonly employed in zooarchaeological and palaeobotanical studies [ex: 9,10] and frequently used in the analysis of prehistoric lithic tools, pottery, and artistic motifs [11,ex: 12–17], which demonstrates its versatility and applicability. In functional analysis, a certain tool type can be assumed to be specialised if it is employed for a single or a reduced group of tasks. Contrarily, general-purpose or multipurpose tools will be used for a multiplicity of tasks. Using the lipid residues as a proxy for the type of use given to a vessel type, the diversity and dominance indices can be used to explore the potential location of a specific shape in the specialised-generalised space.

In this paper, we have used Margalef’s richness index (hereafter the diversity index) [18] and the Berger-Parker dominance index (hereafter the dominance index) [19] to study the degree of specialisation of several pottery types. The formulas for these indices are as follows:

Margalef’s Diversity (D)

$$D=\frac{S-1}{\ln N}$$

Where S is the number of types present and N is the total number of studied cases.

Berger-Parker dominance:

$$BP=\frac{n_{max}}{N}$$

Where n_max_ is the number of cases belonging to the most frequent type and N is the total number of studied cases. This is, essentially, the percentual contribution of the most frequent type.

When calculated, the diversity and dominance indices tend to be inversely correlated. Thus, high specialisation would present lower diversity and higher dominance indices while highly multipurpose tools will tend to present higher diversity and lower dominance indices. As a general rule, we consider diversity and dominance indices to be low when they oscillate around 0.5 or lower.

The application of these indices, however, is not without its limitations if the potential range of types is unknown. Thus, small sample sizes combined with numerous types may result in a lack of precision. Additionally, archaeological applications must also take into account the potential effects of the loss of information due to post-depositional effects. Interpreting small variations in these indices is therefore not generally advised.

In the case of this study, four strategies have been used to mitigate precision problems: (1) only pottery shapes with five or more cases have been evaluated, we argue this is adequate as the study contemplated five possible different residue types, (2) absence of lipids has been considered a valid strategy to take into account potentially undetected types due to degradation or uses not involving fatty-rich substances, (3) we have abstained from interpreting minor differences in these indices, (4) a jackknife cross-validation has been performed to test for biases and assess the variances of our estimates:

| **Vessel types** | **Calculated Dominance** | **Jackknife cross-validation** | | **Calculated Diversity** | **Jackknife cross-validation** | |
| --- | --- | --- | --- | --- | --- | --- |
|  |  | **Mean** | **St. Dev.** |  | **Mean** | **St. Dev.** |
| **SCHIP Pots** | 0.43 | 0.49 | 0.03 | 1.03 | 1.03 | 0.20 |
| **BAC Cups** | 0.89 | 0.88 | 0.04 | 0.46 | 0.43 | 0.15 |
| **CWC Beakers** | 0.43 | 0.49 | 0.03 | 1.03 | 1.03 | 0.20 |
| **CWC Cups** | 0.50 | 0.50 | 0.07 | 1.44 | 1.41 | 0.22 |
| **CWC Amphorae** | 0.88 | 0.88 | 0.05 | 0.48 | 0.45 | 0.17 |
| **BBC Beakers** | 0.86 | 0.86 | 0.06 | 0.51 | 0.48 | 0.20 |
| **UC Classical cups** | 0.54 | 0.54 | 0.04 | 1.17 | 1.16 | 0.11 |
| **UC Pithoi** | 0.40 | 0.48 | 0.04 | 1.24 | 1.29 | 0.29 |
| **UC Pots** | 0.43 | 0.49 | 0.03 | 1.03 | 1.03 | 0.20 |

**3.6 Additional mass spectra from sterols in sample PR-13**

NIST reference mass spectra for Cholestan-3-ol TMS

Detected mass spectra at Rt: 41.1 in sample PR-13

107

75

Detected mass spectra at Rt: 43.0 in sample PR-13


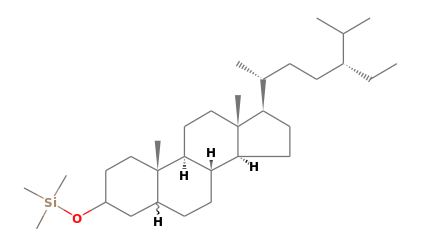

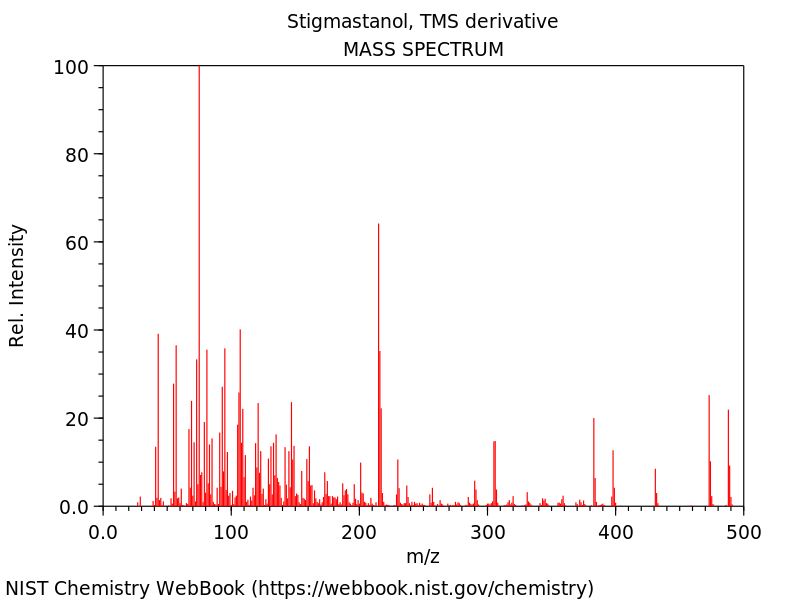


107

305

398

431

473

488

215

NIST reference mass spectra for Stigmastanol TMS

Source: https://webbook.nist.gov/cgi/cbook.cgi?ID=C55493851&Mask=200

**3.6 Study of lipid preservation and vessel parts sampled given the low lipid yields at Pömmelte**

In light of the low lipid yields at Pömmelte, an assessment of a potential bias originating from the vessel parts sampled was performed. The study of which vessel part to sample to increase the probabilities of detecting a residue is complex and mediated by several factors. Different activities may result in lipids being deposited differently in the vessel. For example, boiling will make lipids float and thus they will be deposited closer to the upper part or the rim [20,21]. Ethnoarchaeological studies have used this to establish which percentage of the vessel was repeatedly filled and to determine the vessel’s “filling line” [22]. Alternatively, frying or cooking without water could concentrate fats at the base or closer to the lower parts of the vessel, resulting in a different deposition pattern. As different shapes also facilitate the practice of different activities, they may present specific lipid depositional patterns. Ware types and the presence of calcite and/or metal ions in the clay is another factor to take into account and supported by some experimental research [23,24]. Furthermore, as contamination tends to be more superficial but ancient lipids have had more time to penetrate to the core of the vessel, the surface and depth of the place from which the 1 or 2 grammes of sample are drilled could also potentially influence the results [25].

In consequence, clear associations linking the upper or the lower part of the vessel to higher or lower quantities of lipids are challenging and infrequent. However, the following tables and graphs aim to explore whether lipid yields for the studied vessels in Central Germany can be explained by the vessel part that was sampled.

Solvent extracts (DCM/MeOH extractions)

| Sampled part | N | Median amount of fats | Standard Deviation of the TLE |
| --- | --- | --- | --- |
| Lower | 7 | 69 μg/g | 63 μg/g |
| Central | 43 | 4 μg/g | 99 μg/g |
| Upper | 14 | 56 μg/g | 343 μg/g |


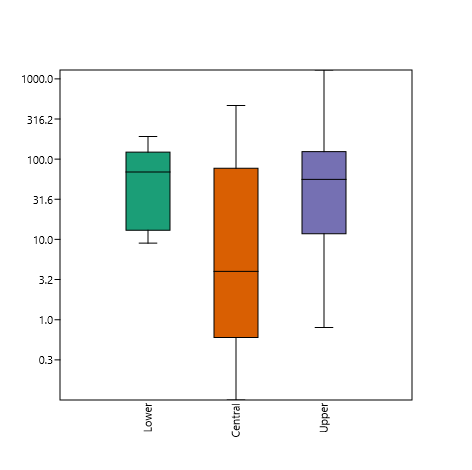
Across our study, samples taken from lower or upper vessel parts do not seem to present higher quantities of lipids. Note how the standard deviations are really high, indicating that regardless of the sampled part, it is possible to find vessels with high quantities of fats, or almost no fats at all.

Overall, for solvent extracts, the sampled vessel part has little to no explanatory power in the actual recovery of a positive lipid extract.

Acidified methanol extracts

| Sampled part | N | Median amount of fats | Standard Deviation |
| --- | --- | --- | --- |
| Lower | 38 | 71 μg/g | 1527 μg/g |
| Central | 40 | 310 μg/g | 1578 μg/g |
| Upper | 36 | 90 μg/g | 1337 μg/g |


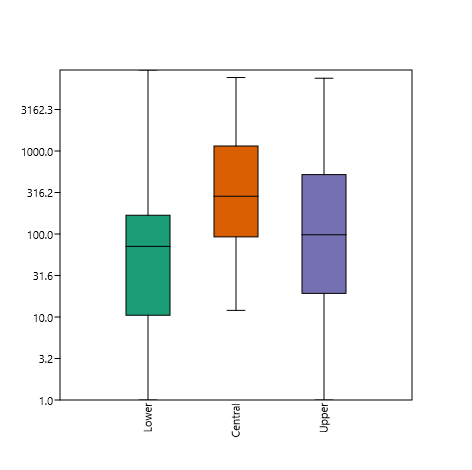


Samples analysed with the acidified methanol procedure show a trend akin to the solvent extracts, no vessel part seems to yield significantly higher lipid quantities than the others.

Again, the standard deviation of the total lipid extracts is two orders of magnitude higher than the median, meaning that almost any result from any vessel part can be expected.

The case of Pömmelte

| Sampled part | N | Median amount of fats | Standard Deviation |
| --- | --- | --- | --- |
| Lower | 18 | 14 μg/g | 197 μg/g |
| Upper | 10 | 34 μg/g | 361 μg/g |


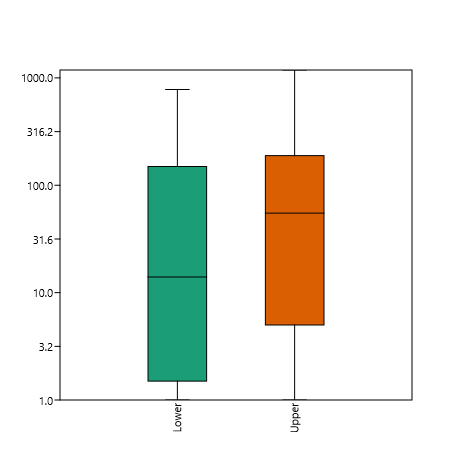


In our study, Pömmelte has been shown to present the worst preservation rates (percentage of samples with a TLE higher than 5 μg/g), “only” 57%, 16 out of 28 samples. All samples were extracted with the Acidified Methanol procedure and, again, no major differences exist between lower and upper vessel parts and the standard deviations are also really high. It should be noted, however, that the preservation is uneven between periods. Baalberge and Únětice vessels present rates of 40% and 35% respectively while Bell Beakers have a 100% preservation rate.

In consequence, it seems that the low preservation rate is a specific phenomenon of Pömmelte, but not appearing in all periods, that could be explained by a multiplicity of factors, including the possibility that vessels that were not used to contain lipid-rich products, or the geomorphological environment in which it they were placed. However, it should be noted that there is also a competing explanation: some of the vessels may not have contained products that would leave a lipid residue, or some vessels contained such large amounts (e.g., Bell Beakers) that survival in an inadequate soil was possible.

**3.7 References:**

1. Evershed RP, Heron C, Goad LJ. Analysis of organic residues of archaeological origin by high-temperature gas chromatography and gas chromatography-mass spectrometry. Analyst. 1990;115: 1339–1342. doi:10.1039/AN9901501339

2. Correa-Ascencio M, Evershed RP. High throughput screening of organic residues in archaeological potsherds using direct acidified methanol extraction. Anal Methods. 2014;6: 1330–1340. doi:10.1039/C3AY41678J

3. Papakosta V, Smittenberg RH, Gibbs K, Jordan P, Isaksson S. Extraction and derivatization of absorbed lipid residues from very small and very old samples of ceramic potsherds for molecular analysis by gas chromatography-mass spectrometry (GC-MS) and single compound stable carbon isotope analysis by gas chromatography-combustion-isotope ratio mass spectrometry (GC-C-IRMS). Microchem J. 2015;123: 196–200. doi:10.1016/j.microc.2015.06.013

4. Colonese AC, Farrell T, Lucquin A, Firth D, Charlton S, Robson HK, et al. Archaeological bone lipids as palaeodietary markers. Rapid Commun Mass Spectrom. 2015;29: 611–8. doi:10.1002/rcm.7144

5. Magurran AE. Ecological Diversity and Its Measurement. Dordrecht: Springer Netherlands; 1988. doi:10.1007/978-94-015-7358-0

6. Rice PM. Specialization, standardization, and diversity: a retrospective. Ceram Leg Anna O Shepard. 1991; 257–279.

7. Eren MI, Buchanan B. Defining and Measuring Diversity in Archaeology: Another Step Toward an Evolutionary Synthesis of Culture. Berghahn Books; 2022.

8. Cruz-Uribe K. The use and meaning of species diversity and richness in archaeological faunas. J Archaeol Sci. 1988;15: 179–196. doi:10.1016/0305-4403(88)90006-4

9. de Vareilles A, Bouby L, Jesus A, Martin L, Rottoli M, Vander Linden M, et al. One sea but many routes to Sail. The early maritime dispersal of Neolithic crops from the Aegean to the western Mediterranean. J Archaeol Sci Reports. 2020;29: 102140. doi:10.1016/j.jasrep.2019.102140

10. Gulyás S, Sümegi P. Farming and/or foraging? New environmental data to the life and economic transformation of Late Neolithic tell communities (Tisza Culture) in SE Hungary. J Archaeol Sci. 2011;38: 3323–3339. doi:10.1016/j.jas.2011.07.019

11. Lull V, Micó R, Palomar B, Rihuete C, Risch R. Cerámica talayótica: la producción alfarera mallorquina entre ca 900 y 550 antres de nuestra era. Col·lecció d’arqueoecologia social mediterrània. Barcelona: Edicions Bellaterra; 2008.

12. Breu A. El fenomen cardial al noroest peninsular: una visió des de la perspectiva de l’estil. Universitat Autònoma de Barcelona. 2014.

13. Fragnoli P. Re-assessing the notion(s) of craft standardization through diversity statistics: A pilot study on Late Chalcolithic pottery from Arslantepe in Eastern Anatolia. PLoS ONE. 2021. doi:10.1371/journal.pone.0245660

14. Campbell S, Healey E. Diversity in obsidian use in the prehistoric and early historic Middle East. Quat Int. 2018;468: 141–154. doi:10.1016/j.quaint.2017.09.023

15. Henry DO, Mraz V. Lithic economy and prehistoric human behavioral ecology viewed from southern Jordan. J Archaeol Sci Reports. 2020;29: 102089. doi:10.1016/j.jasrep.2019.102089

16. Reckin R, Todd LC. Illuminating high elevation seasonal occupational duration using diversity in lithic raw materials and tool types in the greater Yellowstone Ecosystem, USA. J Anthropol Archaeol. 2020;57: 101119. doi:10.1016/j.jaa.2019.101119

17. Cardillo M, Alberti J. Explaining the diversification of lithic projectile points from the northern Patagonian coast (Argentina) during the Holocene using phylogenetic and comparative methods. J Archaeol Sci Reports. 2023;51: 104144. doi:10.1016/j.jasrep.2023.104144

18. Margalef R. Information theory in Ecology. Gen Syst. 1958; 36–71.

19. Berger WH, Parker FL. Diversity of Planktonic Foraminifera in Deep-Sea Sediments. Science (80- ). 1970;168: 1345–1347. doi:10.1126/science.168.3937.1345

20. Charters S, Evershed RP, Goad LJ, Leyden A, Blinkhorn PW, Denham V. Quantification and distribution of lipid in archaeological ceramics: implications for sampling potsherds for organic residue analysis and the classification of vessel use. Archaeometry. 1993;35: 211–223. doi:10.1111/j.1475-4754.1993.tb01036.x

21. Charters S, Evershed RP, Quye A, Blinkhorn PW, Reeves V. Simulation Experiments for Determining the Use of Ancient Pottery Vessels: the Behaviour of Epicuticular Leaf Wax During Boiling of a Leafy Vegetable. J Archaeol Sci. 1997;24: 1–7. doi:http://dx.doi.org/10.1006/jasc.1995.0091

22. Drieu L, Regert M, Mazuy A, Vieugué J, Bocoum H, Mayor A. Relationships Between Lipid Profiles and Use of Ethnographic Pottery: an Exploratory Study. J Archaeol Method Theory. 2022. doi:10.1007/s10816-021-09547-1

23. Hammann S, Scurr DJ, Alexander MR, Cramp LJE. Mechanisms of lipid preservation in archaeological clay ceramics revealed by mass spectrometry imaging. Proc Natl Acad Sci U S A. 2020;117: 14688–14693. doi:10.1073/pnas.1922445117

24. Raven AM, van Bergen PF, Stott AW, Dudd SN, Evershed RP. Formation of long-chain ketones in archaeological pottery vessels by pyrolysis of acyl lipids. J Anal Appl Pyrolysis. 1997;40–41: 267–285. doi:10.1016/S0165-2370(97)00036-3

25. Stern B, Heron C, Serpico M, Bourriau J. A comparison of methods for establishing fatty acid concentration gradients across potsherds: A case study using late bronze age canaanite amphorae. Archaeometry. 2000;42: 399–414. doi:10.1111/j.1475-4754.2000.tb00890.x

3.8 **Credits for figures in the text and in the supplementary materials 1**

**Figures text:**

| **Fig No** | **Credit** |
| --- | --- |
| Fig 1 | base map: N. Seeländer, State Office for Heritage Management and Archaeology Saxony-Anhalt after H. Behrens; mapping: A. Swieder, State Office for Heritage Management and Archaeology Saxony-Anhalt |
| Fig 2 | State Office for Heritage Management and Archaeology Saxony-Anhalt |
| Fig 3 | A. Breu |
| Fig 4 | A. Breu |
| Fig 5 | A. Breu |
| Fig 6 | A. Breu |
| Fig 7 | A. Breu |
| Fig 8 | A. Breu |
| Fig 9 | A. Breu |
| Fig 10 | A. Breu |
| Fig 11 | A. Breu |
| Fig 12 | A. Breu & R. Risch |
| Fig 13 | Layout: R. Risch; drawings see Supp. 1 |
| Fig 14 | A. Breu |

**Supplement 1**

Graphic Design & Layout: B. Janzen, State Office for Heritage Management and Archaeology Saxony-Anhalt and R. Risch, Universitat Autònoma de Barcelona.

| **Page no.** | **Arch ID vessel (HK-Nr.)** | **Sample no.** | **Copyright** |
| --- | --- | --- | --- |
| 1 | 10646:634:9 | HA-1 | M. Eguíluz, State Office for Heritage Management and Archaeology Saxony-Anhalt |
|  | 10756:94:2c | HA-12 | M. Eguíluz, State Office for Heritage Management and Archaeology Saxony-Anhalt |
|  | 10757:11040:4c | HA-7 | M. Eguíluz, State Office for Heritage Management and Archaeology Saxony-Anhalt |
|  | 10757:11040:11f+d | HA-8 | M. Eguíluz, State Office for Heritage Management and Archaeology Saxony-Anhalt |
| 2 | 10757:11040:41a | HA-9 | M. Eguíluz, State Office for Heritage Management and Archaeology Saxony-Anhalt |
|  | 10757:11094:35 | HA-10 | M. Eguíluz, State Office for Heritage Management and Archaeology Saxony-Anhalt |
| 3 | 10757:11058:1a | HA-14 | M. Eguíluz, State Office for Heritage Management and Archaeology Saxony-Anhalt |
|  | 10757:11058:2a | HA-13 | M. Eguíluz, State Office for Heritage Management and Archaeology Saxony-Anhalt |
| 4 | 9495:1031:40a | LI-1 | M. Reuter, State Office for Heritage Management and Archaeology Saxony-Anhalt |
|  | 9495:1031:41a | LI-2 | M. Reuter, State Office for Heritage Management and Archaeology Saxony-Anhalt |
| 5 | 9495:1034:35a | LI-3 | M. Reuter, State Office for Heritage Management and Archaeology Saxony-Anhalt |
|  | 9495:2030:22 | LI-4 | M. Reuter, State Office for Heritage Management and Archaeology Saxony-Anhalt |
|  | 9495:2030:31 | LI-5 | M. Reuter, State Office for Heritage Management and Archaeology Saxony-Anhalt |
| 6 | 12:114 | BE-1 | Weber 2015, tab. 11,4 |
|  | 14:1068 | BE-2 | Weber 2015, tab. 16,1 |
| 7 | 14:1146 | BE-8 | Weber 2015, tab. 19,4 |
|  | 14:1054 | BE-4 | Weber 2015, tab. 15,7 |
| 8 | 13:462 | BE-3 | Weber 2015, tab. 13,6 |
|  | 14:1054 | BE-9 | Weber 2015, tab. 17,1 |
| 9 | 1-95:1593 | BE-5 | Weber 2015, tab. 4,6 |
|  | 13:461 | BE-6 | Weber 2015, tab. 12,3 |
|  | 1-96:1547 | BE-7 | Weber 2015, tab. 5,1 |
| 10 | 2002:2481a | QU-40 | Petzschmann 2006, 158 Abb. 4,3 |
|  | 2002:2481b | QU-41 | Petzschmann 2006, 158 Abb. 4,1 |
|  | 2002:2481c | QU-42 | Petzschmann 2006, 158 Abb. 4,2 |
|  | 2002:2483a | QU-46 | Petzschmann 2006, 162 Abb. 8,2 |
|  | 2002:2483b | QU-47 | Petzschmann 2006, 162 Abb. 8,1 |
|  | 2002:2484a | QU-48 | Petzschmann 2006, 164 Abb. 10,2 |
|  | 2002:2484b | QU-49 | Petzschmann 2006, 164 Abb. 10,1 |
| 11 | 2002:2482a | QU-43 | Petzschmann 2006, 160 Abb. 6a,1 |
|  | 2002:2482b | QU-44 | Petzschmann 2006, 160 Abb. 6a,2 |
|  | 2002:2482c | QU-45 | Petzschmann 2006, 161 Abb. 6b |
| 12 | 13476:2970:1a | PO-14 | A. Hermann/K. N. Rauh, State Office for Heritage Management and Archaeology Saxony-Anhalt |
|  | 13476:2970:2a | PO-15 | K. N. Rauh, State Office for Heritage Management and Archaeology Saxony-Anhalt |
|  | 12820:25741a | PO-13 | K. N. Rauh, State Office for Heritage Management and Archaeology Saxony-Anhalt |
|  | 13476:5501:2a | PO-20 | A. Hermann, State Office for Heritage Management and Archaeology Saxony-Anhalt |
|  | 13476:5501:2b | PO-19 | K. N. Rauh, State Office for Heritage Management and Archaeology Saxony-Anhalt |
| 13 | 2510:7616:35 | PR-9 | L. Kaudelka, State Office for Heritage Management and Archaeology Saxony-Anhalt |
|  | 2510:7616:43 | PR-10 | L. Kaudelka, State Office for Heritage Management and Archaeology Saxony-Anhalt |
|  | 2510:5068:7 | PR-5 | State Office for Heritage Management and Archaeology Saxony-Anhalt |
|  | 2510:5068:8 | PR-6 | State Office for Heritage Management and Archaeology Saxony-Anhalt |
| 14 | 2510:7868:34a | PR-17 | State Office for Heritage Management and Archaeology Saxony-Anhalt |
|  | 2510:7868:33a | PR-16 | State Office for Heritage Management and Archaeology Saxony-Anhalt |
| 15 | 2510:7625:16a | PR-14 | State Office for Heritage Management and Archaeology Saxony-Anhalt |
|  | 2510:7625:17a | PR-15 | State Office for Heritage Management and Archaeology Saxony-Anhalt |
|  | 2510:8821:11 | PR-18 | L. Kaudelka, State Office for Heritage Management and Archaeology Saxony-Anhalt |
| 16 | 2510:7618:16 | PR-11 | S. Neufeldt, State Office for Heritage Management and Archaeology Saxony-Anhalt |
|  | 2510:7618:17 | PR-12 | S. Neufeldt, State Office for Heritage Management and Archaeology Saxony-Anhalt |
|  | 2510:7618:18 | PR-13 | S. Neufeldt, State Office for Heritage Management and Archaeology Saxony-Anhalt |
| 17 | 2510:5043:23 | PR-1 | L. Kaudelka, State Office for Heritage Management and Archaeology Saxony-Anhalt |
|  | 2510:5043:24 | PR-2 | L. Kaudelka, State Office for Heritage Management and Archaeology Saxony-Anhalt |
|  | 2510:5043:25 | PR-3 | L. Kaudelka, State Office for Heritage Management and Archaeology Saxony-Anhalt |
|  | 2510:5043:27 | PR-4 | L. Kaudelka, State Office for Heritage Management and Archaeology Saxony-Anhalt |
| 18 | 2671:25815:38 | OE-7 | State Office for Heritage Management and Archaeology Saxony-Anhalt |
|  | 3125:1581:7 | BR-1 | State Office for Heritage Management and Archaeology Saxony-Anhalt |
|  | 13476:5638:1a | PO-32 | A. Hermann/K. N. Rauh, State Office for Heritage Management and Archaeology Saxony-Anhalt |
| 19 | 2365:5351:23 | WE-1 | State Office for Heritage Management and Archaeology Saxony-Anhalt |
|  | 2365:5351:49a | WE-2 | L. Kaudelka, State Office for Heritage Management and Archaeology Saxony-Anhalt |
|  | 2365:5351:50a | WE-3 | L. Kaudelka, State Office for Heritage Management and Archaeology Saxony-Anhalt |
| 20 | 2671:25645:70 + 75 | OE-5 + OE-6 | L. Kaudelka, State Office for Heritage Management and Archaeology Saxony-Anhalt |
|  | 2671:25645:104 | OE-4 | L. Kaudelka, State Office for Heritage Management and Archaeology Saxony-Anhalt |
| 21 | 2671:25355:26 | OE-1 | S. Neufeldt, State Office for Heritage Management and Archaeology Saxony-Anhalt |
|  | 2671:25355:27 | OE-2 | State Office for Heritage Management and Archaeology Saxony-Anhalt |
|  | 2671:25355:28 | OE-3 | S. Neufeldt, State Office for Heritage Management and Archaeology Saxony-Anhalt |
| 22 | 12820:1750:2a | PO-37 | M. Eguíluz, State Office for Heritage Management and Archaeology Saxony-Anhalt |
|  | 12820:1742:1a | PO-38 | M. Eguíluz, State Office for Heritage Management and Archaeology Saxony-Anhalt |
|  | 12820:1567:1 | PO-34 | M. Eguíluz, State Office for Heritage Management and Archaeology Saxony-Anhalt |
| 23 | 12820:1454:1a | PO-35 | M. Eguíluz, State Office for Heritage Management and Archaeology Saxony-Anhalt |
|  | 12820:1866:2a | PO-36 | K. N. Rauh, State Office for Heritage Management and Archaeology Saxony-Anhalt |
|  | 13476:4207:1a | PO-18 | A. Hermann/K. N. Rauh, State Office for Heritage Management and Archaeology Saxony-Anhalt |
| 24 | 13476:5643:4a | PO-21 | A. Hermann/K. N. Rauh, State Office for Heritage Management and Archaeology Saxony-Anhalt |
|  | 13476:4200:2a | PO-17 | A. Hermann/K. N. Rauh, State Office for Heritage Management and Archaeology Saxony-Anhalt |
| 25 | 2510:5427:21 | PR-7 | L. Kaudelka, State Office for Heritage Management and Archaeology Saxony-Anhalt |
|  | 2510:5427:22 | PR-8 | L. Kaudelka, State Office for Heritage Management and Archaeology Saxony-Anhalt |
|  | 13476:3298:1 | PO-16 | K. N. Rauh, State Office for Heritage Management and Archaeology Saxony-Anhalt |
|  | 13476:2702:1a | PO-39 | K. N. Rauh, State Office for Heritage Management and Archaeology Saxony-Anhalt |
| 26 | 12820:2372:1a | PO-12 | K. N. Rauh, State Office for Heritage Management and Archaeology Saxony-Anhalt |
|  | 13476:2974:1a | PO-33 | K. N. Rauh, State Office for Heritage Management and Archaeology Saxony-Anhalt |
| 27 | 13476:4522:2b | PO-29 | A. Hermann/K. N. Rauh, State Office for Heritage Management and Archaeology Saxony-Anhalt |
|  | 13476:4522:3b | PO-23 | A. Hermann/K. N. Rauh, State Office for Heritage Management and Archaeology Saxony-Anhalt |
|  | 13476:4522:8a | PO-30 | K. N. Rauh, State Office for Heritage Management and Archaeology Saxony-Anhalt |
|  | 13476:4522:6a | PO-31 | A. Hermann/K. N. Rauh, State Office for Heritage Management and Archaeology Saxony-Anhalt |
|  | 13476:4522:7a | PO-26 | K. N. Rauh, State Office for Heritage Management and Archaeology Saxony-Anhalt |
| 28 | 13476:4522:2a | PO-27 | A. Hermann/K. N. Rauh, State Office for Heritage Management and Archaeology Saxony-Anhalt |
|  | 13476:4522:3a | PO-28 | A. Hermann/K. N. Rauh, State Office for Heritage Management and Archaeology Saxony-Anhalt |
|  | 13476:4522:5a | PO-22 | A. Hermann/K. N. Rauh, State Office for Heritage Management and Archaeology Saxony-Anhalt |
| 29 | 13476:4522:7b | PO-24 | K. N. Rauh, State Office for Heritage Management and Archaeology Saxony-Anhalt |
|  | 13476:4522:4a | PO-25 | A. Hermann/K. N. Rauh, State Office for Heritage Management and Archaeology Saxony-Anhalt |
|  | 3615:20147:4a | KL-2 | Revert 2019, Taf. 2,2 |
| 30 | 3615:40168:36a | KL-10 | G. Thies, State Office for Heritage Management and Archaeology Saxony-Anhalt |
|  | 3615:40168:3a | KL-4 | V. Paškauskas, State Office for Heritage Management and Archaeology Saxony-Anhalt |
| 31 | 3615:40168:8a | KL-11 | State Office for Heritage Management and Archaeology Saxony-Anhalt |
|  | 3615:40168:15a | KL-5 | V. Paškauskas, State Office for Heritage Management and Archaeology Saxony-Anhalt |
|  | 3615:30137:1a | KL-6 | V. Paškauskas, State Office for Heritage Management and Archaeology Saxony-Anhalt |
| 32 | 3615:40180:1a | KL-7 | State Office for Heritage Management and Archaeology Saxony-Anhalt |
|  | 3615:30115:2 | KL-1 | G. Thies, State Office for Heritage Management and Archaeology Saxony-Anhalt |
|  | 3615:20147:2a | KL-3 | Revert 2019, Taf. 4,4 |
| 33 | 10734:129:6 | ES-1 | J. Schüler, State Office for Heritage Management and Archaeology Saxony-Anhalt |
|  | 10734:151:2 | ES-2 | J. Schüler, State Office for Heritage Management and Archaeology Saxony-Anhalt |
| 34 | 11243:330:2a | ME-5 | J. Schüler, State Office for Heritage Management and Archaeology Saxony-Anhalt |
|  | 2607:290:592 | OE-10 | S. Neufeldt, State Office for Heritage Management and Archaeology Saxony-Anhalt |
| 35 | 2671:25960:14 | OE-8 | State Office for Heritage Management and Archaeology Saxony-Anhalt |
|  | 2671:25960:15 | OE-9 | State Office for Heritage Management and Archaeology Saxony-Anhalt |
| 36 | 3665:5524:106 | BR-2 | State Office for Heritage Management and Archaeology Saxony-Anhalt |
|  | 3665:5524:110a | BR-3 | State Office for Heritage Management and Archaeology Saxony-Anhalt |
